# A Scoping Review of the Evidence for the Medicinal Use of Natural Honey in Animals

**DOI:** 10.3389/fvets.2020.618301

**Published:** 2021-01-18

**Authors:** Nadine A. Vogt, Ellen Vriezen, Andrea Nwosu, Jan M. Sargeant

**Affiliations:** Department of Population Medicine, Ontario Veterinary College, University of Guelph, Guelph, ON, Canada

**Keywords:** biomedical, canine, feline, treatment efficacy, equine, *Apis*, honey, veterinary

## Abstract

Honey has a history of medicinal use that predates written records. In recent decades, there has been renewed interest in the use of honey in human medicine, particularly for the treatment of burns and other wounds. Several recent systematic reviews in the human literature have demonstrated the efficacy of honey in the treatment of a number of conditions, including burns, wounds and oral mucositis. The goal of this scoping review was to describe the nature and extent of the current body of evidence addressing the medicinal use of natural honey and/or its derivatives in animals. Although the focus of this review was the veterinary literature, all animal species except insects and humans were eligible, including animals used for biomedical research. Electronic databases searched were MEDLINE, CAB Abstracts, AGRICOLA, Web of Science Core Collection, and Web of Science SciELO Citation Index. A total of 397 articles reporting 436 primary research studies were included in this review. The majority of the articles were biomedical research articles (*n* = 350); fewer veterinary research articles were identified (*n* = 47). Apart from one systematic review, all biomedical studies were challenge trials. Most veterinary studies were case reports/series (*n* = 23), followed by challenge trials (*n* = 18) and controlled trials (*n* = 8). The animal species examined within veterinary articles consisted primarily of dogs, horses, cats and cattle, whereas the majority of biomedical research articles examined rats and mice. Wound healing was the most common indication examined; other indications examined included the prevention or treatment of gastric ulcers, bacterial and parasitic infections, toxic exposures, metabolic conditions (e.g., diabetes) and neoplasia. The majority of interventions consisted of non-medical grade honey (*n* = 412/436), followed by medical-grade honey (*n* = 29/436) and derivatives of natural honey (*n* = 9/436). With much of the current veterinary literature consisting of case reports and case series, high-quality primary veterinary research in the form of controlled trials or challenge trials is needed to advance this field, as well as to provide sound data for evidence-based assessments of the efficacy of honey in clinical veterinary practise.

## Introduction

Throughout history, honey has been used as a medicine in the treatment of a range of ailments. In recent decades, there has been a renewed interest in the use of honey as a therapeutic agent, especially for burns, infected wounds and wounds refractory to conventional treatments (1–3). Manuka honey, in particular, is now widely recognised for its ability to eliminate problematic multi-drug resistant pathogens (e.g., methicillin-resistant *Staphylococcus aureus*, multi-drug resistant *Pseudomonas aeruginosa*) for which few to no effective antibiotics currently exist (4, 5). Honey exhibits a broad range of medicinal properties, including antibacterial, anti-inflammatory, anti-mutagenic and anti-proliferative properties (6–8). These medicinal properties of honey have been attributed to some of its over 200 biologically active compounds. Although predominantly composed of sugars, honey also contains a variety of vitamins, minerals, enzymes as well as a diversity of plant-derived compounds, polyphenols, which are known to confer potent antioxidant activity (2).

There is a growing body of human literature examining the use of honey as a therapeutic for indications besides wound healing, including gastrointestinal disorders (e.g., gastritis, duodenitis), ocular conditions (e.g., corneal burns, keratitis) and metabolic syndromes (e.g., diabetes, hypertension) (2, 9, 10). In an effort to summarise existing literature and critically evaluate the quality of available scientific evidence, systematic reviews have been used to evaluate the efficacy of honey in treating burns and wound healing in humans (3, 11–13). Systematic reviews evaluating honey for human applications have reported limitations due to limited available primary research, small sample sizes and poor quality of research (3, 14, 15). Several systematic reviews and meta-analyses or network meta-analyses, however, provide some evidence that honey is superior to conventional treatments for certain conditions, namely, burns (12, 16), and oral mucositis induced by chemotherapy or radiation therapy (17).

In recent years, veterinary medicine has experienced a similar recrudescence of interest in honey as a therapeutic (18–20), and yet there is currently little information available in the veterinary literature regarding the extent of scientific investigation of honey for different therapeutic purposes in different animal species. Given the important differences in the physiology and anatomy between animal species (e.g., the propensity of horses to produce excessive granulation tissue in wounds), a species-specific approach is needed when evaluating the efficacy of potential therapeutics such as honey. Systematic reviews and meta-analyses are an important tool for addressing questions of efficacy; however, they require a sufficient body of existing literature. Another tool for research synthesis that is often considered a useful prerequisite for a systematic review is a scoping review (21). The primary purpose of a scoping review is to identify and map the existing literature on a given topic. Scoping reviews provide an overview of the type(s) of available evidence in a given research field and can be used to identify research gaps, as well as specific subject areas that contain sufficient existing literature for systematic reviews. In contrast with the narrow scope of a systematic review question that assesses the efficacy of a treatment for a specific condition in a single species, scoping reviews address broader research questions about the nature and extent of existing evidence (21). Scoping reviews do not typically provide results from included studies or include assessments of study quality; however, they are considered a rigorous, transparent, and replicable methodology for charting the literature (21). The objective of this scoping review was to examine the extent, methodologies and general characteristics of the literature examining the medicinal use of natural honey or its derivatives in different animal species by examining both the veterinary literature and the relevant biomedical literature. The results from this scoping review will provide a foundation for future work that can contribute to evidence-informed decision-making pertaining to the use of honey as a medicine in veterinary practise. Our guiding research question in this scoping review is, “What is the nature and extent of the current body of evidence addressing the medicinal use of natural honey and/or its derivatives in animals?” The following definitions were used:

- Natural honey and/or its derivatives: honey and/or honey-derived products obtained from honeybees of the genus *Apis*.
- Medicinal use: includes treatment of a disease or medical condition, or prevention of a disease or medical condition.
- Animals: livestock species, poultry, horses, companion animals, laboratory animals, exotic species, and wildlife.

## Methods

This review followed the framework for scoping reviews developed by the Joanna Briggs Institute (22). The review was reported using the preferred reporting of items for systematic reviews and meta-analyses extension for scoping reviews (PRISMA-ScR) (23).

### Protocol and Registration

The intended search strategy, eligibility criteria, study selection, data extraction and approach used for charting data were described in the protocol published in advance; this protocol is available online from Systematic Reviews for Animals and Food (SYREAF) and the University of Guelph's institutional repository, at: http://hdl.handle.net/10214/17310 (Appendix A).

### Eligibility Criteria

Studies that investigated natural honey and/or its derivatives as a therapeutic or preventive medical intervention in any live animal species except for insects and humans were eligible. The following types of articles and literature were eligible: theses and dissertations, conference proceedings (>500 words in length), primary research studies and systematic reviews. For primary research studies, eligible study designs were experimental studies (natural and deliberate disease induction), analytical observational studies (cross-sectional, cohort, case-control, other), case reports and case series. Narrative reviews, textbooks, editorials, commentaries, testimonials and letters to the editor were ineligible. Descriptive primary research studies that did not assess an intervention (e.g., prevalence surveys) were not eligible. Studies for which the full text was not available in English or French were excluded. There were no restrictions based on year of publication or study location.

All animal species, both domestic and wild, were considered eligible populations. These species included, but were not limited to, the following groups: livestock, poultry, companion animals (i.e., cats, dogs, horses), exotic species (e.g., birds, reptiles, fish) and wildlife. Laboratory animal species (e.g., mice, rats) used in biomedical research were also eligible. Humans and insects (e.g., honeybees) were ineligible, as it was not within the scope of our review to examine these species.

Eligible interventions were composed, either entirely or in part, of natural honey and/or a derivative of natural honey. We defined a “derivative” of natural honey as any compound that is bee-derived and that is also naturally found in bee-derived honey, for example, lactic acid bacteria. To be eligible, the honey or honey derivative must have been produced by honeybees of the genus *Apis*; products derived from stingless bees (i.e., of the Meliponini tribe) were ineligible. Bee-derived compounds not naturally found in honey were also ineligible; thus, the following compounds were not eligible interventions unless they were combined with natural honey or a honey derivative: propolis, bee pollen, royal jelly, beeswax, bee bread, and bee venom. Artificial honey or related synthetic compounds not produced by *Apis* bees were ineligible. Honey derivatives that were not explicitly obtained from natural honey were ineligible; if the reported source of the derivative was a pharmaceutical or biotechnology company, it was assumed that these derivatives were synthetically produced, and thus, these interventions were deemed ineligible. Studies had to examine an intervention for a clinical condition or injury, either in animal disease or in an animal model of human disease. Thus, studies that examined an eligible intervention as a proof of concept for health improvement in the absence of disease or injury were ineligible. Eligible interventions had to represent either a preventive intervention (designed to prevent the onset of a clinical condition or injury) or a therapeutic intervention (designed to reduce the signs, severity or duration of a clinical condition or injury). Studies investigating the prevention or treatment of toxin exposures were eligible, since these exposures represent a type of injury. Pharmacokinetic studies examining the interaction of honey with other medications and studies using honey as a vehicle for the administration of other substances were ineligible.

Eligible studies were required to have included the measurement of at least one of the following four broad categories of outcomes: clinical, physiological, pathological and mortality. Therefore, mechanistic studies focusing on how honey might work without measuring any relevant outcomes were ineligible. The intervention had to have been examined in live animals; *in vitro* studies were ineligible. Studies reporting the examination of organ physiology *ex vivo* were considered eligible, as long as the intervention was administered to a live animal prior to removal of the organ(s). Outcomes were not restricted to specific body systems.

### Information Sources and Search Strategy

The following electronic databases were searched: MEDLINE® (via PubMed®), Centre for Agricultural Biosciences (CAB) Abstracts (via CAB Direct), AGRICOLA (via ProQuest®), Web of Science Core Collection® and Web of Science SciELO Citation Index®. The following grey literature sources were also searched: Google Scholar® (the first 500 abstracts sorted on relevance) and theses and dissertations (via ProQuest®). The search strategy comprised two concepts: “honey” and “intervention.” The search strategy was adapted for each source, accounting for differences in syntax, indexing and functionality. When applicable, controlled vocabulary (Medical Subject Headings; MeSH) was used. No date or language restrictions were placed on the search. All searches were performed on September 5, 2019. An example of the specific search strategy used to identify relevant articles in CAB Abstracts was as follows:

(((((apitherapy) OR honey) OR ((“lactic acid bacter^*^” AND (honeybee OR “honey bee” OR honey OR Apis OR bee))))) AND (((((((((preventive OR prevent OR prevention OR preventative OR preventable))) OR ((heal OR heals OR healing OR healed OR healer))) OR ((health OR healthy OR healthcare OR healthier OR healthiest))) OR ((intervention OR intervene OR interventions OR intervenes OR intervening))) OR ((treatment OR treatments OR treated OR treating OR treat OR treats))) OR ((medicine OR medicinal OR medicate OR medication OR medicated OR medications OR medicines OR medicating))) OR ((therapeutic OR therapy OR therapeutically OR therapeutics OR therapies OR therapeutical)))) AND (((sc:ft)))

All searches performed and their results are presented in Appendix B. Search results were uploaded into EndNote (Clarivate Analytics, Philadelphia, Pennsylvania, US), and duplicates were removed. Search results were then uploaded to an online systematic review program (Distiller SR®, Ottawa, ON, Canada), and additional duplicates were removed. To identify additional relevant articles that may not have been identified by our electronic searches, reference list checking was performed for 50 of the most recent articles included in the review.

### Selection of Sources of Evidence

All reviewers received training prior to screening and data extraction to ensure consistency. Articles underwent two levels of screening and one level of data extraction. Screening and data extraction were performed independently by two reviewers using forms pretested on a subset of records (50 in the first level, 50 in the second level, 15 in the third level). At all stages of screening and data extraction, conflicts were resolved by consensus. Level 1 screening was performed on titles and abstracts using two questions:

1. “Does the title/abstract describe primary research or a systematic review?”
2. “Does the title/abstract describe an investigation of the use of natural honey and/or its derivatives as an intervention in a live animal? (Studies assessing mortality are eligible)”

If both reviewers answered “no” to at least one question, the reference was excluded. References moved forward to level 2 for full-text screening if the answer for both questions was “yes” or “unclear.” Level 2 screening was performed on full-text articles using the following questions, which included iterations of level 1 questions to ensure relevance:

3. “Is the article in **English** or **French**?”
4. “Is the full-text publication **>500 words**?”
5. “Does the full-text publication describe a **primary study** or a **systematic review**?”
6. “Was the intervention performed in **LIVE animals**? (Exclude insects and humans)”
7. “Is the intervention a natural honey and/or a honey derivative **produced by honeybees (genus** ***Apis*)**?”
8. “Is natural honey and/or its derivatives being investigated as a **therapeutic** or **preventive** intervention in animals?”

The order of level 2 screening questions was modified from the protocol to capture a meaningful hierarchy of the reasons for article exclusion, and one question was removed, since it was deemed not relevant to this stage. In addition, the emphasis (bolding of the text) of several level 2 screening questions was modified slightly from the protocol to clarify ambiguities and emphasise certain concepts, allowing for ease of interpretation. Forms and guidelines used to perform screening are available in Appendix C.

### Data Charting Process and Data Items

The following study characteristics were extracted: year of publication, country and study design. If the location of the study was not reported, the country of the first author's affiliation was recorded. The study design extracted was based on the design used in the study, not the design reported by authors (if inconsistent), and the following categories were used: case report/case series, observational studies evaluating an intervention, challenge trials, controlled trials and systematic reviews. A challenge trial was defined as a controlled experiment with deliberate disease induction, and a controlled trial was defined as a controlled experiment with natural disease exposure.

The animal species studied was extracted, along with whether or not the study represented biomedical research (i.e., humans as the target population) or veterinary research (i.e., animals as the target population). Studies that did not explicitly state that animals were used as a model for human disease were assumed to represent biomedical research if the article extensively referenced human research and/or did not explicitly mention veterinary applications.

Details of each unique intervention within a study were recorded, including the type of honey and/or honey derivative, and whether the intervention was given alone or combined with another substance or intervention. The type of honey was extracted based on the region of origin or the main floral source (e.g., gelam honey, tualang honey, manuka honey). The status of honey interventions was recorded as either “medical” or “non-medical”; these categories were modified from “medicinal” and “non-medicinal” in the protocol to avoid misrepresentation, since all types of honey have the potential to possess medicinal properties. In order for a honey to be considered medical, the study either had to report that a commercially available medical-grade honey was used or had to list the product manufacturer (indicating that third-party testing and/or product standardisation was performed). For honey or honey derivative interventions that included additional components, these additional components were classified under the following four categories: “herbal products,” “drugs,” “other bee products” and “other.” If applicable, the type of “other bee products” was extracted (e.g., propolis, bee pollen). The intervention was classified as either a preventive or a therapeutic intervention; the intervention was considered preventive if disease or injury had not yet occurred at the time of administration of the intervention, whereas therapeutic interventions were those administered after the disease/injury had occurred. Cases where the intervention was arguably considered both a preventive and a therapeutic were classified within what was judged to be the most appropriate category; for instance, interventions administered simultaneously with a toxin were classified as preventive interventions. The mode of administration of the intervention was also recorded; for ease of data extraction, only unique modes of administration were extracted for a given study. For instance, a study examining three unique interventions, with two interventions given orally and one intervention given intravenously, was extracted as one count each of “oral” and “intravenous.”

The disease or injury targeted by the intervention was extracted and classified within one of the following body systems: “gastrointestinal,” “musculoskeletal,” “nervous system,” “cardiovascular,” “lymphatic/immune system,” “endocrine/metabolic,” “urinary/renal,” “reproductive,” “dermatological” and “other.” If applicable, more than one body system and disease or injury was recorded for an individual study. Conditions affecting multiple body systems were classified based on physiology rather than anatomy. For instance, polycystic ovarian syndrome was classified as “endocrine” rather than “reproductive.” If the impact of toxicity was evaluated predominantly in one or two body systems, these were extracted separately; studies that evaluated the impact of toxicity on more than two body systems were classified under “other.” Ocular conditions, neoplasia, arthritis, haematological conditions and respiratory conditions were also classified as “other.” Burns were considered separately from other wounds, extracted as “burns” and “wound healing,” respectively.

The type of outcome(s) measured was extracted under one of the following categories: “clinical,” “physiological,” “pathological,” “mortality.” To ensure consistency and ease of data extraction, the protocol was modified slightly, in that the method of outcome measurement determined its classification. Outcomes that were observable in live animals were extracted as clinical outcomes and included, but were not limited to, pain scores, behaviour scores and time to healing (for wounds). Physiological outcomes represented outcomes that were not directly observable in live animals and that required the use of specialised equipment or tests. Examples of physiological outcomes included heart rate, body temperature, blood glucose (assessed via a glucometer) and electrical activity of the heart (assessed via electrocardiography). Outcomes that involved the removal of samples from the animal (e.g., biopsies, blood samples) or assessed via post-mortem examination were classified as pathological outcomes (referred to as “pathological lesions” in our protocol). Thus, pathological outcomes could have been assessed in either live animals or euthanized/deceased animals. All forms and guidelines used for data extraction are available in Appendix D.

For publications reporting multiple studies (each study with its own unique control group), each study was extracted separately. Studies evaluating multiple intervention groups against a single control group were extracted as a single study. For ease of data extraction, different doses of the same intervention were collapsed and extracted as a single intervention.

### Synthesis of Results

A figure was generated to present the flow of articles through the review process, including the number of articles excluded at each level and reasons for exclusion. Study characteristics, study design, the types of interventions used and outcomes assessed were summarised descriptively in the text and in the tables. Figures were also used to convey this information, where appropriate. Separate tables were generated for veterinary and biomedical research for each type of study design. Data were presented at the publication level if they were collapsible at this level, as they were for country of study and year of publication. Otherwise, data were presented at the study level (i.e., study design, animal species, indication, outcomes assessed). In some cases, data were collapsed at the study level, if applicable (i.e., mode of administration, intervention type). Study designs for which there were few publications (<5) were summarised narratively in the text.

## Results

### Selection of Sources of Evidence

**Figure 1:**
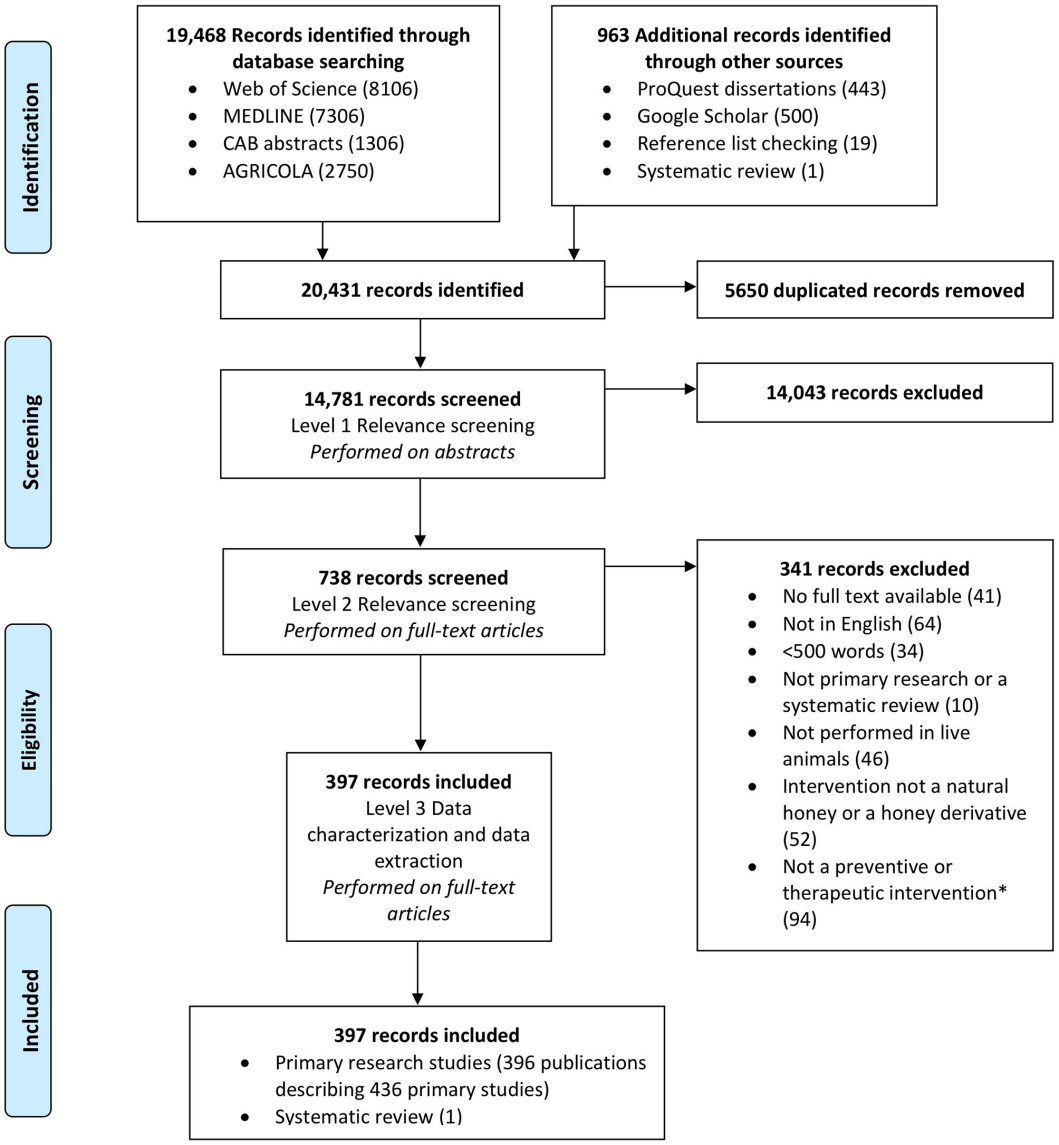
PRISMA (preferred reporting items for systematic reviews and meta-analyses) flowchart showing the selection of studies eligible for a scoping review of the evidence for the medicinal use of natural honey in animals.

Figure 1 depicts the flow of articles, exclusions and the reasons for exclusion. A total of 14,781 unique references were identified. Of these, 738 articles were identified as potentially relevant after level 1 screening; 341 of these records were excluded at level 2. A total of 64 records were excluded at level 2 as the full text was not in English or French; most of these articles were in Spanish (*n* = 23), with a smaller number in Russian (*n* = 14), German (*n* = 8) and Chinese (*n* = 6), among others. In total, 397 articles were included in this review (Appendix E).

### Characteristics of Sources of Evidence

One systematic review was identified; all other records were primary literature. The majority of the included publications represented biomedical research (*n* = 350), with a smaller number of veterinary research articles (*n* = 47; Table 1). One veterinary publication reported two studies, and a number of biomedical publications reported more than one study (*n* = 98/350). Thus, a total of 436 primary research studies from 397 publications were identified and are presented herein. Apart from the systematic review, all biomedical research studies were challenge trials (Table 1). Among veterinary research studies, most were case series or case reports (*n* = 23) or challenge trials (*n* = 18), with a few controlled trials (*n* = 8; Table 1). No observational studies or scoping reviews were identified by this review.

**Table 1:** Study design^a^ of 397 publications included in a scoping review of the evidence for the medicinal use of honey in animals.

**Study design**	**Veterinary research**	**Biomedical research**
Case report/case series	23	0
Controlled trials	8	0
Challenge trials	18	349 (387 studies)
Systematic review	0	1
Total no. publications (no. studies^b^)	47 (49 studies)	350 (388 studies)

a Study design was determined based on methods used, not as reported. No observational studies or scoping reviews were identified.

b Total number of studies is greater than the total number of publications since some publications reported more than one study.

**Figure 2:**
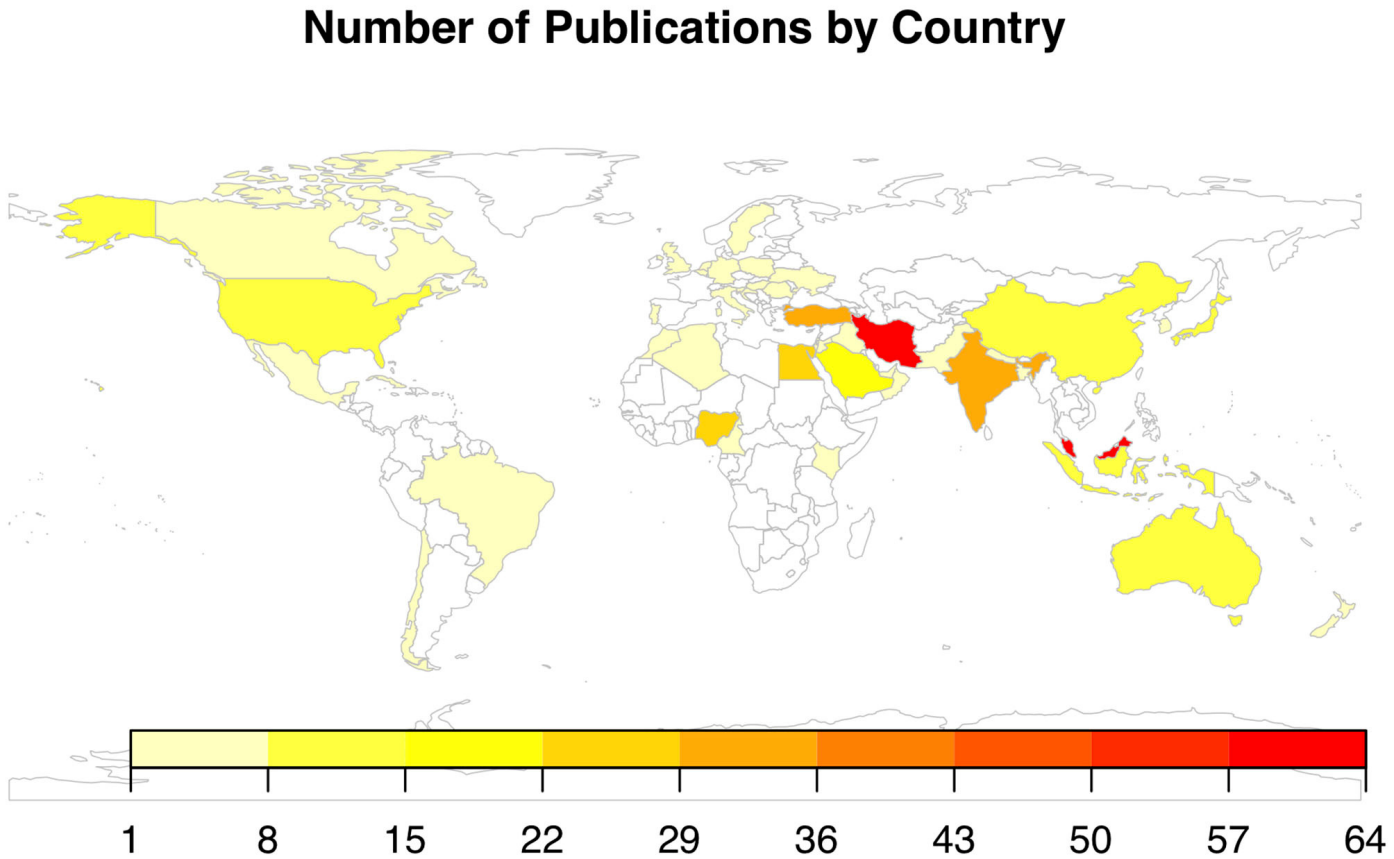
Chloropleth map of the number of publications by country for articles included in a scoping review of the evidence for the medicinal use of honey in animals (*n* = 397), with 45 countries represented. Iran and Malaysia contributed the greatest number of publications.

**Figure 3:**
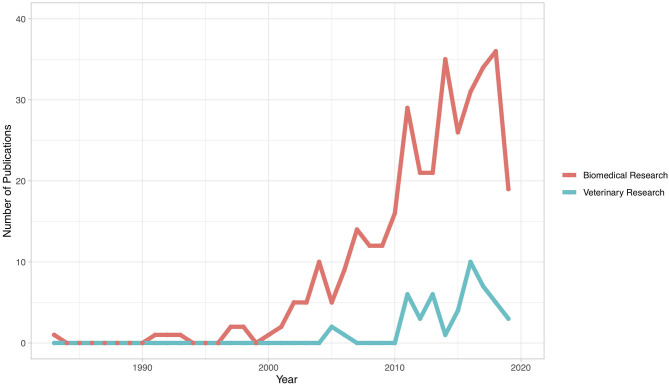
Year of publication for publications included in a scoping review of the evidence for the medicinal use of natural honey in animals (*n* = 397). Shaded region represents an incomplete picture of the total number of publications in 2019, since the search was performed in September 2019 and thus did not capture all studies published in that year.

Figure 2 illustrates the number of publications by country of study, with 45 countries represented. The greatest number of publications was from Malaysia (*n* = 64), followed by Iran (*n* = 61), Turkey (*n* = 34), India (*n* = 31), Egypt (*n* = 27), Nigeria (*n* = 24), Saudi Arabia (*n* = 19), China (*n* = 12), Japan (*n* = 10), and Indonesia (*n* = 10). All other countries had fewer than 10 publications. The distribution of veterinary publications was as follows: Australia (*n* = 6), Nigeria (*n* = 6), Egypt (*n* = 5), India (*n* = 4), Brazil (*n* = 3), United States (*n* = 3), Algeria (*n* = 2), Germany (*n* = 2), Iran (*n* = 2), and Jordan (*n* = 2). The remaining veterinary publications were from countries with only one publication each. Figure 3 illustrates the number of articles for both veterinary and biomedical research by year of publication. The earliest publication identified by this review was published in 1983, and a few publications were identified immediately thereafter. The earliest veterinary publications identified were two studies published in 2005. The number of biomedical research publications began to steadily increase in 2000, and a similar, but smaller, increase in veterinary publications began more recently in 2010 (Figure 3).

The indications for use, categories of outcomes assessed and intervention type by animal species for each type of study design are provided in Tables 2–4 for veterinary research and Table 5 for biomedical research. A variety of animal species were investigated in veterinary studies, most commonly dogs (*n* = 12/49), cats (*n* = 5/49), horses (*n* = 12/49) and cattle (*n* = 7/49); other species less commonly examined included donkeys (*n* = 5/49), chickens (*n* = 3/49), buffalo (*n* = 1/49), pigs (*n* = 1/49), goats (*n* = 1/49), quail (*n* = 1/49), and fish (*n* = 2/49). In addition, there were two veterinary case reports describing a macaque (*n* = 1/49) and a tortoise (*n* = 1/49). The vast majority of biomedical studies examined rats (*n* = 271/387), mice (*n* = 82/387) and other rodents (*n* = 3/387), although several studies used rabbits (*n* = 23/387), dogs (*n* = 6/387), and livestock (*n* = 7/387; Table 5) as animal models. Several studies examined more than one animal species; thus, the total number of species examined exceeds the total number of studies. In both veterinary and biomedical studies, most studies examined clinical outcomes or pathological outcomes; physiological outcomes and mortality were less commonly assessed.

**Table 2:** Summary of 23 veterinary case reports/series included in a scoping review of the evidence for the medicinal use of honey in animals, characterised by species, body system, indication for use, outcome category and type of honey used.

	**Species studied**						**Total^**e**^**
	**Canine**	**Feline**	**Equine**	**Bovine**	**Porcine**	**Exotic^**d**^**	
**BODY SYSTEM**^**a**^**: INDICATION FOR USE**							
Musculoskeletal: fracture	1	1	0	0	0	0	2
Dermatological: burns	2	0	1	2	0	0	5
Dermatological: wound healing^a^	5	2	4	3	0	2	16
Other dermatological^b^	1	0	0	0	1	0	2
**OUTCOME CATEGORIES**							
Clinical outcomes	7	3	5	5	1	2	23
Physiological outcomes	0	2	0	1	0	1	4
Pathological outcomes	1	0	0	0	0	0	1
**TYPE OF INTERVENTION USED**							
Medical honey	3	1	0	0	0	1	5
Non-medical honey	4	2	5	5	1	1	18
Honey derivative	0	0	1^c^	0	0	0	1

a In addition to general wound healing, other specific wounds included an eye enucleation wound (n = 1), lesions from foot and mouth disease (n = 2), necrotizing fasciitis (n = 1), and gangrenous mastitis (n = 1).

b “Other dermatological” included otitis externa (canine) and epitheliogenesis imperfecta (porcine).

c Lactic acid bacteria was the honey derivative used in this study.

d Exotic species included a macaque and a tortoise.

e Total number exceeded the total number of studies, since some studies studied multiple interventions, measured multiple categories of outcomes and/or used the intervention for more than one indication.

**Table 3:** Summary of eight veterinary controlled trials included in a scoping review of the evidence for the medicinal use of honey in animals, characterised by species, indication for use, outcome category and type of honey used.

	**Species studied**					**Total^**b**^**
	**Canine**	**Feline**	**Equine**	**Bovine**	**Avian**	
**BODY SYSTEM: INDICATION FOR USE**						
Cardiovascular: heat stress	0	0	0	0	1	1
Reproductive: subclinical mastitis	0	0	0	1	0	1
Dermatological: wound healing	0	1	2	0	0	3
Other dermatological^a^	1	0	0	1	0	2
Other: hatchability	0	0	0	0	1	1
**OUTCOME CATEGORIES**						
Clinical outcomes	1	1	2	2	0	6
Physiological outcomes	0	0	0	0	1	1
Pathological outcomes	1	0	1	1	0	3
Mortality	0	0	0	0	1	1
**TYPE OF INTERVENTION USED**						
Medical honey	1	1	1	0	0	3
Non-medical honey	0	0	1	2	2	5

a “Other dermatological” included digital dermatitis (bovine) and bacterial colonisation at an intravenous catheter site (canine).

b Total number exceeded the total number of studies, since some studies studied multiple interventions, measured multiple categories of outcomes and/or used the intervention for more than one indication.

**Table 4:** Summary of 18 veterinary challenge trials included in a scoping review of the evidence for the medicinal use of honey in animals, characterised by species, indication for use, outcome category and type of honey used.

	**Species studied**						**Total^**d**^**
	**Canine**	**Feline**	**Equine**	**Avian**	**Caprine**	**Fish**	
**BODY SYSTEM: INDICATION FOR USE**							
Gastrointestinal^a^	1	0	0	1	0	0	2
Musculoskeletal: bone graft	1	0	0	0	0	0	1
Lymphatic/immune system^b^	0	0	0	1	0	1	2
Dermatological: wound healing	3	0	8^c^	0	1	0	12
Other: tebuconazole toxicity	0	0	0	0	0	1	1
**OUTCOME CATEGORIES**							
Clinical outcomes	3	1	8	1	0	1	14
Physiological outcomes	0	1	0	1	0	1	3
Pathological outcomes	3	1	5	2	1	1	13
Mortality	0	0	0	0	0	1	1
**TYPE OF INTERVENTION USED**							
Medical honey	0	0	4	0	0	0	4
Non-medical honey	4	1	5	2	1	2	15

a Indications for use were post-operative peritoneal adhesions (canine) and aflatoxin ingestion (avian).

b Indications for use were Aeromonas hydrophila infection (fish) and infection with Newcastle Disease Virus and Avian Influenza (avian).

c This total included five studies in horses and three studies in donkeys.

d Total number exceeded the total number of studies, since some studies studied multiple interventions, measured multiple categories of outcomes and/or used the intervention for more than one indication.

**Table 5:** Summary of 387 biomedical challenge trials included in a scoping review of the evidence for the medicinal use of honey in animals, characterised by species, indication for use, outcome category and type of honey used.

	**Species studied**							**Total studies^**d**^**
	**Rat**	**Mouse**	**Other rodents^**c**^**	**Rabbit**	**Canine**	**Pigs**	**Sheep**	
**BODY SYSTEM: INDICATION FOR USE**^**a**^								
Gastrointestinal	74	14	0	2	3	0	1	94
Musculoskeletal	8	0	0	0	0	0	0	8
Nervous system	19	7	1	0	0	0	0	27
Cardiovascular	15	1	0	1	0	0	0	17
Lymphatic/immune system	10	8	0	0	0	0	1	19
Endocrine/metabolic	24	0	0	0	0	0	0	24
Urinary/renal	23	2	0	1	0	0	0	26
Reproductive	19	0	0	0	0	0	0	19
Dermatological: burns	21	4	2	4	2	4	0	37
Dermatological: wound healing	37	23	0	9	0	0	0	69
Other dermatological	4	3	0	0	0	1	0	8
Other	40	21	0	8	1	0	0	70
**OUTCOME CATEGORIES**								
Clinical outcomes	135	49	1	16	4	5	1	211
Physiological outcomes	26	5	0	3	0	0	0	34
Pathological outcomes	239	68	3	19	5	4	2	340
Mortality	8	7	0	2	0	0	0	17
**TYPE OF INTERVENTION USED**								
Medical honey	9	4	0	2	0	1	0	16
Non-medical honey	261	77	3	21	6	4	2	374
Honey derivative^b^	3	4	0	0	0	0	1	8

a Details for indications for use by body system are available in Appendix F.

b Honey derivatives included honey ethyl acetate extract, honey methanolic extract and eugenol.

c “Other rodents” included gerbils and guinea pigs.

d Total number exceeded the total number of studies, since some studies studied multiple species, multiple interventions, measured multiple categories of outcomes and/or used the intervention for more than one indication.

Among all types of veterinary studies, wound healing was the most common indication studied (*n* = 31/49). Veterinary case reports/series reported a variety of wound healing applications, including a post-enucleation wound, a contaminated open fracture, feline gangrenous mastitis, lesions from foot-and-mouth disease in livestock and canine necrotizing fasciitis. Among veterinary controlled trials, examples of indications included the treatment of bovine subclinical mastitis, the treatment of equine lower extremity wounds and the treatment of bovine digital dermatitis. Examples of indications (aside from wound healing) among veterinary challenge trials included the following: the prevention of post-operative peritoneal adhesions in dogs, the treatment of aflatoxin ingestion in poultry, the treatment of *Aeromonas hydrophila* infection in fish, the prevention of tebuconazole toxicity in fish and the preservation of bone allografts for use in the surgical repair of cortical bone defects in cats.

Among biomedical challenge trials, a wide variety of indications were examined, and, in contrast with veterinary studies, all body systems were represented (Table 5). The indications most commonly studied were gastrointestinal conditions (*n* = 93/387), burns (*n* = 37/387) and healing of wounds other than burns (*n* = 69/387; Table 5). Examples of commonly studied gastrointestinal conditions included the treatment or prevention of gastric ulcers (*n* = 22/93) and the prevention of post-operative peritoneal adhesions (*n* = 9/93). Prevention of hepatic and/or renal toxicities caused by fungi, heavy metal exposures and pharmaceuticals (e.g., non-steroidal anti-inflammatory drugs) were also commonly studied in biomedical research (*n* = 42/387). A variety of indications related to the nervous system were studied: the prevention of toxin-induced seizures (*n* = 2/387), the treatment of pain (*n* = 8/387) and the improvement of cognitive function (often memory) following toxin exposure or cerebrovascular accidents (*n* = 9/387). Cardiovascular indications studied included the prevention of myocardial infarctions (*n* = 3/387), the prevention of cardiac arrhythmias (*n* = 1/387), the treatment of congestive heart failure (*n* = 1/387), the prevention of atherosclerosis (*n* = 1/387) and the treatment of hypertension (*n* = 3/387). Under endocrine/metabolic conditions, the modulation of glycaemic control in diabetes was commonly studied (*n* = 15/387), and additional indications included the prevention of menopausal syndrome (*n* = 2/387) and the prevention or treatment of polycystic ovarian syndrome (*n* = 3/387). Lymphatic/immune indications included the treatment or prevention of bacterial and parasitic infections (e.g., *Salmonella*, typhoid, trypanosomes, schistosomes), as well as fungal infections (*n* = 16/387). Autoimmune indications such as rheumatoid arthritis were also studied (*n* = 2/387). In addition to the treatment of renal injury caused by ischaemia or toxin exposure, urethral conditions were examined (e.g., treatment of urethral strictures; *n* = 2/387). Among indications classified under “other” body systems, tumour growth and metastasis were commonly studied (*n* = 26/387). Ocular conditions studied included dry eye (*n* = 1/387), corneal injury (*n* = 3/387), keratitis (*n* = 1/387), bacterial conjunctivitis (*n* = 1/387) and allergic conditions such as asthma (*n* = 4/387). A comprehensive list of indications among biomedical challenge trials is available in Appendix F. Most studies investigated the intervention(s) for the treatment of a disease or injury (*n* = 257/436), whereas fewer studies examined honey and/or honey derivatives for the prevention of disease or injury (*n* = 179/436).

The majority of studies examined non-medical honey as an intervention (*n* = 412/436). Fewer studies examined medical honey (*n* = 28/436), and only nine studies examined a derivative from natural honey. The majority of studies examined only one honey intervention against a control group (*n* = 338/436). For the subset of veterinary studies, most examined a single intervention (*n* = 41/49), the majority of which was non-medical honey (*n* = 31/41); the remaining veterinary studies evaluated medical honey (*n* = 10/41). Of the remaining studies that involved multiple honey interventions (*n* = 98/436), most were biomedical challenge trials (*n* = 90/98) with fewer veterinary studies (*n* = 8/98). Studies comparing multiple honey interventions examined between two and six interventions (mean 2.4); most of these studies included a comparison between at least two different non-medical honeys (*n* = 82), seven studies included a comparison between different medical honeys and four studies performed head-to-head comparisons between medical and non-medical honeys. Of the eight veterinary studies that examined multiple honey interventions, only two studies examined medical honeys; one study compared different medical honeys, and the other performed a head-to-head comparison between medical and non-medical honeys.

About half of the studies did not specify the floral source of non-medical honey used (*n* = 221/412). Among the studies that examined non-medical honey, the most commonly used honeys were tualang honey (*n* = 46/191), acacia honey (*n* = 18/191), gelam honey (*n* = 17/191), sidr honey (*n* = 10/191), and chestnut honey (*n* = 6/191). Many studies that reported manuka honey did not specify the honey as medical-grade or did not specify a manufacturer; thus, these were classified as non-medical manuka honeys (*n* = 22/191). Among studies that examined medical honey, manuka honey was the most common (*n* = 20/28), with Medihoney® (*n* = 8/28) as the most commonly used brand. Several studies used L-Mesitran® (*n* = 4/28), a commercially available multi-floral medical-grade honey.

Most studies included interventions administered as honey alone (*n* = 327/436), as opposed to combining honey with at least one other substance (*n* = 160/436; some studies examined several interventions). Sixteen studies combined honey interventions with another bee product: propolis (*n* = 14), bee pollen (*n* = 6), royal jelly (*n* = 6), drone larvae (*n* = 1), bee venom (*n* = 1), apilarnil (*n* = 1), and proapilarnil (*n* = 1). The remaining studies that combined interventions with another substance administered some combination of herbal products, drugs or other substances. Commonly studied herbal products included ginger, garlic, turmeric, aloe vera, *Semecarpus anacardium, Emblica officinalis* and traditional multi-herbal medicines such as Kalpaamruthaa and Kyung-Ok-Ko. In biomedical wound healing studies, interventions frequently included the topical application of hydrogels infused with compounds such as pectin or chitosan.

**Figure 4:**
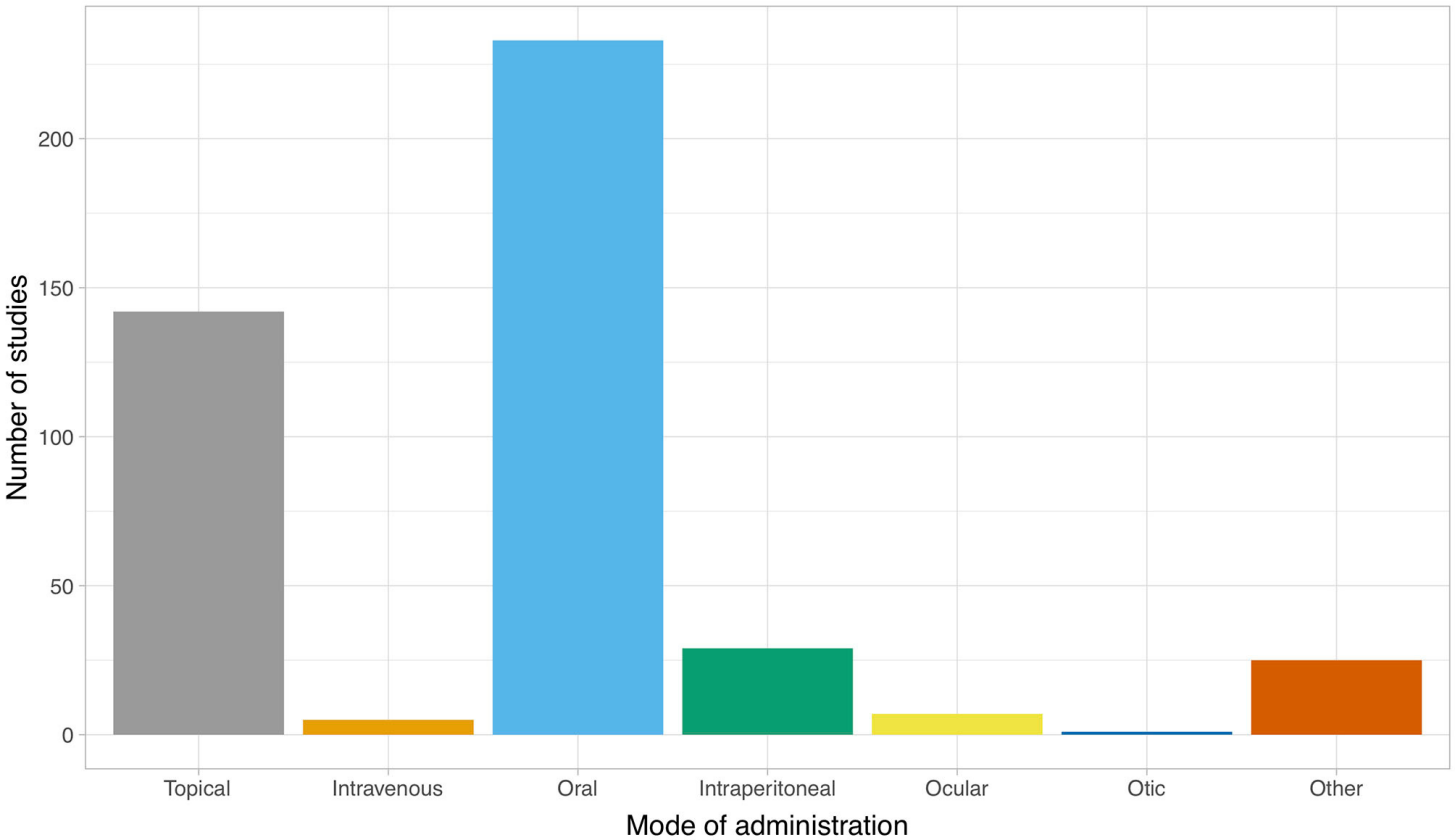
Mode of administration of the intervention for publications (*n* = 397) included in a scoping review of the evidence for the medicinal use of natural honey in animals. Data are presented at the study level. The total count exceeds 397 since some publications reported several studies, and some studies reported more than one mode of administration.

Figure 4 illustrates the mode of administration of the intervention(s) at the study level for both veterinary and biomedical studies. The majority of studies administered the intervention orally (*n* = 233/436), topically (*n* = 141/436) or intraperitoneally (*n* = 29/436). Additional routes of administration included intravenous, ocular, otic or “other,” which included via inhalation, sinus irrigation, intramammary, intraurethrally, via an enema, topically on the oral mucosa, on a surgical implant or in water (i.e., for fish). The distribution of the mode of administration among the subset of veterinary studies differed; the majority of veterinary studies administered the intervention topically (*n* = 38/49). Oral administration was less common (*n* = 4/49), and other routes of administration were less commonly used (*n* ≤ 2; oral, in ovo, in water, intramammary, otic, intraperitoneal, preservation of bone allografts).

The systematic review identified by our scoping study was a biomedical research study from Malaysia, published in 2018. The aim was to “evaluate the therapeutic potential of honey in the context of its gastroprotective function against (non-steroidal anti-inflammatory drug)-induced gastric ulcers” in rats (24). Non-rat animal models were ineligible for this review, and the comparator group was required to be exposed to non-steroidal anti-inflammatory drugs. No meta-analysis was performed.

## Discussion

### Summary of Evidence

Our review identified an increase in the volume of scientific research examining the medicinal value of honey in animals beginning in the year 2000. Veterinary publications, however, were only first identified in 2005 and have been on the rise more recently since 2010. Nearly a third of all publications originated from only two countries: Malaysia and Iran. This stands in contrast to western nations such as the United States, Australia and a number of European nations, each of which had fewer than 10 relevant publications. The overwhelming majority (>85%) of publications were biomedical research studies performed in mice or rats, with humans as the target population. We identified little relevant veterinary research, and roughly half of this work was in the form of case reports or case series, which provide a low quality of scientific evidence for evaluating efficacy due to the absence of a control group for comparison (25). Based on the findings of our review, there is currently insufficient veterinary literature for synthesis research methods to assess the efficacy of honey for any medical condition in livestock or in companion animals. Only one veterinary area was identified as possibly having potential for synthesis research, namely, equine wound healing, as eight challenge trials are available (three in donkeys, five in horses). In contrast with the veterinary literature, a number of topic areas in the biomedical literature appear to contain sufficient primary research for synthesis work. Indeed, the sole systematic review identified by our review evaluated a topic in the biomedical literature. The following areas of the biomedical literature examining rodents would likely contain sufficient primary work for synthesis research: the efficacy of honey in wound healing or burns, glycaemic control in diabetes and tumour growth and/or metastasis. However, given the substantial body of evidence examining the efficacy of honey for wound healing in humans (3, 12, 13, 16), additional systematic reviews examining this particular topic using biomedical animal models for human outcomes may not be informative or necessary. Rather, the biomedical literature may prove most useful for hypothesis generation for researchers evaluating proof of concept for novel clinical applications of honey in both veterinary and human fields.

### Research Gaps: Primary Research Needs for Future Synthesis Work

Our review findings have demonstrated a clear need for additional primary research examining honey in a veterinary context. The majority of veterinary research thus far has been on wound healing, though a small number of other non-dermatological applications have also been explored. Given the advanced state of the human and biomedical literature on honey and wound healing (5), future veterinary research in this area is likely to be geared towards optimising treatment regimens in order to achieve reliable clinical outcomes in animals. With limited primary work in this area, however, additional research is needed concerned solely with the efficacy of honey as a wound-healing agent in companion animals and livestock. A species-specific approach will also be essential for future primary veterinary research to appropriately assess efficacy, safety and, if applicable, the optimal type of honey, dose and route of administration in each instance. The need for a species-specific approach is clear, given examples such as wound healing in equines, in which the production of exuberant granulation tissue in wounds can impede healing and hinder a return to optimal function (26). Roughly a third of the veterinary literature on honey as a wound-healing agent consists of studies examining wound healing in equines. As most of these studies were challenge trials, high-quality controlled trials would help advance this field and provide valuable input for future systematic reviews.

Further primary research is needed to expand the breadth and depth of the limited preliminary veterinary research examining indications other than wound healing. Indications other than wound healing that have figured in the veterinary literature thus far include subclinical mastitis, fracture healing and dermatological lesions caused by bacteria and viruses (bovine digital dermatitis, foot-and-mouth disease). The extensive body of biomedical research examining a diversity of indications in animal models may be used to guide future preliminary veterinary research. Unexplored indications suggested by the biomedical literature include the (primary or adjunct) treatment of pain, parasitic infections, gastrointestinal ailments, toxicities, ocular conditions, neoplasia and metabolic disorders.

The main obstacle to synthesis work for veterinary applications is the limited primary veterinary research suitable to this end. An additional potential obstacle to future synthesis work in this field, however, lies in the heterogeneity of the medical intervention itself. Our review identified heterogeneity in honey as a medical intervention relating to differences in floral source and the method(s) of processing (e.g., irradiation, filtration, and pasteurisation). In the human literature, several systematic review and meta-analyses have collapsed honeys from different floral sources and those processed using different methods in their meta-analyses (3, 12, 13, 16). Since few meta-analyses have performed subgroup analyses by honey type, the importance of honey type is currently unknown, though at least one such work suggests that differences in honey types can significantly impact treatment outcomes (17). To ensure comparability between different studies and collapsibility for future meta-analyses, we suggest that future primary veterinary research focus on standardised medical-grade honey and/or perform head-to-head comparisons between medical-grade and non-medical-grade honey against a common control group to address important research gaps regarding the heterogeneity of honey as a treatment.

### Limitations

One of the main limitations of our review is that the quality of the literature was not assessed, and although this is not considered a typical approach for a scoping review (27, 28), it is an important consideration for the interpretation of the data presented herein. Some additional limitations of this review are related to challenges associated with the identification of potentially relevant articles. A number of relevant articles identified through reference list checking were not identified through our electronic searches, as the journals in which they appeared were not indexed in the databases we searched. Due to the specific language of the search strategy, articles that examined certain honey derivatives that were not listed as search terms may not have been identified. Furthermore, studies were excluded at level 2 if they used honey derivatives and had an associated pharmaceutical manufacturer listed; these were assumed to be synthetic, and it was not within the scope of this review to verify this assumption. By restricting this review to natural honey and derivatives of natural honey, we opted to maintain consistency and to avoid conflating synthetic products with products produced by honeybees. Therefore, although the total number of studies examining honey derivatives was small, it is possible that some relevant studies were missed. In addition, the exclusion of many studies based on language may have impacted our findings; future scoping reviews should consider including resources for translation of literature.

To simplify data extraction, intervention level data were collapsed for certain metrics and presented at the study level (such as with the mode of administration and intervention type); thus, true intervention level data are not available from this review. Nonetheless, the collapsed data presented here provide an overview of this aspect of the existing literature and also provide valuable insight for the design of future primary research studies and potential pitfalls of future synthesis work. A similar simplifying approach was used to classify the type of honey, as either medical or non-medical; this simplification does not capture the nuances of different types of honey, which may be related to the method of processing (i.e., raw vs. unpasteurized). Finally, we collapsed slightly different indications in order to facilitate data extraction and interpretation, but for the purposes of a systematic review, it may be important to consider these indications separately (e.g., healing of non-infected wounds was considered analogous to healing of wounds deliberately inoculated with multi-drug resistant bacteria).

### Conclusions

Currently, there is limited veterinary research examining the efficacy of natural honey and its derivatives in animals, and there is insufficient literature for synthesis research in companion animals (dogs and cats) and livestock species. There may be sufficient literature to assess the efficacy of honey for wound healing in equine species, with eight veterinary challenge trials identified. The vast majority of research examining honey in animals consisted of biomedical challenge trials performed in rats and mice, which assessed a wide range of medical ailments including wound healing, neoplasia, ocular conditions, bacterial and parasitic infections, gastrointestinal ailments and endocrine conditions. The diversity of indications and outcomes assessed, along with the heterogeneity of honey treatments, presents a challenge for future systematic reviews and meta-analyses. In particular, the heterogeneity of honeys attributable to floral source(s) and the distinctions between raw, processed and irradiated honey are important considerations for the collapsibility of different interventions for future meta-analyses. With much of the veterinary literature having a low evidentiary value (i.e., case reports or case series), high-quality primary veterinary research in the form of controlled trials is needed to advance this field and to provide sound data for synthesis work that will form the basis for evidence-based assessments of the efficacy of honey for various indications in companion and livestock species.

## Data Availability Statement

The original contributions presented in the study are included in the article/Supplementary Materials, further inquiries can be directed to the corresponding author/s.

## Author Contributions

NV developed protocols, forms and the guidelines of the review, with input from JS and EV. Screening was performed by EV, JS, and NV. Data extraction was performed by EV, AN, and NV. NV performed data analysis and drafted the manuscript, with input from EV and JS. All authors contributed to the article and approved the submitted version.

## Conflict of Interest

The authors declare that the research was conducted in the absence of any commercial or financial relationships that could be construed as a potential conflict of interest.

## Supplementary Material

Table 1 Raw data.

Data Sheet 1 Appendix A.

Data Sheet 2 Appendix B.

Data Sheet 3 Appendix C.

Data Sheet 4 Appendix D.

Data Sheet 5 Appendix E.

Data Sheet 6 Appendix F.
