# Supplementary material for: A Scoping Review of the Evidence for the Medicinal Use of Natural Honey in Animals
Source: Front Vet Sci. 2021 Jan 18;7:618301. doi: 10.3389/fvets.2020.618301 (PMC7847899; doi:10.3389/fvets.2020.618301)
Supplement: Data Sheet 1 — Appendix A. [file Data_Sheet_1.PDF]

Jan Sargeant
Digitally signed by Jan Sargeant
DN: cn=Jan Sargeant, o=University of Guelph, ou=Population Medicine, email=sargeanj@uoguelph.ca, c=CA
Date: 2019.09.03 08:29:45 -04'00'

A Protocol for a Scoping Review of the Evidence for the Medicinal Use of Natural Honey and/or its Derivatives in Animals

Author details
Nadine A. Vogt[1], Jan M. Sargeant[1,2], Danielle A. Julien[1]

[1]Department of Population Medicine, Ontario Veterinary College, University of Guelph, Guelph Ontario, Canada
[2]Centre for Public Health and Zoonoses, University of Guelph, Guelph, Ontario, Canada

Corresponding author:
Nadine A. Vogt[1]
Email: nvogt@uoguelph.ca

Introduction

Rationale

Honey is a viscous liquid solution produced by honeybees (genus *Apis*) from the nectar of flowers or the sap of plants[1]. The use of honey as a healing agent in folk medicine predates written records[1]. More recently, the therapeutic value of honey in modern human medicine has emerged as a topic of interest, especially in light of the increasing prevalence of antimicrobial resistance globally[2]. There is evidence in the scientific literature supporting the use of honey as an antimicrobial, anti-inflammatory, and anti-tumour agent[3-6]. Honey has also been critically examined for its potential in humans as a therapeutic agent in burns and wound healing, as a cardiovascular potentiating agent, and for regulation of glycemic response[1]. Several systematic reviews and meta-analyses evaluating the efficacy of honey for various therapeutic purposes are available in the human literature[7-9]. In contrast, there is little information available in the veterinary literature regarding the extent of scientific investigation of honey for different therapeutic uses across different animal species[10-12], which would serve as a basis for research synthesis methods such as systematic reviews and meta-analyses.

This protocol describes the methods for conducting a scoping review which will examine the extent, methodologies, and general characteristics of the literature examining the medicinal use of natural honey and/or its derivatives in animals. To the best of our knowledge, and at the time of writing, no scoping reviews or systematic reviews or meta-analyses on this topic have been performed.

Research Question and Objectives

In this scoping review, our broad research question is: "*What is the nature and extent of the current body of evidence addressing the medicinal use of natural honey and/or its derivatives in animals?* The following definitions will be used:

- Natural honey and/or its derivatives: honey and/or honey-derived products obtained from honeybees of the genus *Apis*.
- Medicinal use: includes treatment of disease or medical condition as well as prevention of a disease or medical condition.
- Animals: livestock species, poultry, horses, companion animals, lab animals, exotic species, and wildlife.

We will explore this research question with the intention of mapping the literature on this topic, as well as informing potential future systematic reviews.

Methods

Review Registration

This protocol has been published online on the SYREAF (Systematic Reviews for Animals and Food) website at www.syreaf.org, and is archived in the University of Guelph's institutional repository (The Atrium).

Eligibility Criteria

The following studies will be eligible for inclusion:

- Systematic reviews and primary research studies including: experimental studies, analytical observational studies (cross-sectional, cohort, case-control), case reports, case series;
- Theses and dissertations;
- Conference proceedings, if they are >500 words in length;
- Studies which investigate natural honey and/or its derivatives as a therapeutic or preventive medical intervention in any live animal species.

The following types of articles and literature are ineligible for inclusion: narrative reviews, textbooks, editorials, commentaries, testimonials, letters to the editor. Descriptive primary research studies such as surveys of prevalence or incidence which do not assess an intervention, are not eligible for inclusion. Studies for which the full text in English or French is unavailable will be excluded. No restrictions will be placed on year or location of studies.

Eligible Population of Interest

Any animal species, domestic or wild, are eligible populations, and may include any of the following groups: livestock, poultry, companion animals (i.e. cats, dogs and horses), exotic species (e.g., birds, reptiles), and wildlife. Studies examining the use of natural honey and/or its derivatives as an intervention in lab animal species (e.g., mice, rats) for translational research will also be eligible (i.e., humans may be the target population). Insect species such as honeybees are excluded from our population of interest, as it is not within the scope of our review to identify research investigating the use of honey or honey-derived products in mitigating recent honeybee morbidity and mortality.

Eligible Interventions of Interest

Studies examining natural honey and/or its derivatives as an intervention will be eligible. "Derivatives" includes any compound which is bee-derived, and is also naturally found in honey (e.g., lactic acid bacteria). Bee-derived compounds which are not naturally found in honey are ineligible, thus the following compounds are not eligible interventions: propolis, bee pollen, royal jelly, beeswax, bee bread, bee venom. Artificial honey and/or related compounds which are not produced by bees are ineligible. The intervention must be derived from honeybees of the genus *Apis,* therefore products derived from stingless bees (of the tribe Meliponini) are ineligible. The intervention of interest may represent a preventive intervention (designed to prevent onset of a clinical condition), or a therapeutic intervention (designed to reduce signs, severity, or duration of clinical condition).

Eligible Outcomes of Interest

Four broad categories of outcomes will be considered: clinical, physiological, pathological lesions, and mortality. With the exception of mortality, studies must examine the intervention in live animals: *in vitro* studies or studies which investigate the mechanisms by which honey might treat/prevent a disease/injury will not be eligible. Clinical outcomes represent outcomes which are observable in live animals, some examples of which include pain scores, and time to healing (for wounds). Physiological outcomes encompass all outcomes which are not directly observable in live animals, but which may require specialized equipment or tests. Examples of physiological outcomes include blood glucose, heart rate, and white blood cell count. Pathological lesions include all outcomes which are assessed either via post-mortem examination, or via histological examination of tissue samples. Pathological lesions may be assessed either in live animals or by post-mortem examination of euthanized/deceased animals, but the intervention must have been performed on a live animal. Mortality will also be assessed. Outcomes may be focused on any body system.

Identifying Relevant Studies

An initial search of one electronic database was performed by NAV in July 2019. The search terms from this initial search can be found in Appendix I (**Table 1**). Comprehensive searches will be performed using the McLaughlin Library, University of Guelph and the following databases: AGRICOLA© via ProQuest®, CAB Direct© via CAB Direct, PubMed© via NCBI, the Science Citation Index Expanded (SCI-EXPANDED)™, Emerging Sources Citation Index (ESCI)™, the Zoological Record, and Conference Proceedings Citation Index-Science databases via the Web of Science platform™ (**Table 2**).

The grey literature will also be searched, including Google Scholar (first 500 citations sorted on relevance), as well as theses and dissertations using ProQuest Dissertations and Theses Database. If fewer than 50 full text articles are identified as relevant for inclusion, reference list checking will be performed on all relevant articles. If more than 50 full text articles are identified as relevant, reference list checking will only be performed on the 50 most recently published articles.

The search terms will include variations on the concepts "honey" and "intervention", and will be modified for each search resource, accounting for differences in syntax, indexing, and functionality. Controlled vocabulary will be used in the search, when applicable.

There will be no language restrictions on the literature search.

Data Management

Search results will be uploaded into Mendeley® reference management software, and duplicate records will be removed. In the final report, information regarding the total number of unique articles, number of duplicates, and number of eligible studies will be presented using the Preferred Reporting Items for Systematic Reviews and Meta-Analyses (PRISMA)© flowchart template.

Study Selection

Level 1 and **Level 2** screening for eligibility will be performed independently by NAV and a second reviewer. All reviewers will receive training prior to the screening process. A pretest using titles and abstracts of 100 articles will be performed.

Level 1: Title/Abstract screening will be performed using the following questions, with response options "yes", "no", and "unclear":

1) Does the title/abstract describe primary research or a systematic review?
2) Does the title/abstract describe an investigation of the use of natural honey and/or its derivatives as an intervention in a live animal? (studies assessing mortality are eligible)

Studies will be excluded if both reviewers agree that the answer to at least one question is "no".

Level 2: Secondary screening performed on full text with the following questions will be used with responses "yes" (neutral) or "no" (exclude):

3) Is the full text publication >500 words?
4) Does the full text publication describe a primary research study or a systematic review evaluating an intervention? (exclude descriptive studies)
5) Is the full text article available in English or French?
6) Is the intervention a natural honey and/or a derivative **produced by honeybees (genus *Apis*)**?
7) Is the study evaluating natural honey and/or its derivatives as a therapeutic or preventive intervention in **animals**? (study population must be animals; target population can be humans or animals)
8) Was the intervention performed in **live animals?** (i.e. not an *in vitro* or mechanistic study; studies assessing mortality are eligible)
9) Is the live animal population studied one of the following types of animal: livestock, poultry, horses, companion animals, lab animals, exotic species, wildlife? (exclude if the population is an insect).

Fifteen references will be used for the pre-test of the secondary screening tool. Conflicts regarding eligibility will be resolved by consensus, and a third member will arbitrate if consensus cannot be reached. Level 2 eligibility screening and data extraction on French articles will be completed by a single reviewer (NAV).

Data Extraction Strategy

Full text publications will be acquired through available University of Guelph library resources and uploaded into a review management software program (DistillerSR®, Ottawa, ON, Canada).

Data extraction will be performed using forms developed for this review and pre-tested using five references. The information will be extracted from each study by two reviewers working independently. Categories of the following data were determined *a priori* in this protocol, but additional categories may be added during review process as needed.

General study characteristics: publication year, country in which the study was conducted.

Study population: specific animal species studied (e.g., feline, bovine). Information about whether the animal species was studied for translational research (i.e., with a human target population) will also be extracted.

Intervention investigated: type of intervention (i.e., preventive vs. therapeutic). Intervention characteristics will be extracted (specific type, brand) and will be categorized as: non-medicinal honey, medicinal honey, honey derivatives. Information about the mode of administration will be extracted: oral use vs. topical use.

Outcomes investigated: the body system investigated will be extracted (e.g., gastrointestinal, ocular, cardiovascular, dermatological). The indication for use will also be extracted (e.g., glycemic regulation, antimicrobial, cardiovascular protective, anti-tumour, anti-inflammatory, burns, wound healing). The clinical outcome(s) of interest investigated will be extracted (e.g., clinical, physiological, pathological lesions, mortality).

Study approach: the type of study design will be extracted and categorized as:

- **Descriptive:**
    - Case-series / case reports (study describes medicinal use of honey and/or its derivatives in a single animal, or small number of animals)
- **Analytical:**
    - Experimental study
        - Trial of intervention to prevent (i.e. experiments in controlled settings, field trials with natural disease exposure, and challenge trials)
        - Trial of intervention to treat (i.e. controlled trial, field trial, or clinical trial)
    - Observational study
        - Intervention to prevent

        - Intervention to treat
- **Systematic review:**
    - Prevention
    - Treatment

Results Strategy

Charting the Data

A descriptive summary of study characteristics, study approach, animal species, and details about the intervention and outcomes assessed will be generated.

Collating, Summarizing and Reporting the Results

Figures and tables will be used to summarize and report results. A flow chart figure will be used to present the flow of articles through the review process, including number of articles excluded at each level of screening, and reasons for exclusion. Tables will be used to summarize results by study approach (i.e. descriptive; experimental; analytical observational; systematic review; study approach unclear).

Descriptive and analytic study approaches will be further categorized by the following: year of publication, country of study, species category, intervention category (preventive vs. therapeutic), intervention characteristics, intervention mode of administration, intervention indication, body system studied, along with the outcome(s) assessed.

Discussion

This scoping review will provide a synthesis of primary research investigating the medicinal use of natural honey and/or its derivatives in animal species. Results from this review can be used to inform future primary research in this field, as well as future systematic reviews and meta-analyses on this topic.

Competing Interests

The authors have no competing interests to declare.

Authors' Contributions

NAV will serve as review leader, and therefore will be responsible for coordinating the review, training second reviewers, performing primary and secondary screening, data extraction and analysis, summarizing and collation of results. NAV is responsible for preparing all drafts of the protocol and the final manuscript with input from JMS on content and methodology. JMS will act as methodological expert, serving as a consultant for conducting the review, and will also participate as a reviewer. One to three additional reviewers (including DAJ) with experience in the conduct of reviews will participate in the review, performing primary and secondary screening, and data extraction. All authors will contribute to, and approve the final manuscript.

Acknowledgements

There is no external funding for this review.

References

1. Khan RU, Naz S, Abudabos AM. Towards a better understanding of the therapeutic applications and corresponding mechanisms of action of honey. Environ Sci Pollut R. 2017;24: 27755-27766. DOI:10.1007/s11356-017-0567-0
2. Anand S, Deighton M, Livanos G, Morrison PD, Pang ECK, Mantri, N. Antimicrobial activity of agastache honey and characterization of its bioactive compounds in comparison with important commercial honeys. Front Microbiol. 2019;10:1–16. DOI:10.3389/fmicb.2019.00263
3. Bilsel Y, Bugra D, Yamaner S, Bulut T, Cevikbas U, Turkoglu U. Could honey have a place in colitis therapy? Effects of honey, prednisolone, and disulfiram on inflammation, nitric oxide, and free-radical formation. Dig Surg. 2002;19:306-311. DOI:10.1159/000064580.
4. Ahmed S, Othman NH. Honey as a potential natural anticancer agent: a review of its mechanisms. Evid Based Complement Altern Med. 2013; Article ID 829070, 7 pages. DOI:10.1155/2013/829070
5. Can Z, Yildiz O, Sahin H, Turumtay EA, Silici S, Kolayli S. An investigation of Turkish honeys: their physico-chemical properties, antioxidant capacities and phenolic profiles. Food Chem. 2015;180:133-141. DOI:10.1016/j.foodchem.2015.02.024
6. Wasihun A, Kasa B. Evaluation of antibacterial activity of honey against multidrug resistant bacteria in Ayder Referral and Teaching Hospital, Northern Ethiopia. SpringerPlus 2016;5:842. DOI:10.1186/s40064-016-2493-x
7. Jull AB, Cullum N, Dumville JC, Westby MJ, Deshpande S, Walker N. Honey as a topical treatment for wounds. Cochrane Db Syst Rev. 2015;3: Art. No.:CD005083. DOI:10.1002/14651858.CD005083.pub4
8. Norman G, Christia J, Liu Z, Westby MJ, Jefferies JM, Hudson T, et al. Antiseptics for burns (Review). Cochrane Db Syst Rev. 2017;7: Art. No.:CD011821. DOI:10.1102/14651858.CD011821.pub2
9. Oduwole O, Udoh EE, Oyo-Ita A, Meremikwu MM. Honey for acute cough in children. Cochrane Db Syst Rev. 2018; 4. Art. No.:CD007094. DOI:10.1002/14651858.CD007094.pub5
10. Maruhashi E, Braz BS, Nunes T, Pomba C, Belas A, Duarte-Correia JH, Lourenço AM. Efficacy of medical grade honey in the management of canine otitis externa - a pilot study. Vet Dermatol. 2016;27: 93-e27. DOI:10.1111/vde.12291
11. Olofsson TC, Butler É, Lindholm C, Nilson B, Michanek P, Vásquez A. Fighting off wound pathogens in horses with honeybee lactic acid bacteria. Curr Microbiol. 2016;73:463-473. DOI:10.1007/s00284-016-1080-2
12. Piccart K, Vásquez A, Piepers S, De Vliegher S, Olofsson TC. Short communication: Lactic acid bacteria from the honeybee inhibit the in vitro growth of mastitis pathogens. J Dairy Sci. 2016;99: 2940–2944. DOI:10.3168/jds.2015-10208

Appendix I: Initial search strategy and selected electronic databases

Table 1: Initial search of the literature in one electronic database performed on August 29, 2019: **PubMed via NCBI (**McLaughlin Library, University of Guelph)

#	Searches	Results
1	therapeutic OR therapy OR therapeutically OR therapeutics OR therapies OR therapeutical **[all fields]**	**9718330**
2	preventive OR prevent OR prevention OR preventative OR preventable **[all fields]**	**2129496**
3	medicine OR medicinal OR medicate OR medication OR medicated OR medications OR medicines OR medicating **[all fields]**	**6461735**
4	treatment OR treatments OR treated OR treating OR treat OR treats **[all fields]**	**11210430**
5	intervention OR intervene OR interventions OR intervenes OR intervening **[all fields]**	**7927393**
6	health OR healthy OR healthcare OR healthier OR healthiest **[all fields]**	**5416205**
7	heal OR heals OR healing OR healed OR healer **[all fields]**	**254592**
8	1 OR 2 OR 3 OR 4 OR 5 OR 6 OR 7 **[all fields]**	**18961883**
9	apitherapy **[all fields]**	**198**
10	honey **[all fields]**	**11578**
11	"lactic acid bacter*" AND (honeybee OR "honey bee" OR honey OR Apis OR bee) **[all fields]**	**82**
12	9 OR 10 OR 11 **[all fields]**	**11696**
13	8 AND 12 **[all fields]**	**7291**
14	((("Wounds and Injuries"[Mesh])) AND ("Apitherapy"[Mesh] OR "Honey"[Mesh] OR "faringel" [Supplementary Concept] OR "plukenetione A" [Supplementary Concept] OR "3-(2'-pyrrolidinyl)-kynurenic acid" [Supplementary Concept] OR "apalbumin 1, honeybee" [Supplementary Concept] OR "guanxin suhe wan" [Supplementary Concept] OR "hyenanchin" [Supplementary Concept] OR "Bint al Zahab" [Supplementary Concept] OR "ingenol-3,5,20-triacetate" [Supplementary Concept] OR "5-hydroxymethylfurfural" [Supplementary Concept] OR "Aubang Gahl Soo" [Supplementary Concept] OR "leptosperin" [Supplementary Concept] OR "3,6,7-trimethyllumazine" [Supplementary Concept] OR "AP1-1 polysaccharide" [Supplementary Concept] OR "kyung-ok-ko" [Supplementary Concept])) **[all fields]**	**226**
15	13 OR 14 **[all fields]**	**7298**

Table 2 Platforms and electronic databases which will be used for keyword searches.

**Database**	**Platform:**
PubMed	NCBI©
CABI©	CAB Direct
AGRICOLA	ProQuest®
SCI-EXPANDED	Web of Science™
ESCI	Web of Science™
CPCI-S	Web of Science™
ProQuest Dissertations and Theses	ProQuest®

View publication stats
