# Supplementary material for: A Scoping Review of the Evidence for the Medicinal Use of Natural Honey in Animals
Source: Front Vet Sci. 2021 Jan 18;7:618301. doi: 10.3389/fvets.2020.618301 (PMC7847899; doi:10.3389/fvets.2020.618301)
Supplement: Data Sheet 2 — Appendix B. [file Data_Sheet_2.PDF]

Appendix B
Electronic Searches
Sept. 5, 2019

PUBMED (7306 hits)

(((((("Wounds and Injuries"[Mesh])) AND ("Apitherapy"[Mesh] OR "Honey"[Mesh] OR "faringel"[Supplementary Concept] OR "plukenetione A"[Supplementary Concept] OR "3-(2'-pyrrolidinyl)-kynurenic acid"[Supplementary Concept] OR "apalbumin 1, honeybee"[Supplementary Concept] OR "guanxin suhe wan"[Supplementary Concept] OR "hyenanchin"[Supplementary Concept] OR "Bint al Zahab"[Supplementary Concept] OR "ingenol-3,5,20-triacetate"[Supplementary Concept] OR "5-hydroxymethylfurfural"[Supplementary Concept] OR "Aubang Gahl Soo"[Supplementary Concept] OR "leptosperin"[Supplementary Concept] OR "3,6,7-trimethyllumazine"[Supplementary Concept] OR "AP1-1 polysaccharide"[Supplementary Concept] OR "kyung-ok-ko"[Supplementary Concept]))))) OR (((((apitherapy) OR honey) OR (("lactic acid bacter*" AND (honeybee OR "honey bee" OR honey OR Apis OR bee))))) AND ((((((((preventive OR prevent OR prevention OR preventative OR preventable))) OR ((heal OR heals OR healing OR healed OR healer))) OR ((health OR healthy OR healthcare OR healthier OR healthiest))) OR ((intervention OR intervene OR interventions OR intervenes OR intervening))) OR ((treatment OR treatments OR treated OR treating OR treat OR treats))) OR ((medicine OR medicinal OR medicate OR medication OR medicated OR medications OR medicines OR medicating))) OR ((therapeutic OR therapy OR therapeutically OR therapeutics OR therapies OR therapeutical))))

CAB abstracts (1306 hits approx..)

(((((apitherapy) OR honey) OR (("lactic acid bacter*" AND (honeybee OR "honey bee" OR honey OR Apis OR bee))))) AND ((((((((preventive OR prevent OR prevention OR preventative OR preventable))) OR ((heal OR heals OR healing OR healed OR healer))) OR ((health OR healthy OR healthcare OR healthier OR healthiest))) OR ((intervention OR intervene OR interventions OR intervenes OR intervening))) OR ((treatment OR treatments OR treated OR treating OR treat OR treats))) OR ((medicine OR medicinal OR medicate OR medication OR medicated OR medications OR medicines OR medicating))) OR ((therapeutic OR therapy OR therapeutically OR therapeutics OR therapies OR therapeutical)))) AND (((sc:ft)))

Web of Science (Core collection) (7883 hits)

ALL FIELDS: ((((apitherapy OR honey OR ("lactic acid bacter*" AND (honeybee OR "honey bee" OR honey OR Apis OR bee))) AND ((therap* OR medic* OR treat* OR (intervention OR intervene OR interventions OR intervenes OR intervening) OR health* OR (heal OR heals OR healing))))))
Refined by: DOCUMENT TYPES: (ARTICLE OR LETTER OR REPRINT OR PROCEEDINGS PAPER OR NOTE OR CORRECTION ADDITION OR MEETING ABSTRACT OR EARLY ACCESS OR DATA PAPER OR CORRECTION)
Timespan: All years. **Indexes:** SCI-EXPANDED, CPCI-S, ESCI.

Web of Science (SciELO citation index) (223 hits)

TOPIC: ((((apitherapy OR honey OR ("lactic acid bacter*" AND (honeybee OR "honey bee" OR honey OR Apis OR bee))) AND ((therap* OR medic* OR treat* OR prevent* (intervention OR intervene OR interventions OR intervenes OR intervening) OR health* OR (heal OR heals OR healing))))))
Timespan: All years. **Indexes:** SCIELO.

ProQuest (Dissertations and theses) (443 hits)

(ab(apitherapy OR honey OR ("lactic acid bacter*" AND (honeybee OR "honey bee" OR honey OR Apis OR bee))) AND ab(heal* OR therap* OR medic* OR interven* OR treat* OR prevent*)) OR (ti(apitherapy OR honey OR ("lactic acid bacter*" AND (honeybee OR "honey bee" OR honey OR Apis OR bee))) AND ti(heal* OR therap* OR medic* OR interven* OR treat* OR prevent*))

ProQuest (AGRICOLA) (2750 hits)

((apitherapy OR honey OR ("lactic acid bacter*" AND (honeybee OR "honey bee" OR honey OR Apis))) AND (therap* OR medic* OR treat* OR prevent* OR health* OR (heal OR heals OR healing) OR (intervention OR intervene OR interventions OR intervenes OR intervening))) OR (su(honey) AND (su(heal*) OR su(interven*) OR su(therap*) OR su(treat*) OR su(medic*) OR su(prevent*)))

First 500 relevant articles from Google Scholar

(honey OR ("lactic acid bacteria" AND (honey OR honeybee OR "honey bee" OR Apis OR bee))) AND (heal* OR interven* OR therap* OR treat* OR prevent* OR medic*) AND veterinar*

A total of 20,411 articles with duplicates
