# Supplementary material for: A Scoping Review of the Evidence for the Medicinal Use of Natural Honey in Animals
Source: Front Vet Sci. 2021 Jan 18;7:618301. doi: 10.3389/fvets.2020.618301 (PMC7847899; doi:10.3389/fvets.2020.618301)
Supplement: Data Sheet 3 — Appendix C. [file Data_Sheet_3.PDF]

Appendix C
Level 1 and Level 2 Screening Forms and Guidelines

Level 1

1. Does the title/abstract describe **primary research** or a **systematic review**?
   Answers (select one): *yes, no, unsure*
2. Does the title/abstract describe an investigation of the **use of natural honey** and/or its derivatives as an intervention in a **live animal?** (studies assessing mortality are eligible)
   Answers (select one): *yes, no, unsure*

Level 2

1. Is the article in **English** or **French?**
   Answers (select one): *yes, no*
2. Is the full text publication **>500** words?
   Answers (select one): *yes, no*
3. Does the publication describe a **primary research study** or a **systematic review?**
   Answers (select one): *yes, no*
4. Was the intervention performed in **LIVE animals**? (exclude insects)
   Answers (select one): *yes, no*
5. Is the intervention a natural honey and/or a honey derivative **produced by honeybees (genus *Apis*)?**
   Answers (select one): *yes, no*
6. Is natural honey and/or its derivatives being investigated as a **therapeutic** or **preventive** intervention in animals?
   Answers (select one): *yes, no*

<u>Additional guidelines:</u>

- Study population must be animals; target population can be humans or animals.
- Studies assessing mortality are eligible.
- In vitro or mechanistic studies are **NOT** eligible. Exclude at question 4.
- Assume that honey derivatives listed with a manufacturer are synthetically derived and exclude at question 5.
- Examples of honey derivatives include: chrysin, lactic acid bacteria, methylglyoxal, caffeic acid.
- Honey may be used alone, mixed with other bee products, or mixed with other medicinal substances such as herbs.
- Studies which use honey as a **vehicle** are **NOT** eligible. Exclude at question 6.
- **Pharmacokinetic** studies which examine the interaction of honey with other medications are **NOT** eligible. Exclude at question 6.
- For question 6, the study must be evaluating a positive effect of the intervention. Studies examining the toxicity of mad honey are not eligible. Exclude at question 6.

- For question 6, interventions used as a proof of concept for health improvement in the absence of disease or injury are ineligible.
- For question 6, studies investigating the prevention or treatment of toxin exposures are considered eligible:
    - E.g., Studies examining formaldehyde-induced inflammation in the paws of mice are considered eligible, since this scenario represents a toxin exposure for which honey might serve as a treatment. In contrast, studies examining honey as an intervention for the reduction of inflammation in the absence of a toxin exposure or disease are ineligible.
