# Supplementary material for: A Scoping Review of the Evidence for the Medicinal Use of Natural Honey in Animals
Source: Front Vet Sci. 2021 Jan 18;7:618301. doi: 10.3389/fvets.2020.618301 (PMC7847899; doi:10.3389/fvets.2020.618301)
Supplement: Data Sheet 4 — Appendix D. [file Data_Sheet_4.PDF]

Appendix D
Data Extraction Form and Guidelines

1. Year of publication: _________
2. Country of study – as reported, or if not reported, country of corresponding author affiliation: _____________
3. Animal species (at species level): _________________
4. What study design was used (based on what they did, **not** based on how it was reported)? Answers (select one): *Case report/case series, experimental-deliberate disease model, experiment-natural exposure, observational, systematic review, scoping review*
5. Was this translational research (i.e., an animal model for evaluating potential treatments for humans)
   Answers (select one): *yes, no*
6. Was it intended to prevent or treat (therapeutic)?
   Answers (select one): *prevent, treat*
7. Were there multiple interventions with honey and/or honey derivatives? (e.g., head-to-head comparison of different honeys against a control group?)
   Answers (select one): *yes, no*

   If answer to Q7 was yes, *then:*
8. How many unique honey and/or honey derivative interventions were studied?
   Answers (select one): *2, 3, 4, 5, 6*
9. For the **first** intervention (alphabetically), is honey a component (alone or in combination) of the intervention?
   Answers (select one): *yes, no*

   If answer to Q9 was yes, *then:*
10. For the first intervention (alphabetically), was it a commercial medical honey?
    Answers (select one): *yes (specify product name) ________ , no (specify label if provided, e.g., "Turkish honey")*
11. For the first intervention (alphabetically), is a honey derivative a component (along or in combination) of the intervention?
    Answers (select one): *yes (specify which derivative it was, e.g., chrysin) _________, no*
12. Were there other components to the first intervention?
    Answers (select one): *yes, no*

    If answer to Q12 was yes, *then:*
13. What were the other components to the first intervention?
    Answers (select all that apply): *herbal product, drug, other bee products (specify, e.g., bee venom, propolis, bee pollen, royal jelly, beeswax, bee bread) _________, other (specify)____________.*

    Depending on answer to Q8:
    Identical questions and logic as used for questions 9-13 were used for additional unique interventions, representing questions 14-38 (up to 6 unique interventions, if applicable).

If answer to Q7 was no*, then:*
Identical questions and logic as used for questions 9-13 were used to capture details of the intervention, representing questions 39-43.

44. What was the mode of administration of the intervention?
Answers (select one): *intravenously, topical (on skin), oral, intraperitoneal (body cavity), ocular (eye), otic (ear), other (specify)* _________.
45. What body system was the intervention being used to treat? Please specify the condition when reported (e.g., Burn management would be checked as **dermatological** with "burns" in text box).
Answers (select all that apply): *gastrointestinal* _________*, musculoskeletal* _________*, nervous system* _________*, cardiovascular* _________*, lymphatic/immune* _________*, endocrine/metabolic* _________*, urinary/renal* _________*, reproductive* _________*, dermatological* _________*, other* ________.
46. What outcomes were measured?
Answers (select all that apply): *clinical outcomes (e.g., wound size), physiological (e.g., heart rate), pathological (e.g., histology, post-mortem observations), mortality.*

Additional guidelines:

- Separate trials within a publication were extracted on separate forms. Trials using more than one mode of administration were also extracted on separate forms.
- When more than one intervention was conducted on the same group of animals at different points in time, this was considered a single study. For instance, a study that examined nociception in mice and which subsequently used the same group of mice to study carrageenan-induced paw edema was classified as one trial, and extracted on the same form.
- If multiple animal species received the same intervention(s), these were extracted on the same form.
- To determine what study design was used the following definitions were provided (using wound healing in animals as an example):
  - Case series/case report: animals with wounds were treated with honey. **No control group.**
  - Experimental – natural exposure: animals with wounds were **randomly assigned** by researcher to either a treatment group (honey) or control group. Usually a field study – owners don't decide which group animal is in.
  - Experiment – deliberate disease: usually lab studies. Animals were inoculated with wounds, and then **randomly assigned** by researcher to either a treatment group (honey) or control group.
  - Observational: animals with wounds are already being treated with honey as decided by owners and we have decided to observe the study as it unfolds – **must have a control group.**
- For question 6, answer "prevent" if the intervention is used to prevent a disease/condition from occurring. Answer "treat" if the intervention is used to minimize/address an existing disease/condition. Studies which do not prevent or

treat a well-recognized and uncontroversial disease/condition **should have been excluded at level 2.** E.g., amelioration of lipid profile (such as cholesterol levels) is not considered a disease/condition for the purposes of our review.

- Treatment groups with different honey interventions should be extracted separately, (even if they only differ by an extra component such as herbs or drugs). If interventions differ only with respect to dosage, collapse these groups, and extract as one intervention.
- If the type of non-medical honey is not specified, leave the text box blank.
- Many of the interventions for wound healing will describe how the wounds were flushed and cleaned prior to treatment and bandaged following treatment. These are considered standard approaches to wound management and we are not interested in capturing these details: only capture details you would expect to vary between studies. E.g., if antibiotics were administered along with honey, we would capture this by selecting “drug” because we do not anticipate this to be given within all studies of wound healing in animals. Another E.g., if banana leaves were used as a bandage, and this is a novel approach, select “other” and specify in text box that “banana leaves as bandage”.
- If a condition/illness falls under more than one body system, please select the most appropriate one. E.g., report polycystic ovarian syndrome under “endocrine”, rather than “reproductive”. Studies evaluating the effect of toxins primarily on one or two body systems should be classified under that body system: for these studies, report both the toxin being used and the induced disease within the text box, e.g., “carbon tetrachloride-induced hepatotoxicity”.
- Classify hepatic and pancreatic diseases under “gastrointestinal system”.
- If an animal disease model is being used to approximate a human condition, extract the human disease, not the reported disease in the animal model.
- The following are examples of conditions/illnesses and their classification:
    - Gastrointestinal: anastomosis surgery, irritable bowel disease
    - Musculoskeletal: bone graft, fracture
    - Nervous system: pain, spinal surgery
    - Cardiovascular: arrhythmias
    - Lymphatic/immune: rheumatoid arthritis, bacterial infection
    - Endocrine/metabolic: diabetes, hyperthyroidism
    - Urinary/renal: kidney stones
    - Reproductive: infertility due to noise stress
    - Dermatological: wound healing, burns, eczema
    - Other: tumour reduction, tumour growth
        - Extract as “wound healing” for all studies examining the healing of wounds, regardless of additional features such as bacterial infection, or comorbidities.
        - Specify “burns” (if applicable) if this is the type of wound. All other types of wounds will be extracted as “wound healing”.
- Clinical outcomes and physiological outcomes are always measured in a live animal. Clinical outcomes are readily observable, either with a physical examination, or with simple measurement (e.g., body weight, wound size). Outcomes assessed in a live animal to assess physiology, and requiring

specialized equipment are physiological (e.g., heart rate). If an outcome is measured in a deceased/euthanized animal, it is necessarily pathological. Tissue, blood or other samples removed from the animal for assessment are classified as pathological outcomes. A post-mortem examination would be classified as a pathological outcome.
