# Supplementary material for: A Scoping Review of the Evidence for the Medicinal Use of Natural Honey in Animals
Source: Front Vet Sci. 2021 Jan 18;7:618301. doi: 10.3389/fvets.2020.618301 (PMC7847899; doi:10.3389/fvets.2020.618301)
Supplement: Data Sheet 5 — Appendix E. [file Data_Sheet_5.PDF]

Appendix E
Citations Included

Veterinary Research Publications

1. Abdel-Wahed, R. E., El-Kammar, M. H., Abu-Ahmed, H., & Abo-Bakr, E. (2011). Propolis (bee glue)... a new dressing for equine wounds. *Alexandria Journal of Veterinary Sciences*, *34*(1), 21–36.
2. Abu-Ahmed, H., Abdel-Wahed, R. E., El-Kammar, M. H., & El-Neweshy, M. S. (2013). Evaluation of the effectiveness of propolis compared with honey on second intention wound healing in the equine. *Middle East Journal of Scientific Research*, *14*(10), 1292–1298. https://doi.org/10.5829/idosi.mejsr.2013.14.10.7494
3. Adeyemi, A. B., Jegede, H. O., Amid, A. S., Daodu, O. B., Ajiboye, B., Hamzat, M. O., Durotoye, T., Amire, E. S., Bashir, M. D., & Adeyanju, J. B. (2017). Management of a chronic necrotizing wound in a dog using natural honey therapy. *Exploratory Animal and Medical Research*, *7*(2), 201–205.
4. Ahmed M, B. S. (2012). Wound care with euphorbia honey after nucleation: a case report. *Clinical Microbiology: Open Access*, *02*(06). https://doi.org/10.4172/2327-5073.1000129
5. Ali, M. M., & Radad, K. (2011). Cod liver oil/honey mixture: an effective treatment of equine complicated lower leg wounds. *Veterinary World*, *4*(7), 304–310.
6. Apaydin, N., Kemiksiz, E., & Akcay, A. (2019). Comparison of manuka honey (Manuka Nd, G) and Etacridine Lactate (Rivanol) applications in the treatment of infected wounds in cats. *Acta Scientiae Veterinariae*, *47*(1), 1–8. https://doi.org/10.22456/1679-9216.90287
7. Babaei, S., Rahimi, S., Torshizi, M. A. K., Tahmasebi, G., & Miran, S. N. K. (2016). Effects of propolis, royal jelly, honey and bee pollen on growth performance and immune system of Japanese quails. *Veterinary Research Forum*, *7*(1), 13–20.
8. Bischofberger, A. S., Dart, C. M., Horadagoda, N., Perkins, N. R., Jeffcott, L. B., Little, C. B., & Dart, A. J. (2016). Effect of Manuka honey gel on the transforming growth factor β1 and β3 concentrations, bacterial counts and histomorphology of contaminated full-thickness skin wounds in equine distal limbs. *Australian Veterinary Journal*, *94*(1–2), 27–34. https://doi.org/10.1111/avj.12405
9. Bischofberger, A. S., Tsang, A. S., Horadagoda, N., Dart, C. M., Perkins, N. R., Jeffcott, L. B., Jackson, C. J., & Dart, A. J. (2015). Effect of activated protein C in second intention healing of equine distal limb wounds: a preliminary study. *Australian Veterinary Journal*, *93*(10), 361–366. https://doi.org/10.1111/avj.12363
10. Bischofberger, A. S., Dart, C. M., Perkins, N. R., & Dart, A. J. (2011). A preliminary study on the effect of manuka honey on second-intention healing of contaminated wounds on the distal aspect of the forelimbs of horses. *Veterinary Surgery*, *40*(7), 898–902. https://doi.org/10.1111/j.1532-950X.2011.00886.x
11. Bischofberger, A. S., Dart, C. M., Perkins, N. R., Kelly, A., Jeffcott, L., & Dart, A. J. (2013). The effect of short- and long-term treatment with manuka honey on second intention healing of contaminated and noncontaminated wounds on the distal aspect of the forelimbs in horses. *Veterinary Surgery*, *42*(2), 154–160. https://doi.org/10.1111/j.1532-950X.2012.01083.x

12. Chaudhary, P. S., Varshney, J. P., & Deshmukh, V. V. (2011). Emergency and critical care of thermal burns in bovines. *Intas Polivet*, *12*(2), 172–179.
13. Eyarefe, O., & Oguntoye, C. (2017). Honey, an unexplored topical wound dressing agent in Nigerian veterinary practice. *Sokoto Journal of Veterinary Sciences*, *14*(3), 8. https://doi.org/10.4314/sokjvs.v14i3.2
14. Gakuya, D. W., Mulei, C. M., & Wekesa, S. B. (2011). Use of ethnoveterinary remedies in the management of foot and mouth disease lesions in a dairy herd. *African Journal of Traditional, Complementary and Alternative Medicines*, *8*(2), 165–169. https://doi.org/10.4314/ajtcam.v8i2.63204
15. Ferreira, D., Rocha, H. C., Kreutz, L. C., Loro, V. L., Marqueze, A., Koakoski, G., Santos da Rosa, J. G., Gusso, D., Oliveira, T. A., de Abreu, M. S., & Barcellos, L. J. G. (2013). Bee products prevent agrichemical-induced oxidative damage in fish. *PLoS ONE*, *8*(10). https://doi.org/10.1371/journal.pone.0074499
16. Ferreira, M. P., Alievi, M. M., Beck, C. A. C., Dal-Bó, I. S., Gonzalez, P. C., Nóbrega, F. S., Silva, L. M., Stédile, R., Scherer, S., Rocha, J. P. V, Silva Filho, A. P. F., Svierk, B., & Costa, G. (2012). Comparison of lyophilization, and freezing in honey as techniques to preserve cortical bone allografts used to repair experimental femoral defects in domestic adult cats. *Arquivo Brasileiro de Medicina Veterinária e Zootecnia*, *64*(2), 263–273. https://doi.org/10.1590/S0102-09352012000200003
17. Herbert, E. W. (2018). Findings and strategies for treating horses injured in open range fires. *Equine Veterinary Education*, *30*(4), 177–186. https://doi.org/10.1111/eve.12806
18. Herdan, C. L., Acke, E., Dicken, M., Archer, R. M., Forsyth, S. F., Gee, E. K., & Pauwels, F. E. T. (2012). Multi-drug-resistant *Enterococcus* spp. as a cause of non-responsive septic synovitis in three horses. *New Zealand Veterinary Journal*, *60*(5), 297–304. https://doi.org/10.1080/00480169.2011.651702
19. Maguire, P., Azagrar, J. M., Carb, A., & Lesser, A. (2015). The successful use of negative-pressure wound therapy in two cases of canine necrotizing fasciitis. *Journal of the American Animal Hospital Association*, *51*(1), 43–48. https://doi.org/10.5326/JAAHA-MS-6033
20. Lukanc, B., Potokar, T., & Erjavec, V. (2018). Observational study of the effect of L-Mesitran® medical honey on wound healing in cats. *Veterinarski Arhiv*, *88*(1), 59–74. https://doi.org/10.24099/vet.arhiv.160905a
21. Mandel, H. H., Sutton, G. A., Abu, E., & Kelmer, G. (2020). Intralesional application of medical grade honey improves healing of surgically treated lacerations in horses. *Equine Veterinary Journal*, *52*(1), 41–45. https://doi.org/10.1111/evj.13111
22. Maruhashi, E., Braz, B.S., Nunes, T., Pomba, C., Belas, A., Duarte-Correia, J.H. and Lourenço, A.M. (2016), Efficacy of medical grade honey in the management of canine otitis externa – a pilot study. Vet Dermatol, 27: 93-e27. https://doi.org/10.1111/vde.12291
23. Ranjan, R., Biswal, J. K., Sharma, A. K., Kumar, M., & Pattnaik, B. (2016). Management of foot and mouth disease in a dairy farm: By ethnoveterinary practice. *Indian Journal of Animal Sciences*, *86*(3), 256–259.
24. Ogunro, B.N, Otuh, P.I., Olatunji-Akioye, A., Emikpe, B.O., Oyebanji, V., Adejumodi, O., & Morenikeji, O. (2016). Management of epitheliogenesis imperfecta in a piglet (*Sus Scrofa domesticus)* in Ibadan, Nigeria. *Nigerian Veterinary Journal*, *37*(3), 187-191–191.

25. Royaux, E., Polis, I., Boyen, F., Van Ham, L., & de Rooster, H. (2016). No evidence that medicinal honey reduces bacterial skin colonisation at a peripheral catheter insertion site in dogs. *Journal of Small Animal Practice*, *57*(7), 374–378. https://doi.org/10.1111/jsap.12501
26. Skinner, O. T., Cuddy, L. C., Coisman, J. G., Covey, J. L., & Ellison, G. W. (2016). Temporary rectal stenting for management of severe perineal wounds in two dogs. *Journal of the American Animal Hospital Association*, *52*(6), 385–391. https://doi.org/10.5326/JAAHA-MS-6350
27. Sogebi, E. A. O., Adeleye, A. I., & Babalola, S. A. (2017). Management of partial thickness burn of the dorsum skin in a 3-year-old male German shepherd. *Sokoto Journal of Veterinary Sciences*, *15*(1), 77–81. https://doi.org/10.4314/sokjvs.v15i1.11
28. Staunton, C. J., Halliday, L. C., & Garcia, K. D. (2005). The use of honey as a topical dressing to treat a large, devitalized wound in a stumptail macaque (*Macaca arctoides*). *Contemporary Topics in Laboratory Animal Science*.
29. Tsang, A. S., Dart, A. J., Sole-Guitart, A., Dart, C. M., Perkins, N. R., & Jeffcott, L. B. (2017). Comparison of the effects of topical application of UMF20 and UMF5 manuka honey with a generic multifloral honey on wound healing variables in an uncontaminated surgical equine distal limb wound model. *Australian Veterinary Journal*, *95*(9), 333–337. https://doi.org/10.1111/avj.12616
30. Wahba, N. M., El Nisr, N. A., Sayed, S. M., Ellah, M. R. A., El-Hafeez, M. M. A., & Aamer, A. A. (2011). Intramammary honey infusion: a new trend in the management of bovine subclinical mastitis. *Journal of Animal and Veterinary Advances*, *10*(20), 2740–2744. https://doi.org/10.3923/javaa.2011.2740.2744
31. Olofsson, T. C., Butler, É., Lindholm, C., Nilson, B., Michanek, P., & Vásquez, A. (2016). Fighting off wound pathogens in horses with honeybee lactic acid bacteria. *Current Microbiology*, *73*(4), 463–473. https://doi.org/10.1007/s00284-016-1080-2
32. Oelschlaegel, S., Pieper, L., Staufenbiel, R., Gruner, M., Zeippert, L., Pieper, B., Koelling-Speer, I., & Speer, K. (2012). Floral markers of cornflower (*Centaurea cyanus*) honey and its peroxide antibacterial activity for an alternative treatment of digital dermatitis. *Journal of Agricultural and Food Chemistry*, *60*(47), 11811–11820. https://doi.org/10.1021/jf303699t
33. Khiati, B., Bacha, S., Aissat, S., & Ahmed, M. (2014). The use of Algerian honey on cutaneous wound healing: a case report and review of the literature. *Asian Pacific Journal of Tropical Disease*, *4*(S2), S867–S869. https://doi.org/10.1016/S2222-1808(14)60748-9
34. Rusu, S. M., & Öfner, S. (2017). A case study on thermographical aspects of plastron wound in a spur-thighed tortoise (*Testudo graceca*). *Revista Română de Medicină Veterinară*, *27*(1), 16-18.
35. Hananeh, W. M., Ismail, Z. B., Alshehabat, M. A., Abeeleh, M. A., & Ali, J. H. (2015). Effects of Sidr honey on second-intention healing of contaminated full-thickness skin wounds in healthy dogs. *Bulletin of the Veterinary Institute in Pulawy*, *59*(3), 433–439. https://doi.org/10.1515/bvip-2015-0063
36. Wilson C. R. (2013). Feline gangrenous mastitis. *The Canadian veterinary journal = La revue veterinaire canadienne*, *54*(3), 292–294.
37. Ferreira, M. P., Alievi, M. M., Dal-Bó, I. S., Gonzalez, P., Nóbrega, F. S., Gouvêa, A. S., & Beck, C. (2018). Surgical management of long bone fractures in cats using cortical

bone allografts preserved in honey. *The Canadian veterinary journal = La revue veterinaire canadienne*, *59*(4), 393–396.
38. Adekunle, M. O., Abioja, M. O., Abiona, J. A., Jegede, A. V, & Sodipe, O. G. (2017). Rectal temperature, heart rate, packed cell volume and differential white blood cell count of laying pullets to honey supplemented water during hot-dry season. *Slovak Journal of Animal Science*, *50*(1), 15–20.
39. Shrestha, R. (2017). A case report on wound healing activity of honey dressing. *Nepalese Journal of Agricultural Sciences,* 15, 202-204.
40. Abdel-Wahed, R.E., El-Kammar, M.H., Korittum, A.S., & Edrees, I.R. (2013). Surgical and histological evaluation of the effectiveness of propolis on wound healing. *Alexandria Journal of Veterinary Sciences*, *39*(1), 52–63.
41. Shokouhi Sabet Jalali, F., Farshid, A.A., Saifzadeh, S., & Javanmardi, S. (2006). Evaluation of topical application of honey in prevention of post-operative peritoneal adhesion formation in dogs. *Iranian Journal of Veterinary Research, 7*(4), 59-62.
42. Alshehabat, M. A., Ismail, Z. A. B., Hananeh, W. M., & Awawdeh, M. S. (2016). Effect of sidr honey on healing of contaminated wounds in alloxan-induced diabetes mellitus in dogs. *Research Opinions in Animal & Veterinary Sciences*, *6*(8), 242–247. https://doi.org/10.20490/roavs/16-041
43. Kumar, A., Dass, L.L., Singh, K.K., & Sahay, S. (2005). Histomorphological evaluation of wound healing potential of honey. *Royal Veterinary Journal of India, 1*(2), 78-80.
44. Abdullah, S., Leghari, I. H., Moriani, A. A., Rajput, N., Gandhai, J. A., & Nisa, M. (2018). Effect of in ovo supplementation of honey in fertile eggs on post hatch growth performance of broiler chickens. *Journal of Animal and Plant Sciences*, *28*(6), 1584–1590.
45. Devi Prasad, V., Hari Krishna, N.V.V., & Ravi Kumar, P. (2018). Clinical management of burn- a report of six ongole cattle. *Intas Polivet, 19*(1), 78-80.
46. Adebiyi, O. A., Okolie-Alfred, U. V, Godstime, C., & Adeniji, O. A. (2015). Performance and gut morphometry of broiler fed maize based diets supplemented with charcoal and honey as anti-aflatoxin. *American Journal of Experimental Agriculture*, *7*(3), 163–169. https://doi.org/10.9734/AJEA/2015/13012
47. Rosidah, R., Subhan, U., Mulyani, Y., & Dermawan, R. (2019). The effectiveness of honey supplementation in feed for improving goldfish fingerling *Carassius auratus* immune system against *Aeromonas hydrophila* bacteria attack. *Jurnal Akuakultur Indonesia, 18*(1), 89-100. https://doi.org/10.19027/jai.18.1.89-100

Biomedical Research Publications

48. Ali, A. R. A., & Ismail, S. H. (2012). The protective effect of honey against amikacin-induced nephrotoxicity in rats. *Iraqi Journal of Pharmaceutical Sciences*, *21*(2), 85-93.
49. Aziz, C., Ismail, C., Hussin, C., & Mohamed, M. (2014). The antinociceptive effects of tualang honey in male Sprague-Dawley rats: A preliminary study. *Journal of Traditional and Complementary Medicine*, *4*(4), 298–302. https://doi.org/10.4103/2225-4110.139115
50. Doğan, A., & Kolankaya, D. (2005). Protective effect of Anzer honey against ethanol-induced increased vascular permeability in the rat stomach. *Experimental and Toxicologic Pathology*, *57*(2), 173–178. https://doi.org/10.1016/j.etp.2005.04.004

51. Gunaldi, O., Erdogan, S., Guclu, D. G., Tugcu, B., Ofluoglu, E., Baydin, S., & Emel, E. (2014). "Honey" can prevent epidural fibrosis development after laminectomy: An experimental study. *Turkish Neurosurgery*, *24*(6), 849–854. https://doi.org/10.5137/1019-5149.JTN.8783-13.0
52. Kumar Saxena, A., P Phyu, H., M Al-Ani, I., & P, O. (2016). Improved spatial learning and memory performance following Tualang honey treatment during cerebral hypoperfusion-induced neurodegeneration. *Journal of Translational Science*, *2*(5), 264–271. https://doi.org/10.15761/jts.1000150
53. Mahaneem, M., Sulaiman, S. A., Jaafar, H., Nainamohamed, K., Sirajudeen, S., Ismail, Z. I. M., & Islam, M. N. (2011). Effect of honey on testicular functions in rats exposed to cigarette smoke. *Journal of ApiProduct and ApiMedical Science*, *3*(1), 12–17. https://doi.org/10.3896/ibra.4.03.1.03
54. Mohamed, M., Sulaiman, S. A., Jaafar, H., & Salam, K. N. (2011). Antioxidant protective effect of honey in cigarette smoke-induced testicular damage in rats. *International Journal of Molecular Sciences*, *12*(9), 5508–5521. https://doi.org/10.3390/ijms12095508
55. Nur Jannah, M. H., Mahmood, A. A., Sidik, K., & Salmah, I. (2006). Cytoprotective effects of honey in combination with Aqueous and ethanol extracts from *Chromolaena odorata l.* (*eupatorium odoratum l.*) in rats. *Journal of Health and Translational Medicine*, *9*(1), 7–13.
56. Öztaşan, N., Altinkaynak, K., Akçay, F., Göçer, F., & Dane, Ş. (2005). Effects of mad honey on blood glucose and lipid levels in rats with streptozocin-induced diabetes. *Turkish Journal of Veterinary and Animal Sciences*, *29*(5), 1093–1096. https://www.researchgate.net/publication/287862277
57. Sairazi, N. S. M., Sirajudeen, K. N. S., Muzaimi, M., Swamy, M., Asari, M. A., & Sulaiman, S. A. (2017). Tualang honey attenuates kainic acid-induced oxidative stress in rat cerebellum and brainstem. *International Journal of Pharmacy and Pharmaceutical Sciences*, *9*(12), 155-162. https://doi.org/10.22159/ijpps.2017v9i12.21084
58. Demir, A., Simsek, T., Engin, M.S., & Yildiz, L. (2007). The effect of topical honey wound dressing on wound healing in diabetic mice. *Gazi Medical Journal, 18*(3), 110-113.
59. Sidik, K., Mahmood, A.A., & Salmah, I. (2006). Acceleration of wound healing by aqueous extract of *Allium sativum* in combination with honey on cutaneous wound healing in rats. *International Journal of Molecular Medicine and Advance Sciences, 2*(2), 231-235.
60. Rozaini, M.Z., Zuki, A.B.Z., Noordin, M., Norimah, Y., & Nazrul Hakim, A. (2004). The effects of different types of honey on tensile strength evaluation of burn wound tissue healing. *International Journal of Applied Research in Veterinary Medicine, 2*(4), 290-296.
61. Adnyana, I. K., Sigit, J. I., & Kusumawardani, L. A. (2014). Gastric ulcer healing effect of wild honey and its combination with Turmeric (*Curcuma domestica* Val.) Rhizome on male Wistar rats. *Journal of Chinese Pharmaceutical Sciences*, *23*(12), 844–849. https://doi.org/10.5246/jcps.2014.12.107
62. Ali, A. T. M. M., & Al Swayeh, O. A. (2003). Honey potentiates the gastric protection effects of sucralfate against ammonia-induced gastric lesions in rats. *Saudi Journal of Gastroenterology*, *9*(3), 117–123.

63. Alturkistani, H. A., Abuzinadah, O. A. H., Kelany, A. M., Abd El-Aziz, G. S., & Alrafiah, A. R. (2019). The combined effect of honey and olive oil against methotrexate mediated hepatotoxicity in rats: A biochemical, histological and immunohistological study. *Histology and Histopathology*, *34*(12), 1313–1327. https://doi.org/10.14670/HH-18-126
64. Andriţoiu, C. V., Andriţoiu, V., Cuciureanu, M., Nica-Badea, D., Bibire, N., & Popa, M. (2014). Effect of apitherapy products against carbon tetrachloride-induced toxicity in Wistar rats. *Romanian Journal of Morphology and Embryology*, *55*(3), 835–847.
65. Asgari, B., Kermanian, F., Derakhshan, N., Asna-Ashari, M., Sadat, Z. R. N., & Yaslianifard, S. (2018). Honey-derived *Lactobacillus rhamnosus* alleviates *Helicobacter pylori*-induced gastro-intestinal infection and gastric inflammation in C57BL/6 mice: an immuno-histologic study. *Arquivos de Gastroenterologia*, *55*(3), 279–282. https://doi.org/10.1590/s0004-2803.201800000-70
66. Azman, K. F., Zakaria, R., AbdAziz, C., Othman, Z., & Al-Rahbi, B. (2015). Tualang honey improves memory performance and decreases depressive-like behavior in rats exposed to loud noise stress. *Noise and Health*, *17*(75), 83–89. https://doi.org/10.4103/1463-1741.153388
67. Bigham-Sadegh, A., Karimi, I., Hoseini, F., Oryan, A., Sharifi, S., & Pakzad, A. (2018). Effects of honey and hydroxyapatite on bone healing in rats. *Trauma Monthly*, *23*(4):e56119. https://doi.org/10.5812/traumamon.56119
68. Bilsel, Y., Bugra, D., Yamaner, S., Bulut, T., Cevikbas, U., & Turkoglu, U. (2002). Could honey have a place in colitis therapy? Effects of honey, prednisolone, and disulfiram on inflammation, nitric oxide, and free radical formation. *Digestive Surgery*, *19*(4), 306–311. https://doi.org/10.1159/000064580
69. Chamani, G., Zarei, M. R., Mehrabani, M., Mehdavinezhad, A., Vahabian, M., & Ahmadi-Motamayel, F. (2017). Evaluation of honey as a topical therapy for intraoral wound healing in rats. *Wounds*, *29*(3), 80–86.
70. El-haskoury, R., Al-Waili, N., Kamoun, Z., Makni, M., Al-Waili, H., & Lyoussi, B. (2018). Antioxidant activity and protective effect of carob honey in CCl_4-induced kidney and liver injury. *Archives of Medical Research*, *49*(5), 306–313. https://doi.org/10.1016/j.arcmed.2018.09.011
71. Eroglu, O., Deniz, T., Kisa, U., Comu, F. M., Kaygusuz, S., & Kocak, O. M. (2018). The effect of different types of honey on healing infected wounds. *Journal of Wound Care*, *27*(10), S18–S25. https://doi.org/10.12968/jowc.2018.27.Sup10.S18
72. Giusto, G., Vercelli, C., Comino, F., Caramello, V., Tursi, M., & Gandini, M. (2017). A new, easy-to-make pectin-honey hydrogel enhances wound healing in rats. *BMC Complementary and Alternative Medicine*, *17*(1), 1–7. https://doi.org/10.1186/s12906-017-1769-1
73. Karabulut, E., & Durmus, A. S. (2014). Use of honey in treatment of cornea alkali burns. *Indian Veterinary Journal*, *81*, 993-994.
74. Middelkoop, E., Van Den Bogaerdt, A. J., Lamme, E. N., Hoekstra, M. J., Brandsma, K., & Ulrich, M. M. W. (2004). Porcine wound models for skin substitution and burn treatment. *Biomaterials*, *25*(9), 1559–1567. https://doi.org/10.1016/S0142-9612(03)00502-7
75. Milani, S. M., Najafi, M., Alizadeh, P., & Rezazadeh, H. (2018). Inhibitory effect of Honey on 7,12-Dimethylbenz(a)anthracene-initiated and croton oil-promoted skin

carcinogenesis. *Jundishapur Journal of Natural Pharmaceutical Products*, *13*(3):e57992. https://doi.org/10.5812/jjnpp.57992

76. Mohamad Zaid, S. S., Kassim, N. M., & Othman, S. (2015). Tualang honey protects against BPA-induced morphological abnormalities and disruption of ERα, ERβ, and C3 mRNA and protein expressions in the uterus of rats. *Evidence-Based Complementary and Alternative Medicine*, 202874. https://doi.org/10.1155/2015/202874
77. Mohd. Zohdi, R., Zuki Abu Bakar Zakaria, M., Yusof, N., Mustapha, N. M., Somchit, M. N. H., & Hasan, A. (2012). Honey hydrogel dressing to treat burn wound in rats - a preliminary report. *Pertanika Journal of Tropical Agricultural Science*, *35*(1), 67–74.
78. Mukai, K., Komatsu, E., Yamanishi, M., Hutakuchi, M., Kanzaka, K., Uno, Y., Yamazaki, S., Kato, S., Yamamoto, T., Hattori, M., Nakajima, Y., Urai, T., Asano, K., Murakado, N., Okuwa, M., & Nakatani, T. (2017). Effectiveness of changing the application of Japanese honey to a hydrocolloid dressing in between the inflammatory and proliferative phases on cutaneous wound healing in male mice. *Wounds, 29*(1), 1-9.
79. Mythilypriya, R., Shanthi, P., & Sachdanandam, P. (2007). Analgesic, antipyretic and ulcerogenic properties of an indigenous formulation-Kalpaamruthaa. *Phytotherapy Ressearch*, *21*, 574–578. https://doi.org/10.1002/ptr
80. Naseran, S. N., Mokhtari, M., Abedinzade, M., & Shariati, M. (2018). The effects of *Nigella sativa* hydro-alcoholic extract and honey on lipid profile and indices of insulin resistance in polycystic ovarian syndrome Wistar rat model. *Journal of Research in Medical and Dental Science, 6*(4), 81-87.
81. Noori, S., Kokabi, M., & Hassan, Z. M. (2018). Poly(vinyl alcohol)/chitosan/honey/clay responsive nanocomposite hydrogel wound dressing. *Journal of Applied Polymer Science*, *135*(21), 306-313. https://doi.org/10.1002/app.46311
82. Oršolić, N., Sacases, F., Du Sert, P. P., & Bašić, I. (2007). Antimetastatic ability of honey bee products. *Periodicum Biologorum*, *109*(2), 173–180.
83. Paydar, S., Ziaeian, B., Dehghanian, A., Heidarpour, M., Alavi Moghadam, R., Dalfardi, B., & Karladani, A. H. (2017). A comparison of the effects of topical prolavacid solution (a polyhexamethylene biguanide-based wound cleanser) and Medihoney ointment in a rat model of cutaneous wound. *Advances in Wound Care*, *6*(12), 407–412. https://doi.org/10.1089/wound.2017.0747
84. Peimani, A., & Eslammanesh, T. (2014). Effect of topical application of Medihoney on the healing of the maxillary second molar tooth socket in Wistar rats. *Wounds, 26*(8), E55-E59.
85. Rozaini, M. Z., Zuki, A., Noordin, M., Norimah, Y., & Nazrul, H. (2004). Histological evaluation on burns wound healing treated with Nenas (*Ananas comosus* spp.) and Gelam (*Melaleuca* spp.) honey. *The 11th International Conference of the Association of Institutions for Tropical Veterinary Medicine and 16th Veterinary Association Malaysia Congress, 23-27 August 2004, Petaling Jaya, Malaysia*, 381-383.
86. Rozaini, M. Z., Zuki, A., Noordin, M., Norimah, Y., & Nazrul, H. (2004). Tensile strength evaluation on burns wound healing treated with Nenas (*Ananas comosus* spp.) and Gelam (*Melaleuca* spp.) honey. *The 11th International Conference of the Association of Institutions for Tropical Veterinary Medicine and 16th Veterinary Association Malaysia Congress, 23-27 August 2004, Petaling Jaya, Malaysia*, 338-339.
87. Sahin, A., Turkmen, S., Guzel, N., Mentese, A., Turedi, S., Karahan, S. C., Yulug, E., Demir, S., Aynaci, O., Deger, O., & Gunduz, A. (2018). A Comparison of the effects of

Grayanotoxin-containing honey (Mad honey), normal honey, and propolis on fracture healing. *Medical Principles and Practice*, *27*(2), 99–106. https://doi.org/10.1159/000487552
88. Sarhan, W. A., & Azzazy, H. M. (2017). Apitherapeutics and phage-loaded nanofibers as wound dressings with enhanced wound healing and antibacterial activity. *Nanomedicine*, *12*(17), 2055–2067. https://doi.org/10.2217/nnm-2017-0151
89. Sawazaki, T., Nakajima, Y., Urai, T., Mukai, K., Ohta, M., Kato, I., Kawaguchi, A., Kinoshita, Y., Kumagai, Y., Sakashita, A., & Yamazaki, A. (2018). Efficacy of honeydew honey and blossom honey on full-thickness wound healing in mice. *Wounds, 30*(7), 197-204.
90. Varalakshmi, V., Suganiya, S., & Mala, R. (2015). Fabrication and characterization of hybrid sponge for healing of infectious burn wound. *Recent Patents on Nanotechnology*, *9*(3), 212–221. https://doi.org/10.2174/1872210510999151126112122
91. Yudaniayanti, I. S., Primarizky, H., & Nangoi, L. (2018). The effects of honey (*Apis dorsata*) supplements on increased bone strength in ovariectomized rat as animal model of osteoporosis. *AIP Conference Proceedings*, *1945,* 020004. https://doi.org/10.1063/1.5030226
92. Yuslianti, E. R., Bachtiar, B. M., Suniarti, D. F., Sutjiatmo, A. B., Yuslianti, E. R., Bachtiar, B. M., Suniarti, D. F., & Sutjiatmo, A. B. (2015). Effect of topical Rambutan honey pharmaceutical grade on oral mucosa wound healing based on tissue wound closure and fibroblasts proliferation in vivo. *International Journal of Pharmacology*, *11*(7), 864–869. https://doi.org/10.3923/ijp.2015.864.869
93. Tajik, H., Jalali, F. S. S., Javadi, S., Shahbazi, Y., & Amini, M. (2009). Clinical and microbiological evaluations of efficacy of combination of natural honey and yarrow on repair process of experimental burn wound. *Journal of Animal and Veterinary Advances, 8*(5), 907-922.
94. Ali, A. T. M. M., Al-Swayeh, O. A., Al-Humayyd, M. S., Mustafa, A. A., Al-Rashed, R. S., & Al-Tuwaijiri, A. S. (1997). Natural honey prevents ischemia-reperfusion-induced gastric mucosal lesions and increased vascular permeability in rats. *European Journal of Gastroenterology and Hepatology, 9*, 1101-1107.
95. Gheldof, N. C. M. (1996). Honey as a source of natural antioxidants (unpublished doctoral dissertation). University of Illinois at Urbana-Champaign, United States.
96. Abd Aziz, C. B., Ahmad Suhaimi, S. Q., Hasim, H., Ahmad, A. H., Long, I., & Zakaria, R. (2019). Effects of Tualang honey in modulating nociceptive responses at the spinal cord in offspring of prenatally stressed rats. *Journal of Integrative Medicine*, *17*(1), 66–70. https://doi.org/10.1016/j.joim.2018.12.002
97. Abd Aziz, C. B., Ahmad, R., Mohamed, M., & Wan Yusof, W. N. (2013). The effects of tualang honey intake during prenatal stress on pain responses in the rat offsprings. *European Journal of Integrative Medicine*, *5*(4), 326–331. https://doi.org/10.1016/j.eujim.2013.03.001
98. Abdelaziz, I., Elhabiby, M. I., & Ashour, A. A. (2013). Toxicity of cadmium and protective effect of bee honey, vitamins C and B complex. *Human and Experimental Toxicology*, *32*(4), 362–370. https://doi.org/10.1177/0960327111429136
99. Abdelrahman, M. T., Maina, E. N., & Elshemy, H. A. (2018). Clove (*Syzygium aromaticum*) and honey extracts significantly reduce inflammatory cytokines and liver function enzymes in experimental rats fed on carbon tetrachloride (CCl_4). *Journal of*

Radiation Research and Applied Sciences, *11*(4), 416–422. https://doi.org/10.1016/j.jrras.2018.08.003

100. Abdul Sani, N. F., Belani, L. K., Pui Sin, C., Abdul Rahman, S. N. A., Das, S., Zar Chi, T., Makpol, S., & Yusof, Y. A. M. (2014). Effect of the combination of gelam honey and ginger on oxidative stress and metabolic profile in streptozotocin-induced diabetic sprague-dawley rats. *BioMed Research International*, 160695. https://doi.org/10.1155/2014/160695
101. Abdulmajeed, W. I., Sulieman, H. B., Zubayr, M. O., Imam, A., Amin, A., Biliaminu, S. A., Oyewole, L. A., & Owoyele, B. V. (2015). Honey prevents neurobehavioural deficit and oxidative stress induced by lead acetate exposure in male wistar rats-a preliminary study. *Metabolic Brain Disease*, *31*(1), 37–44. https://doi.org/10.1007/s11011-015-9733-6
102. Adikwu, M. U., & Alozie, B. U. (2007). Application of snail mucin dispersed in detarium gum gel in wound healing. *Scientific Research and Essays*, *2*(6), 195–198.
103. Afroz, R., Tanvir, E. M., Hossain, M. F., Gan, S. H., Parvez, M., Aminul Islam, M., & Khalil, M. I. (2014). Protective effect of Sundarban honey against acetaminophen-induced acute hepatonephrotoxicity in rats. *Evidence-Based Complementary and Alternative Medicine*, *2014,* 143782. https://doi.org/10.1155/2014/143782
104. Afroz, R., Tanvir, E. M., Karim, N., Hossain, M. S., Alam, N., Gan, S. H., & Khalil, M. I. (2016). Sundarban honey confers protection against isoproterenol-induced myocardial infarction in wistar rats. *BioMed Research International*, *2016,* 6437641. https://doi.org/10.1155/2016/6437641
105. Afshari, M. J., Sheikh, N., & Afarideh, H. (2015). PVA/CM-chitosan/honey hydrogels prepared by using the combined technique of irradiation followed by freeze-thawing. *Radiation Physics and Chemistry*, *113*, 28–35. https://doi.org/10.1016/j.radphyschem.2015.04.023
106. Ahmed, S., & Othman, N. H. (2017). The anti-cancer effects of Tualang honey in modulating breast carcinogenesis: An experimental animal study. *BMC Complementary and Alternative Medicine*, *17*(1), 1–11. https://doi.org/10.1186/s12906-017-1721-4
107. Ahmed, S., Sulaiman, S. A., & Othman, N. H. (2017). Oral administration of Tualang and Manuka honeys modulates breast cancer progression in Sprague-Dawley rats model. *Evidence-Based Complementary and Alternative Medicine*, *2017,* 5904361. https://doi.org/10.1155/2017/5904361
108. Ait Abderrahim, L., Taïbi, K., Ait Abderrahim, N., Boussaid, M., Rios-Navarro, C., & Ruiz-Saurí, A. (2019). Euphorbia honey and garlic: biological activity and burn wound recovery. *Burns*, *45*(7), 1695–1706. https://doi.org/10.1016/j.burns.2019.05.002
109. Akbulut, S., Dogan, Z., Baskiran, A., Elbe, H., & Turkoz, Y. (2019). Effect of a honey and arginine-glutamine-hydroxymethylbutyrate mixture on the healing of colon anastomosis in rats immunosuppressed with tacrolimus. *Biotechnic and Histochemistry*, *94*(7), 514–521. https://doi.org/10.1080/10520295.2019.1601257
110. Al Aamri, Z. M., & Ali, B. H. (2017). Does honey have any salutary effect against streptozotocin - induced diabetes in rats? *Journal of Diabetes and Metabolic Disorders*, *16*(1), 1–6. https://doi.org/10.1186/s40200-016-0278-y
111. Al-Malki, A. L., & Sayed, A. A. R. (2013). Bees' honey attenuation of metanil-yellow-induced hepatotoxicity in rats. *Evidence-Based Complementary and Alternative Medicine*, *2013*. https://doi.org/10.1155/2013/614580

112. Al-Rahbi, B., Zakaria, R., Othman, Z., Hassan, A., Mohd Ismail, Z. I., & Muthuraju, S. (2014). Tualang honey supplement improves memory performance and hippocampal morphology in stressed ovariectomized rats. *Acta Histochemica*, *116*(1), 79–88. https://doi.org/10.1016/j.acthis.2013.05.004

113. Al-Rahbi, B., Zakaria, R., Othman, Z., Hassan, A., & Ahmad, A. H. (2014). Enhancement of BDNF concentration and restoration of the hypothalamic-pituitary-adrenal axis accompany reduced depressive-like behaviour in stressed ovariectomised rats treated with either tualang honey or estrogen. *The Scientific World Journal*, *2014*. https://doi.org/10.1155/2014/310821

114. Al-Rahbi, B., Zakaria, R., Othman, Z., Hassan, A., & Ahmad, A. H. (2014). Protective effects of Tualang honey against oxidative stress and anxiety-like behaviour in stressed ovariectomized rats. *International Scholarly Research Notices*, *2014*, 1–10. https://doi.org/10.1155/2014/521065

115. Al-Seeni, M. N., El Rabey, H. A., & Al-Solamy, S. M. (2015). The protective role of bee honey against the toxic effect of melamine in the male rat kidney. *Toxicology and Industrial Health*, *31*(6), 485–493. https://doi.org/10.1177/0748233714551765

116. Al-Wabel, N. A., Mousa, H. M., Omer, O. H., & Abdel-Salam, A. M. (2007). Biological evaluation of synbiotic fermented milk against lead acetate contamination in rats. *Journal of Food, Agriculture and Environment*, *5*(3–4), 169–172.

117. Al-Waili, N. S. (2003). Intravenous and intrapulmonary administration of honey solution to healthy sheep: effects on blood sugar, renal and liver function tests, bone marrow function, lipid profile, and carbon tetrachloride-induced liver injury. *Journal of Medicinal Food*, *6*(3), 231–247. https://doi.org/10.1089/109662003607166652

118. Al-Waili, N. S., Saloom, K. Y., Akmal, M., Al-Waili, F., Al-Waili, T. N., Al-Waili, A. N., & Ali, A. (2006). Honey ameliorates influence of hemorrhage and food restriction on renal and hepatic functions, and hematological and biochemical variables. *International Journal of Food Sciences and Nutrition*, *57*(5–6), 353–362. https://doi.org/10.1080/09637480600802371

119. Al-Waili, N. S., Saloom, K. Y., Al-Waili, T. N., Al-Waili, A. N., Akmal, M., Al-Waili, F. S., & Al-Waili, H. N. (2006). Influence of various diet regimens on deterioration of hepatic function and hematological parameters following carbon tetrachloride: A potential protective role of natural honey. *Natural Product Research*, *20*(13), 1258–1264. https://doi.org/10.1080/14786410600906475

120. Al-Yahya, M., Mothana, R., Al-Said, M., Al-Dosari, M., Al-Musayeib, N., Al-Sohaibani, M., Parvez, M. K., & Rafatullah, S. (2013). Attenuation of CCl_4-induced oxidative stress and hepatonephrotoxicity by Saudi Sidr honey in rats. *Evidence-Based Complementary and Alternative Medicine*, *2013*. https://doi.org/10.1155/2013/569037

121. Alagwu, E. A., Nneli, R. O., Egwurugwu, J. N., & Osim, E. E. (2011). Gastric cytoprotection and honey intake in albino rats. *Nigerian Journal of Physiological Sciences*, *26*(1), 39–42.

122. Aliyu, M., Ibrahim, S., Inuwa, H. M., Sallau, A. B., Abbas, O., Aimola, I. A., Habila, N., & Uche, N. S. (2013). Ameliorative effects of acacia honey against sodium arsenite-induced oxidative stress in some viscera of male wistar albino rats. *Biochemistry Research International*, *2013*. https://doi.org/10.1155/2013/502438

123. Alizadeh, A. M., Sohanaki, H., Khaniki, M., Mohaghgheghi, M. A., Ghmami, G., & Mosavi, M. (2011). The effect of teucrium polium honey on the wound healing and

tensile strength in rat. *Iranian Journal of Basic Medical Sciences*, *14*(6), 499–505. https://doi.org/10.22038/ijbms.2011.5048

124. Almasaudi, S. B., Abbas, A. T., Al-Hindi, R. R., El-Shitany, N. A., Abdel-Dayem, U. A., Ali, S. S., Saleh, R. M., Al Jaouni, S. K., Kamal, M. A., & Harakeh, S. M. (2017). Manuka honey exerts antioxidant and anti-inflammatory activities that promote healing of acetic acid-induced gastric ulcer in rats. *Evidence-Based Complementary and Alternative Medicine*, *2017*, 1–12. https://doi.org/10.1155/2017/5413917

125. Almasaudi, S. B., El-Shitany, N. A., Abbas, A. T., Abdel-Dayem, U. A., Ali, S. S., Al Jaouni, S. K., & Harakeh, S. (2016). Antioxidant, anti-inflammatory, and antiulcer potential of manuka honey against gastric ulcer in rats. *Oxidative Medicine and Cellular Longevity*, *2016*. https://doi.org/10.1155/2016/3643824

126. Amaral, T. Y., Padilha, I. G., Presídio, G. A., Silveira, E. A. A. S. da, Duarte, A. W. F., Barbosa, A. P. F., Bezerra, A. F. de S., & López, A. M. Q. (2017). Antimicrobial and anti-inflammatory activities of *Apis mellifera* honey on the *Helicobacter pylori* infection of Wistar rats gastric mucosa. *Food Science and Technology (Campinas)*, *37*(Suppl. 1), 34–41. https://doi.org/10.1590/1678-457x.31016

127. Andritoiu, C. V., Ochiuz, L., Andritoiu, V., & Popa, M. (2014). Effect of apitherapy formulations against carbon tetrachloride-induced toxicity in Wistar rats after three weeks of treatment. *Molecules*, *19*(9), 13374–13391. https://doi.org/10.3390/molecules190913374

128. Arabmoazzen, S., Sarkaki, A., Saki, G., & Mirshekar, M. A. (2015). Antidiabetic effect of honey feeding in noise induced hyperglycemic rat: involvement of oxidative stress. *Iranian Journal of Basic Medical Sciences*, *18*(8), 745–751. https://doi.org/10.22038/ijbms.2015.4724

129. Arulkumaran, S., Ramprasath, V. R., Shanthi, P., & Sachdanandam, P. (2007). Alteration of DMBA-induced oxidative stress by additive action of a modified indigenous preparation-Kalpaamruthaa. *Chemico-Biological Interactions*, *167*(2), 99–106. https://doi.org/10.1016/j.cbi.2007.01.013

130. Asari, M. A., Zulkaflee, M. H., Sirajudeen, K. N. S., Mohd Yusof, N. A., & Mohd Sairazi, N. S. (2019). Tualang honey and DHA-rich fish oil reduce the production of pro-inflammatory cytokines in the rat brain following exposure to chronic stress. *Journal of Taibah University Medical Sciences*, *14*(4), 317–323. https://doi.org/10.1016/j.jtumed.2019.06.004

131. Atagana, O. S., & Asagba, S. O. (2014). Protective effects of honey against cadmium-induced alteration of some biochemical parameters in rats. *Toxicological and Environmental Chemistry*, *96*(10), 1557–1563. https://doi.org/10.1080/02772248.2015.1027205

132. Aysan, E., Ayar, E., Aren, A., & Cifter, C. (2002). The role of intra-peritoneal honey administration in preventing post-operative peritoneal adhesions. *European Journal of Obstetrics and Gynecology and Reproductive Biology*, *104*(2), 152–155. https://doi.org/10.1016/S0301-2115(02)00070-2

133. Ayyildiz, A., Akgül, T. K., Cebeci, Ö., Nuhoğlu, B., Çaydere, M., Üstün, H., & Germiyanoğlu, C. (2007). Intraurethral honey application for urethral injury: an experimental study. *International Urology and Nephrology*, *39*(3), 815–821. https://doi.org/10.1007/s11255-006-9152-z

134. Azman, K. F., Zakaria, R., Abdul Aziz, C. B., & Othman, Z. (2016). Tualang honey attenuates noise stress-induced memory deficits in aged rats. *Oxidative Medicine and Cellular Longevity*, *2016*. https://doi.org/10.1155/2016/1549158

135. Azman, K. F., Zakaria, R., Othman, Z., & Abdul Aziz, C. B. (2018). Neuroprotective effects of Tualang honey against oxidative stress and memory decline in young and aged rats exposed to noise stress. *Journal of Taibah University for Science*, *12*(3), 273–284. https://doi.org/10.1080/16583655.2018.1465275

136. Aznan, M. I., Khan, O. H., Unar, A. O., Tuan Sharif, S. E., Khan, A. H., Syed, S. H., & Zakaria, A. D. (2016). Effect of Tualang honey on the anastomotic wound healing in large bowel anastomosis in rats-a randomized controlled trial. *BMC Complementary and Alternative Medicine*, *16*(1), 1–7. https://doi.org/10.1186/s12906-016-1003-6

137. B. B. Waykar and Y. A. Alqadhi. (2019). Protective role of honey and royal jelly on cisplatin induced oxidative stress in liver of rat. *International Journal of Pharmaceutical Sciences And Research*, *10*(8), 3898. https://doi.org/10.13040/IJPSR.0975-8232.10(8).3898-04

138. Bashkaran, K., Zunaina, E., Bakiah, S., Sulaiman, S. A., Sirajudeen, K., & Naik, V. (2011). Anti-inflammatory and antioxidant effects of Tualang honey in alkali injury on the eyes of rabbits: experimental animal study. *BMC Complementary and Alternative Medicine*, *11*(1), 90. https://doi.org/10.1186/1472-6882-11-90

139. Bergman, A., Yanai, J., Weiss, J., Bell, D., & David, M. P. (1983). Acceleration of wound healing by topical application of honey. An animal model. *The American Journal of Surgery*, *145*(3), 374–376. https://doi.org/10.1016/0002-9610(83)90204-0

140. Yegani, A. A., Kheirkhah, H. A., Ostovar, A., Koohi, V., & Öztürk, Ö. (2015). Effect of locally produced honey on serum levels of glucose, triglyceride, cholesterol, HDL, VLDL, and LDL in alloxanized diabetic rats. *International Journal of Biosciences (IJB)*, *6*(3), 137–145. https://doi.org/10.12692/ijb/6.3.137-145

141. Bukhari, M. H., Khalil, J., Qamar, S., Qamar, Z., Zahid, M., Ansari, N., & Bakhshi, I. M. (2011). Comparative gastroprotective effects of natural honey, *Nigella sativa* and cimetidine against acetylsalicylic acid induced gastric ulcer in albino rats. *Journal of the College of Physicians and Surgeons Pakistan*, *21*(3), 151–156. https://doi.org/03.2011/JCPSP.151156

142. Cai, M., Shin, B. Y., Kim, D. H., Kim, J. M., Park, S. J., Park, C. S., Won, D. H., Hong, N. D., Kang, D. H., Yutaka, Y., & Ryu, J. H. (2011). Neuroprotective effects of a traditional herbal prescription on transient cerebral global ischemia in gerbils. *Journal of Ethnopharmacology*, *138*(3), 723–730. https://doi.org/http://dx.doi.org/10.1016/j.jep.2011.10.016

143. Celepli, S., Kismet, K., Kaptanoğlu, B., Erel, S., Özer, S., Celepli, P., Kaygusuz, G., Devrim, E., Gencay, Ö., Sorkun, K., Durak, I., & Akkuş, M. A. (2011). The effect of oral honey and pollen on postoperative intraabdominal adhesions. *Turkish Journal of Gastroenterology*, *22*(1), 65–72. https://doi.org/10.4318/tjg.2011.0159

144. Cheng, N., Du, B., Wang, Y., Gao, H., Cao, W., Zheng, J., & Feng, F. (2014). Antioxidant properties of jujube honey and its protective effects against chronic alcohol-induced liver damage in mice. *Food and Function*, *5*(5), 900–908. https://doi.org/10.1039/c3fo60623f

145. Cheng, N., Wu, L., Zheng, J., & Cao, W. (2015). Buckwheat honey attenuates carbon tetrachloride-induced liver and DNA damage in mice. *Evidence-Based Complementary and Alternative Medicine*, *2015*. https://doi.org/10.1155/2015/987385

146. Chepulis, L. M., Starkey, N. J., Waas, J. R., & Molan, P. C. (2009). The effects of long-term honey, sucrose or sugar-free diets on memory and anxiety in rats. *Physiology and Behavior*, *97*(3–4), 359–368. https://doi.org/10.1016/j.physbeh.2009.03.001

147. Choi, C., Kim, W. S., Park, Y. H., Park, S. C., Jang, M. K., & Nah, J. W. (2014). Water-soluble chitosan and herbal honey compound alleviates atopic dermatitis-like lesions in NC/Nga mice. *Journal of Industrial and Engineering Chemistry*, *20*(2), 499–504. https://doi.org/10.1016/j.jiec.2013.05.008

148. Choi, D. S., Kim, S., Lim, Y. M., Gwon, H. J., Park, J. S., Nho, Y. C., & Kwon, J. (2012). Hydrogel incorporated with chestnut honey accelerates wound healing and promotes early HO-1 protein expression in diabetic (*db/db*) mice. *Tissue Engineering and Regenerative Medicine*, *9*(1), 36–42. https://doi.org/10.1007/s13770-012-0036-2

149. Dawane, J. S., Biradar, A., Vaidya, K., Sharma, A., Bhosale, M., & Pandit, V. A. (2018). Burn wound healing potential of honey, sandal wood, calendula and cooling with tap water-A comparative study on wistar rats. *Journal of Clinical and Diagnostic Research*, *12*(10), FC10–FC13. https://doi.org/10.7860/JCDR/2018/36971.12125

150. de Assis, P. O. A., Guerra, G. C. B., Araújo, D. F. de S., de Araújo Júnior, R. F., Machado, T. A. D. G., de Araújo, A. A., de Lima, T. A. S., Garcia, H. E. M., de Andrade, L. de F. L. I., & Queiroga, R. de C. R. do E. (2016). Intestinal anti-inflammatory activity of goat milk and goat yoghurt in the acetic acid model of rat colitis. *International Dairy Journal*, *56*, 45–54. https://doi.org/10.1016/j.idairyj.2015.11.002

151. Dzeufiet, P. D. D., Mogueo, A., Bilanda, D. C., Aboubakar, B. F. O., Tédong, L., Dimo, T., & Kamtchouing, P. (2014). Antihypertensive potential of the aqueous extract which combine leaf of *Persea americana* Mill. (Lauraceae), stems and leaf of *Cymbopogon citratus* (D.C) Stapf. (Poaceae), fruits of *Citrus medical* L. (Rutaceae) as well as honey in ethanol and sucrose experimental model. *BMC Complementary and Alternative Medicine*, *14*(1), 1–12. https://doi.org/10.1186/1472-6882-14-507

152. Ebrahimi, M., Dehghani, F., Farhadian, N., Karimi, M., & Golmohammadzadeh, S. (2017). Investigating the anti-apoptotic effect of sesame oil and honey in a novel nanostructure form for treatment of heart failure. *Nanomedicine Journal*, *4*(4), 245–253. https://doi.org/10.22038/nmj.2017.04.007

153. Ekanem, J. T., Majolagbe, O. R., Sulaiman, F. A., & Muhammad, N. O. (2006). Effects of honey-supplemented diet on the parasitemia and some enzymes of *Trypanosoma brucei*-infected rats. *African Journal of Biotechnology*, *5*(17), 1557–1561. https://doi.org/10.5897/AJB06.289

154. El Denshary, E. S., Al-Gahazali, M. A., Mannaa, F. A., Salem, H. A., Hassan, N. S., & Abdel-Wahhab, M. A. (2012). Dietary honey and ginseng protect against carbon tetrachloride-induced hepatonephrotoxicity in rats. *Experimental and Toxicologic Pathology*, *64*(7–8), 753–760. https://doi.org/10.1016/j.etp.2011.01.012

155. El Rabey, H. A., Al-Seeni, M. N., Al-Sieni, A. I., Al-Hamed, A. M., Zamzami, M. A., & Almutairi, F. M. (2019). Honey attenuates the toxic effects of the low dose of tartrazine in male rats. *Journal of Food Biochemistry*, *43*(4), 1–11. https://doi.org/10.1111/jfbc.12780

156. El Rabey, H. A., Al-Seeni, M. N., & Al-Solamy, S. M. (2013). Bees' honey protects the liver of male rats against melamine toxicity. *BioMed Research International*, *2013*, 14–16. https://doi.org/10.1155/2013/786051

157. El-Aidy, W. K., Ebeid, A. A., Sallam, A. E. R. M., Muhammad, I. E., Abbas, A. T., Kamal, M. A., & Sohrab, S. S. (2015). Evaluation of propolis, honey, and royal jelly in amelioration of peripheral blood leukocytes and lung inflammation in mouse conalbumin-induced asthma model. *Saudi Journal of Biological Sciences*, *22*(6), 780–788. https://doi.org/10.1016/j.sjbs.2014.11.005

158. El-Arab, A. M. E., Girgis, S. M., Hegazy, E. M., & El-Khalek, A. B. A. (2006). Effect of dietary honey on intestinal microflora and toxicity of mycotoxins in mice. *BMC Complementary and Alternative Medicine*, *6*, 1–13. https://doi.org/10.1186/1472-6882-6-6

159. El-Kased, R. F., Amer, R. I., Attia, D., & Elmazar, M. M. (2017). Honey-based hydrogel: In vitro and comparative in vivo evaluation for burn wound healing. *Scientific Reports*, *7*(1), 1–11. https://doi.org/10.1038/s41598-017-08771-8

160. El-Khayat, Z., Ezzat, A. R., Arbid, M. S., Rasheed, W. I., & Elias, T. R. (2009). Potential effects of bee honey and propolis against the toxicity of ochratoxin A in rats. *Macedonian Journal of Medical Sciences*, *2*(4), 311–318. https://doi.org/10.3889/MJMS.1857-5773.2009.0073

161. Emre, A., Akin, M., Isikgonul, I., Yuksel, O., Anadol, A. Z., & Cifter, C. (2009). Comparison of intraperitoneal honey and sodium hyaluronate-carboxymethylcellulose (Seprafilm™) for the prevention of postoperative intra-abdominal adhesions. *Clinics*, *64*(4), 363–368. https://doi.org/10.1590/S1807-59322009000400016

162. Eraslan, G., Kanbur, M., Silici, S., & Karabacak, M. (2010). Beneficial effect of pine honey on trichlorfon induced some biochemical alterations in mice. *Ecotoxicology and Environmental Safety*, *73*(5), 1084–1091. https://doi.org/10.1016/j.ecoenv.2010.02.017

163. Erejuwa, O. O., Nwobodo, N. N., Akpan, J. L., Okorie, U. A., Ezeonu, C. T., Ezeokpo, B. C., Nwadike, K. I., Erhiano, E., Abdul Wahab, M. S., & Sulaiman, S. A. (2016). Nigerian honey ameliorates hyperglycemia and dyslipidemia in alloxan-induced diabetic rats. *Nutrients*, *8*(3), 1–14. https://doi.org/10.3390/nu8030095

164. Erejuwa, O. O., Sulaiman, S. A., Ab Wahab, M. S., Sirajudeen, K. N. S., Salleh, S., & Gurtu, S. (2012). Honey supplementation in spontaneously hypertensive rats elicits antihypertensive effect via amelioration of renal oxidative stress. *Oxidative Medicine and Cellular Longevity*, *2012*, 7–9. https://doi.org/10.1155/2012/374037

165. Erejuwa, O. O., Sulaiman, S. A., Wahab, M. S. A., Sirajudeen, K. N. S., Salleh, M. S. M., & Gurtu, S. (2011). Differential responses to blood pressure and oxidative stress in streptozotocin-induced diabetic wistar-kyoto rats and spontaneously hypertensive rats: effects of antioxidant (honey) treatment. *International Journal of Molecular Sciences*, *12*(3), 1888–1907. https://doi.org/10.3390/ijms12031888

166. Erejuwa, O. O., Sulaiman, S. A., ab Wahab, M. S., Salam, S. K. N., md Salleh, M. S., & Gurtu, S. G. (2011). Comparison of antioxidant effects of honey, glibenclamide, metformin, and their combinations in the kidneys of streptozotocin-induced diabetic rats. *International Journal of Molecular Sciences*, *12*(1), 829–843. https://doi.org/10.3390/ijms12010829

167. Erejuwa, O. O., Sulaiman, S. A., Wahab, M. S. A., Sirajudeen, K. N. S., Salleh, M. S. M., & Gurtu, S. (2011). Glibenclamide or metformin combined with honey improves glycemic control in streptozotocin-induced diabetic rats. *International Journal of Biological Sciences*, *7*(2), 244–252. https://doi.org/10.7150/ijbs.7.244
168. Erguder, B. I., Kilicoglu, S. S., Namuslu, M., Kilicoglu, B., Devrim, E., Kismet, K., & Durak, I. (2008). Honey prevents hepatic damage induced by obstruction of the common bile duct. *World Journal of Gastroenterology*, *14*(23), 3729–3732. https://doi.org/10.3748/wjg.14.3729
169. Ergul, E., & Ergul, S. (2010). The effect of honey on the intestinal anastomotic wound healing in rats with obstructive jaundice. *Bratislava Medical Journal*, *111*(5), 265–270.
170. Eteraf-Oskouei, T., Najafi, M., & Gharehbagheri, A. (2013). Natural honey: A new and potent anti-angiogenic agent in the air-pouch model of inflammation. *Drug Research*, *64*(10), 530–536. https://doi.org/10.1055/s-0033-1363229
171. Eyarefe, D. O., Kuforiji, D. I., Jarikre, T. A., & Emikpe, B. O. (2017). Enhanced electroscalpel incisional wound healing potential of honey in wistar rats. *International Journal of Veterinary Science and Medicine*, *5*(2), 128–134. https://doi.org/10.1016/j.ijvsm.2017.10.002
172. Eyarefe, O. D., Emikpe, B. O., Akinloye, S. O., Alonge, T. O., & Fayemi, O. E. (2012). Effects of honey, glutamine and their combination on canine small bowel epithelial cell proliferation following massive resection. *Nigerian Journal of Physiological Sciences*, *27*(2), 189–193.
173. Eyarefe, O. D., Ologunagba, F. M., & Emikpe, B. O. (2014). Wound healing potential of natural honey in diabetic and non-diabetic wistar rats. *African Journal of Biomedical Research*, *17*(1), 15–21.
174. Fard, S. G., Tan, R. T. R., Mohammed, A. A., Meng, G. Y., Muhamad, S. K. S., Al-Jashamy, K. A., & Mohamed, S. (2011). Wound healing properties of *Eucheuma cottonii* extracts in Sprague-Dawley rats. *Journal of Medicinal Plant Research*, *5*(27), 6373–6380. https://doi.org/10.5897/JMPR10.902
175. Farhan, E. M., Chechan, R. A., & Al-kinani, L. Q. (2017). Investigation of role magnetized water used in supplementary feeding for honeybees to modulate the genotoxic side effects induced by cyclophosphamide in mice bone marrow cells. *Journal of Contemporary Medical Sciences*, *3*(12), 313–318. https://doi.org/10.22317/jcms.12201705
176. Farrokhi, M. R., Vasei, M., Fareghbal, S., & Bakhtazad, A. (2011). Effect of honey on peridural fibrosis formation after laminectomy in rats: a novel experimental study. *Evidence-Based Complementary and Alternative Medicine*, *2011*. https://doi.org/10.1155/2011/504967
177. Farzadinia, P., Jofreh, N., Khatamsaz, S., Movahed, A., Akbarzadeh, S., Mohammadi, M., & Bargahi, A. (2016). Anti-inflammatory and wound healing activities of aloe vera, honey and milk ointment on second-degree burns in rats. *International Journal of Lower Extremity Wounds*, *15*(3), 241–247. https://doi.org/10.1177/1534734616645031
178. Fazalda, A., Quraisiah, A., & Nur Azlina, M. F. (2018). Antiulcer Effect of Honey in nonsteroidal anti-inflammatory drugs induced gastric ulcer model in rats: a

systematic review. *Evidence-Based Complementary and Alternative Medicine*, *2018*. https://doi.org/10.1155/2018/7515692

179. Febriyenti, F., Lucida, H., Almahdy, A., Alfikriyah, I., & Hanif, M. (2019). Wound-healing effect of honey gel and film. *Journal of Pharmacy and Bioallied Sciences*, *11*(2), 176–180. https://doi.org/10.4103/JPBS.JPBS_184_18
180. Fernandez-Cabezudo, M. J., El-Kharrag, R., Torab, F., Bashir, G., George, J. A., El-Taji, H., & al-Ramadi, B. K. (2013). Intravenous administration of manuka honey inhibits tumor growth and improves host survival when used in combination with chemotherapy in a melanoma mouse model. *PLoS ONE*, *8*(2). https://doi.org/10.1371/journal.pone.0055993
181. Fihri, A. F., Al-Waili, N. S., El-Haskoury, R., Bakour, M., Amarti, A., Ansari, M. J., & Lyoussi, B. (2016). Protective effect of Morocco carob honey against lead-induced anemia and hepato-renal toxicity. *Cellular Physiology and Biochemistry*, *39*(1), 115–122. https://doi.org/10.1159/000445610
182. Galal, R. M., Zaki, H. F., El-Nasr, M. M. S., & Agha, A. M. (2012). Potential protective effect of honey against paracetamol-induced hepatotoxicity. *Archives of Iranian Medicine*, *15*(11), 674–680.
183. García, Y., Díaz-Castro, J., López-Aliaga, I., Alférez, M. J. M., Ramos, A., & Campos, M. S. (2013). Bioavailability of Fe, Cu, Zn and antioxidant defence in anemic rat supplemented with a mixture of heme/non-heme Fe. *Journal of Food and Nutrition Research*, *52*(2), 128–138.
184. Gencay, C., Kilicoglu, S. S., Kismet, K., Kilicoglu, B., Erel, S., Muratoglu, S., Sunay, A. E., Erdemli, E., & Akkus, M. A. (2008). Effect of honey on bacterial translocation and intestinal morphology in obstructive jaundice. *World Journal of Gastroenterology*, *14*(21), 3410–3415. https://doi.org/10.3748/wjg.14.3410
185. Ghaderi, R., Afshar, M., Akhbarie, H., & Golalipour, M. J. (2010). Comparison of the efficacy of honey and animal oil in accelerating healing of full thickness wound of mice skin. *International Journal of Morphology*, *28*(1), 193–198. https://doi.org/10.4067/s0717-95022010000100027
186. Gharzouli, K., Balint, G. A., Galfi, M., Rimanoczy, A., & Juhasz, A. (2001). The effect of osmolality changes on gastric mucosal endogenous prostacyclin levels in drug-induced experimental ulcer model of rat. *Experimental and Toxicologic Pathology*, *53*(5), 409–411. https://doi.org/10.1078/0940-2993-00208
187. Gharzouli, K., Gharzouli, A., Amira, S., & Khennouf, S. (2001). Protective effect of mannitol, glucose-fructose-sucrose-maltose mixture, and natural honey hyperosmolar solutions against ethanol-induced gastric mucosal damage in rats. *Experimental and Toxicologic Pathology*, *53*(2–3), 175–180. https://doi.org/10.1078/0940-2993-00175
188. Gharzouli, K., Amira, S., Gharzouli, A., & Khennouf, S. (2002). Gastroprotective effects of honey and glucose-fructose-sucrose-maltose mixture against ethanol-, indomethacin-, and acidified aspirin-induced lesions in the rat. *Experimental and Toxicologic Pathology*, *54*(3), 217–221. https://doi.org/10.1078/0940-2993-00255
189. Gholami, M., Abbaszadeh, A., Khanipour Khayat, Z., Anbari, K., Baharvand, P., & Gharravi, A. M. (2018). Honey improves spermatogenesis and hormone secretion in testicular ischaemia–reperfusion-induced injury in rats. *Andrologia*, *50*(1), 1–6. https://doi.org/10.1111/and.12804

190. Gholami, M., Hemmati, M., Taheri-Ghahfarokhi, A., Hoshyar, R., & Moossavi, M. (2016). Expression of glucokinase, glucose 6-phosphatase, and stress protein in streptozotocin-induced diabetic rats treated with natural honey. *International Journal of Diabetes in Developing Countries*, *36*(1), 125–131. https://doi.org/10.1007/s13410-015-0456-3

191. Gholami, M., Abbaszadeh, A., Baharvand, P., Hasanvand, A., Hasanvand, A., & Gharravi, A. M. (2018). Protective effects of Persian honey, *Apis Mellifera Meda Skorikov* on side effects of chemotherapy and ischemia/reperfusion induced testicular injury. *Journal of Complementary and Integrative Medicine*, *15*(4), 1–7. https://doi.org/10.1515/jcim-2016-0035

192. Gill, R., Poojar, B., Bairy, L. K., & Praveen, K. S. E. (2019). Comparative evaluation of wound healing potential of manuka and acacia honey in diabetic and nondiabetic rats. *Journal of Pharmacy and Bioallied Sciences*, *11*(2), 116–126. https://doi.org/10.4103/JPBS.JPBS_257_18

193. Giusto, G., Vercelli, C., Iussich, S., Audisio, A., Morello, E., Odore, R., & Gandini, M. (2017). A pectin-honey hydrogel prevents postoperative intraperitoneal adhesions in a rat model. *BMC Veterinary Research*, *13*(1), 11–15. https://doi.org/10.1186/s12917-017-0965-z

194. Gunaldi, O., Postalci, L., Gucluhan, G., Tugcu, B., Kizilyildirim, S., Daglioglu, Y.K., Ofluoglu, E., & Emel., E. (2013). Assessment of the antimicrobial effect of manuka honey in the impant-related spinal infections in rats. *Journal of Neurological Sciences, 30*(3), 551-558.

195. Gupta, P., Tripathi, A., Agrawal, T., Narayan, C., Singh, B. M., Kumar, M., & Kumar, A. (2016). Synergistic protective effect of picrorhiza with honey in acetaminophen induced hepatic injury. *Indian Journal of Experimental Biology*, *54*(8), 530–536.

196. Guthrie, H. C., Martin, K. R., Taylor, C., Spear, A. M., Whiting, R., Macildowie, S., Clasper, J. C., & Watts, S. A. (2014). A pre-clinical evaluation of silver, iodine and Manuka honey-based dressings in a model of traumatic extremity wounds contaminated with *Staphylococcus aureus*. *Injury*, *45*(8), 1171–1178. https://doi.org/10.1016/j.injury.2014.05.007

197. Hajizadeh, F., Derakhshan, B., Peimani, A., & Abbasi, Z. (2018). Effect of topical honey on mandibular bone defect healing in rats. *Journal of Contemporary Dental Practice*, *19*(1), 47–51. https://doi.org/10.5005/JP-JOURNALS-10024-2210

198. Hamad, R., Jayakumar, C., Ranganathan, P., Mohamed, R., El-Hamamy, M. M. I., Dessouki, A. A., Ibrahim, A., & Ramesh, G. (2015). Honey feeding protects kidney against cisplatin nephrotoxicity through suppression of inflammation. *Clinical and Experimental Pharmacology and Physiology*, *42*(8), 843–848. https://doi.org/10.1111/1440-1681.12433

199. Hamdy, A. A., Elattal, N. A., Amin, M. A., Ali, A. E., Mansour, N. M., Awad, G. E. A., Farrag, A. R. H., & Esawy, M. A. (2018). *In vivo* assessment of possible probiotic properties of *Bacillus subtilis* and prebiotic properties of levan. *Biocatalysis and Agricultural Biotechnology*, *13*(December 2017), 190–197. https://doi.org/10.1016/j.bcab.2017.12.001

200. Hamzaoglu, I., Saribeyoglu, K., Durak, H., Karahasanoglu, T., Boyrak, I., Altug, T., Sirin, F., & Sariyar, M. (2000). Protective covering of surgical wounds with honey

impedes tumor implantation. *Archives of Surgery*, *135*(12), 1414–1417. https://doi.org/10.1001/archsurg.135.12.1414
201. Haron, M. N., & Mohamed, M. (2016). Effect of honey on the reproductive system of male rat offspring exposed to prenatal restraint stress. *Andrologia*, *48*(5), 525–531. https://doi.org/10.1111/and.12473
202. Haron, M. N., Rahman, W. F. W. A., Sulaiman, S. A., & Mohamed, M. (2014). Tualang honey ameliorates restraint stress-induced impaired pregnancy outcomes in rats. *European Journal of Integrative Medicine*, *6*(6), 657–663. https://doi.org/10.1016/j.eujim.2014.07.001
203. Hashemi, B., Bayat, A., Kazemei, T., & Azarpira, N. (2011). Comparison between topical honey and mafenide acetate in treatment of auricular burn. *American Journal of Otolaryngology - Head and Neck Medicine and Surgery*, *32*(1), 28–31. https://doi.org/10.1016/j.amjoto.2009.07.005
204. Hazrati, M., Mehrabani, D., Japoni, A., Montasery, H., Azarpira, N., Hamidian-Shirazi, A. R., & Tanideh, N. (2010). Effect of honey on healing of *Pseudomonas aeruginosa* infected burn wounds in rat. *Journal of Applied Animal Research*, *37*(2), 161–165. https://doi.org/10.1080/09712119.2010.9707117
205. Hegazi, A. G., Al Guthami, F. M., Al Gethami, A. F., & El Fadaly, H. A. (2017). Beneficial effects of *Capparis Spinosa* honey on the immune response of rats infected with *Toxoplasma Gondii*. *Journal of Pharmacopuncture*, *20*(2), 112–118. https://doi.org/10.3831/KPI.2017.20.015
206. Hemadi, M., Saki, G., Rajabzadeh, A., Khodadadi, A., & Sarkaki, A. (2013). The effects of honey and vitamin e administration on apoptosis in testes of rat exposed to noise stress. *Journal of Human Reproductive Sciences*, *6*(1), 54–58. https://doi.org/10.4103/0974-1208.112383
207. Hilliard, G., DeClue, C. E., Minden-Birkenmaier, B. A., Dunn, A. J., Sell, S. A., & Shornick, L. P. (2019). Preliminary investigation of honey-doped electrospun scaffolds to delay wound closure. *Journal of Biomedical Materials Research - Part B Applied Biomaterials*, *107*(8), 2620–2628. https://doi.org/10.1002/jbm.b.34351
208. Hussein, S. Z., Mohd Yusoff, K., Makpol, S., & Mohd Yusof, Y. A. (2013). Gelam honey attenuates carrageenan-induced rat paw inflammation via NF-κB Pathway. *PLoS ONE*, *8*(8). https://doi.org/10.1371/journal.pone.0072365
209. Hussein, S. Z., Mohd Yusoff, K., Makpol, S., & Mohd Yusof, Y. A. (2012). Gelam honey inhibits the production of proinflammatory, mediators NO, PGE 2, TNF-α, and IL-6 in carrageenan-induced acute paw edema in rats. *Evidence-Based Complementary and Alternative Medicine*, *2012*. https://doi.org/10.1155/2012/109636
210. Ibrahim, A., Eldaim, M. A. A., & Abdel-Daim, M. M. (2016). Nephroprotective effect of bee honey and royal jelly against subchronic cisplatin toxicity in rats. *Cytotechnology*, *68*(4), 1039–1048. https://doi.org/10.1007/s10616-015-9860-2
211. Imada, T., Nakamura, S., Kitamura, N., Shibuya, I., & Tsubota, K. (2014). Oral administration of royal jelly restores tear secretion capacity in rat blink-suppressed dry eye model by modulating lacrimal gland function. *PLoS ONE*, *9*(9), 1–7. https://doi.org/10.1371/journal.pone.0106338
212. Jafari Anarkooli, I., Barzegar Ganji, H., & Pourheidar, M. (2014). The protective effects of insulin and natural honey against hippocampal cell death in streptozotocin-

induced diabetic rats. *Journal of Diabetes Research*, *2014*. https://doi.org/10.1155/2014/491571
213. Jaganathan, S. K., Mondhe, D., Wani, Z. A., & Supriyanto, E. (2014). Evaluation of selected honey and one of its phenolic constituent eugenol against L1210 lymphoid leukemia. *Scientific World Journal*, *2014*, 10–12. https://doi.org/10.1155/2014/912051
214. Jang, M., Lee, M. J., Lee, J. M., Bae, C. S., Kim, S. H., Ryu, J. H., & Cho, I. H. (2014). Oriental medicine Kyung-Ok-Ko prevents and alleviates dehydroepiandrosterone-induced polycystic ovarian syndrome in rats. *PLoS ONE*, *9*(2). https://doi.org/10.1371/journal.pone.0087623
215. Jastrzebska-Stojko, Z., Stojko, R., Rzepecka-Stojko, A., Kabała-Dzik, A., & Stojko, J. (2013). Biological activity of propolis-honey balm in the treatment of experimentally-evoked burn wounds. *Molecules*, *18*(11), 14397–14413. https://doi.org/10.3390/molecules181114397
216. Javadi, S. M. R., Hashemi, M., Mohammadi, Y., MamMohammadi, A., Sharifi, A., & Makarchian, H. R. (2018). Synergistic effect of honey and *nigella sativa* on wound healing in rats. *Acta Cirurgica Brasileira*, *33*(6), 518–523. https://doi.org/10.1590/s0102-865020180060000006
217. Kabała-Dzik, A., Stojko, R., Szaflarska-Stojko, E., Wróblewska-Adamek, I., Stojko, A., Stojko, J., & Stawiarska-Pięta, B. (2004). Influence of honey-balm on the rate of scare formation during experimental burn wound healing in pigs. *Bulletin of the Veterinary Institute in Pulawy*, *48*(3), 311–316.
218. Kadir, E. A., Sulaiman, S. A., Yahya, N. K., & Othman, N. H. (2013). Inhibitory effects of tualang honey on experimental breast cancer in rats: A preliminary study. *Asian Pacific Journal of Cancer Prevention*, *14*(4), 2249–2254. https://doi.org/10.7314/APJCP.2013.14.4.2249
219. Kalantari, N., Ghasemi, M., Bayani, M., & Ghaffari, S. (2016). Effect of honey on mRNA expression of TNF-α, IL-1β and IL-6 following acute toxoplasmosis in mice. *Cytokine*, *88*, 85–90. https://doi.org/10.1016/j.cyto.2016.08.029
220. Kamaruzaman, N. A., Sulaiman, S. A., Kaur, G., & Yahaya, B. (2014). Inhalation of honey reduces airway inflammation and histopathological changes in a rabbit model of ovalbumin-induced chronic asthma. *BMC Complementary and Alternative Medicine*, *14*(Il), 1–11. https://doi.org/10.1186/1472-6882-14-176
221. Kassim, M., Achoui, M., Mansor, M., & Yusoff, K. M. (2010). The inhibitory effects of Gelam honey and its extracts on nitric oxide and prostaglandin E2 in inflammatory tissues. *Fitoterapia*, *81*(8), 1196–1201. https://doi.org/10.1016/j.fitote.2010.07.024
222. Kassim, M., Mansor, M., Al-Abd, N., & Yusoff, K. M. (2012). Gelam honey has a protective effect against lipopolysaccharide (LPS)-induced organ failure. *International Journal of Molecular Sciences*, *13*(5), 6370–6381. https://doi.org/10.3390/ijms13056370
223. Kassim, M., Yusoff, K. M., Ong, G., Sekaran, S., Yusof, M. Y. B. M., & Mansor, M. (2012). Gelam honey inhibits lipopolysaccharide-induced endotoxemia in rats through the induction of heme oxygenase-1 and the inhibition of cytokines, nitric oxide, and high-mobility group protein B1. *Fitoterapia*, *83*(6), 1054–1059. https://doi.org/10.1016/j.fitote.2012.05.008
224. Khalil, M. I., Tanvir, E. M., Afroz, R., Sulaiman, S. A., & Gan, S. H. (2015). Cardioprotective effects of tualang honey: Amelioration of cholesterol and cardiac

enzymes levels. *BioMed Research International*, *2015*. https://doi.org/10.1155/2015/286051

225. Khan, M. A., Shahzadi, T., Malik, S. A., Shahid, M., Ismail, M., Zubair, M., & Iqbal, S. (2019). Pharmacognostic evaluation of turmeric (*Curcuma longa*) extracts in diabetic wound healing. *Journal of Animal and Plant Sciences*, *29*(1), 68–74.
226. Khoo, Y. T., Halim, A. S., Singh, K. K. B., & Mohamad, N. A. (2010). Wound contraction effects and antibacterial properties of Tualang honey on full-thickness burn wounds in rats in comparison to hydrofibre. *BMC Complementary and Alternative Medicine*, *10*, 48. https://doi.org/10.1186/1472-6882-10-48
227. Khorasani, M. Z., Jarrahi, M., & Jarrahi, A. (2016). Combined effect of Iranian propolis and honey on healing of induced incisional wound in rat. *Iioab Journal*, *7*(8), 184–190.
228. Khorshidi, H. R., Kasraianfard, A., Derakhshanfar, A., Rahimi, S., Sharifi, A., Makarchian, H. R., Ghorbanpoor, M., & Javadi, S. M. R. (2017). Evaluation of the effectiveness of sodium hyaluronate, sesame oil, honey, and silver nanoparticles in preventing postoperative surgical adhesion formation. An experimental study. *Acta Cirurgica Brasileira*, *32*(8), 626–632. https://doi.org/10.1590/s0102-865020170080000004
229. Kilicoglu, B., Gencay, C., Kismet, K., Serin Kilicoglu, S., Erguder, I., Erel, S., Sunay, A. E., Erdemli, E., Durak, I., & Akkus, M. A. (2008). The ultrastructural research of liver in experimental obstructive jaundice and effect of honey. *American Journal of Surgery*, *195*(2), 249–256. https://doi.org/10.1016/j.amjsurg.2007.04.011
230. Kim, T.-H., Lee, K. M., Hong, N. D., & Jung, Y.-S. (2016). Anti-platelet and anti-thrombotic effect of a traditional herbal medicine Kyung-Ok-Ko. *Journal of Ethnopharmacology*, *178*, 172–179. https://doi.org/http://dx.doi.org/10.1016/j.jep.2015.11.040
231. Kolawole, T. A., Oyeyemi, W. A., Adigwe, C., Leko, B., Udeh, C., & Dapper, D. V. (2015). Honey attenuates the detrimental effects of nicotine on testicular functions in nicotine treated wistar rats. *Nigerian Journal of Physiological Sciences : Official Publication of the Physiological Society of Nigeria*, *30*(1–2), 11–16.
232. Korkmaz, A., & Kolankaya, D. (2009). Anzer honey prevents N-ethylmaleimide-induced liver damage in rats. *Experimental and Toxicologic Pathology*, *61*(4), 333–337. https://doi.org/10.1016/j.etp.2008.07.005
233. Kotian, S., Bhat, K., Pai, S., Nayak, J., Souza, A., Gourisheti, K., & Padma, D. (2018). The Role of Natural Medicines on Wound Healing: A Biomechanical, Histological, Biochemical and Molecular Study. *Ethiopian Journal of Health Sciences*, *28*(6), 759–770. https://doi.org/10.4314/ejhs.v28i6.11
234. Koval, V. M., Tykhonov, O. I., & Shpychak, O. S. (2017). Study of specific pharmacological activity of standardized composition of bee product substances for treatment of urogenital system. *Zaporozhye Medical Journal*, *0*(5). https://doi.org/10.14739/2310-1210.2017.5.110237
235. Kumar, V., Aneesh, K. A., Kshemada, K., Ajith, K. G. S., Binil, R. S. S., Deora, N., Sanjay, G., Jaleel, A., Muraleedharan, T. S., Anandan, E. M., Mony, R. S., Valiathan, M. S., Santhosh, K. T. R., & Kartha, C. C. (2017). Amalaki rasayana, a traditional Indian drug enhances cardiac mitochondrial and contractile functions and improves cardiac

function in rats with hypertrophy. *Scientific Reports*, *7*(1), 1–17. https://doi.org/10.1038/s41598-017-09225-x
236. Kumari, S., Harjai, K., & Chhibber, S. (2010). Topical treatment of *Klebsiella pneumoniae* B5055 induced burn wound infection in mice using natural products. *Journal of Infection in Developing Countries*, *4*(6), 367–377. https://doi.org/10.3855/jidc.312
237. Kundu, S., Biswas, T. K., Das, P., Kumar, S., & Kumar De, D. (2005). Turmeric (*Curcuma longa*) rhizome paste and honey show similar wound healing potential: A preclinical study in rabbits. *International Journal of Lower Extremity Wounds*, *4*(4), 205–213. https://doi.org/10.1177/1534734605281674
238. Küpeli Akkol, E., Orhan, D. D., Gürubüz, I., & Yesilada, E. (2010). *In vivo* activity assessment of a “honey-bee pollen mix” formulation. *Pharmaceutical Biology*, *48*(3), 253–259. https://doi.org/10.3109/13880200903085482
239. El-Kutry, M.S. (2015). Ginger and honeybee modulates MTX-induced oxidative stress in kidney of rats. *European Journal of Medicinal Plants*, *5*(1), 53–65. https://doi.org/10.9734/ejmp/2015/11837
240. Lee, M. J., Jang, M., Bae, C. S., Park, K. S., Kim, H. J., Lee, S., Lee, S. W., Kim, Y. O., & Cho, I. H. (2016). Effects of oriental medicine Kyung-Ok-Ko on uterine abnormality in hyperandrogenized rats. *Rejuvenation Research*, *19*(6), 456–466. https://doi.org/10.1089/rej.2015.1787
241. Leong, A. G., Herst, P. M., & Harper, J. L. (2012). Indigenous New Zealand honeys exhibit multiple anti-inflammatory activities. *Innate Immunity*, *18*(3), 459–466. https://doi.org/10.1177/1753425911422263
242. Li, S., Liu, D., Liu, P., Fu, Z., Sun, M., Zhang, Y., & Hu, H. (2017). Comparison of attenuating renal ischemia/reperfusion injury effects of raw and honey wine-processed *Herba Siegesbeckiae*. *International Journal of Clinical and Experimental Medicine*, *10*(1), 524–531.
243. Maameri, Z., Beroual, K., Djerrou, Z., Habibatni, S., Benlaksira, B., Serakta, M., Mansour-Djaalab, H., Kahlouche-Riachi, F., Bachtarzi, K., & Pacha, Y. H. (2012). Preliminary study to assess cicatrizing activity of honey and *Pistacia lentiscus* fatty oil mixture on experimental burns in rabbits. *International Journal of Medicinal and Aromatic Plants*, *2*(3), 476–480.
244. Maghraby, A. S., & Hassan, S. A. (2005). Effect of antioxidative properties of honey on *Schistosoma mansoni*-infected mice. *Polish Journal of Food and Nutrition Sciences*, *14*(3), 323–326.
245. Mahesh, A., Shaheetha, J., Thangadurai, D., & Rao, D. M. (2009). Protective effect of Indian honey on acetaminophen induced oxidative stress and liver toxicity in rat. *Biologia*, *64*(6), 1225–1231. https://doi.org/10.2478/s11756-009-0205-5
246. Mahgoub, A. A., el-Medany, A. H., Hagar, H. H., & Sabah, D. M. (2002). Protective effect of natural honey against acetic acid-induced colitis in rats. *Tropical Gastroenterology : Official Journal of the Digestive Diseases Foundation*, *23*(2), 82–87.
247. Jaganathan, S. K., Mondhe, D., Wani, Z. A., Pal, H. C., & Mandal, M. (2010). Effect of honey and eugenol on ehrlich ascites and solid carcinoma. *Journal of Biomedicine and Biotechnology*, *2010*(1), 1–6. https://doi.org/10.1155/2010/989163
248. Medhi, B., Prakasha, A., Avti, K., Saikia, U. N., Pandhia, P., & Khanduja, K. L. (2008). Effect of manuka honey and sulfasalazine in combination to promote antioxidant

defense system in experimentally induced ulcerative colitis model in rats. *Indian Journal of Experimental Biology*, *46*(8), 583–590.

249. Meskini, M., & Esmaeili, D. (2018). The study of formulated Zoush ointment against wound infection and gene expression of virulence factors *Pseudomonas aeruginosa*. *BMC Complementary and Alternative Medicine*, *18*(1), 1–10. https://doi.org/10.1186/s12906-018-2251-4

250. Meteoglu, I., Kavas, N. C., Saricaoglu, M., Ilkaya, F., Guzel, H., Alici, O., & Öztürk, F. (2015). Chestnut honey and sherbet enhance the healing of burn wounds in rat model. *Clinical and Investigative Medicine*, *38*(4), E267–E273.

251. Mirzaei, B., Etemadian, S., Goli, H. R., Bahonar, S., Gholami, S. A., Karami, P., Farhadi, M., & Tavakoli, R. (2018). Construction and analysis of alginate-based honey hydrogel as an ointment to heal of rat burn wound related infections. *International Journal of Burns and Trauma*, *8*(4), 88–97.

252. Mobarok Ali, A. T. M. (2003). Prevention of ammonia-induced gastric lesions in rats by natural honey. *Journal of Nutritional and Environmental Medicine*, *13*(4), 239–246. https://doi.org/10.1080/13590840310001649899

253. Mohamad Zaid, S. S., Sulaiman, S. A., Othman, N. H., Soelaiman, I. N., Shuid, A. N., Mohamad, N., & Muhamad, N. (2012). Protective effects of Tualang honey on bone structure in experimental postmenopausal rats. *Clinics*, *67*(7), 779–784. https://doi.org/10.6061/clinics/2012(07)13

254. Mohamed, M., Sulaiman, S. A., & Jaafar, H. (2012). Histological changes in male accessory reproductive organs in rats exposed to cigarette smoke and the protective effect of honey supplementation. *African Journal of Traditional, Complementary and Alternative Medicines*, *9*(3), 329–335. https://doi.org/10.4314/ajtcam.v9i3.5

255. Mohamed, M., Sulaiman, S. A., & Sirajudeen, K. N. S. (2013). Protective effect of honey against cigarette smoke induced-impaired sexual behavior and fertility of male rats. *Toxicology and Industrial Health*, *29*(3), 264–271. https://doi.org/10.1177/0748233711432568

256. Mohammadimanesh, A., Mozaffari-Khosravi, H., Vahidiniya, A. A., Doaei, S., Salehi, I., & Fayyaz, N. (2016). The comparative effect of different types of honey on levels of glucose, fructosamine and insulin in Streptozocin-induced diabetes in Wistar rats. *South Asian Journal of Experimental Biology*, *6*(1), 39–44.

257. Mohd Sairazi, N. S., Sirajudeen, K. N. S., Asari, M. A., Mummedy, S., Muzaimi, M., & Sulaiman, S. A. (2017). Effect of tualang honey against KA-induced oxidative stress and neurodegeneration in the cortex of rats. *BMC Complementary and Alternative Medicine*, *17*(1), 1–12. https://doi.org/10.1186/s12906-016-1534-x

258. Mohd Sairazi, N. S., Sirajudeen, K. N. S., Muzaimi, M., Mummedy, S., Asari, M. A., & Sulaiman, S. A. (2018). Tualang honey reduced neuroinflammation and caspase-3 activity in rat brain after kainic acid-induced status epilepticus. *Evidence-Based Complementary and Alternative Medicine*, *2018*. https://doi.org/10.1155/2018/7287820

259. Mohd Zohdi, R., Abu Bakar Zakaria, Z., Yusof, N., Mohamed Mustapha, N., & Abdullah, M. N. H. (2012). Gelam (*Melaleuca* spp.) honey-based hydrogel as burn wound dressing. *Evidence-Based Complementary and Alternative Medicine*, *2012*. https://doi.org/10.1155/2012/843025

260. Momin, M., Kurhade, S., Khanekar, P., & Mhatre, S. (2016). Novel biodegradable hydrogel sponge containing curcumin and honey for wound healing. *Journal of Wound Care*, *25*(6), 364–372. https://doi.org/10.12968/jowc.2016.25.6.364
261. Mosavat, M., Ooi, F. K., & Mohamed, M. (2014). Effects of honey supplementation combined with different jumping exercise intensities on bone mass, serum bone metabolism markers and gonadotropins in female rats. *BMC Complementary and Alternative Medicine*, *14*(1), 1–8. https://doi.org/10.1186/1472-6882-14-126
262. Muhammad, A., Odunola, O. A., Gbadegesin, M. A., Adegoke, A. M., Olugbami, J. O., & Uche, N. S. (2015). Modulatory role of Acacia honey from north-west Nigeria on sodium arsenite-induced clastogenicity and oxidative stress in male Wistar rats. *Natural Product Research*, *29*(4), 321–326. https://doi.org/10.1080/14786419.2014.940945
263. Mukai, K., Koike, M., Nakamura, S., Kawaguchi, Y., Katagiri, F., Nojiri, S., Yamada, Y., Miyajima, E., Matsumoto, M., Komatsu, E., Nakajima, Y., Urai, T., Murakado, N., & Nakatani, T. (2015). Evaluation of the effects of a combination of Japanese honey and hydrocolloid dressing on cutaneous wound healing in male mice. *Evidence-Based Complementary and Alternative Medicine*, *2015*. https://doi.org/10.1155/2015/910605
264. Mythilypriya, R., Shanthi, P., & Sachdanandam, P. (2009). Ameliorating effect of Kalpaamruthaa, a Siddha preparation in adjuvant induced arthritis in rats with reference to changes in proinflammatory cytokines and acute phase proteins. *Chemico-Biological Interactions*, *179*(2–3), 335–343. https://doi.org/10.1016/j.cbi.2009.01.001
265. Mythilypriya, R., Shanthi, P., & Sachdanandam, P. (2008). Efficacy of Siddha formulation Kalpaamruthaa in ameliorating joint destruction in rheumatoid arthritis in rats. *Chemico-Biological Interactions*, *176*(2–3), 243–251. https://doi.org/10.1016/j.cbi.2008.07.008
266. Mythilypriya, R., Shanthi, P., & Sachdanandam, P. (2008). Salubrious effect of Kalpaamruthaa, a modified indigenous preparation in adjuvant-induced arthritis in rats-A biochemical approach. *Chemico-Biological Interactions*, *173*(2), 148–158. https://doi.org/10.1016/j.cbi.2008.02.007
267. Mythilypriya, R., Shanthi, P., & Sachdanandam, P. (2007). Restorative and synergistic efficacy of Kalpaamruthaa, a modified Siddha preparation, on an altered antioxidant status in adjuvant induced arthritic rat model. *Chemico-Biological Interactions*, *168*(3), 193–202. https://doi.org/10.1016/j.cbi.2007.04.005
268. Najafi, M., Shaseb, E., Ghaffary, S., Fakhrju, A., & Oskouei, T. E. (2011). Effects of chronic oral administration of natural honey on ischemia/reperfusion-induced arrhythmias in isolated rat heart. *Iranian Journal of Basic Medical Sciences*, *14*(1), 75–81. https://doi.org/10.22038/ijbms.2011.4967
269. Nakajima, Y., Mukai, K., Nasruddin, Komatsu, E., Iuchi, T., Kitayama, Y., Sugama, J., & Nakatani, T. (2013). Evaluation of the effects of honey on acute-phase deep burn wounds. *Evidence-Based Complementary and Alternative Medicine*, *2013*. https://doi.org/10.1155/2013/784959
270. Nakajima, Y., Nakano, Y., Fuwano, S., Hayashi, N., Hiratoko, Y., Kinoshita, A., Miyahara, M., Mochizuki, T., Nishino, K., Tsuruhara, Y., Yokokawa, Y., Iuchi, T., Kon, Y., Mukai, K., Kitayama, Y., Murakado, N., Okuwa, M., & Nakatani, T. (2013). Effects of three types of Japanese honey on full-thickness wound in mice. *Evidence-Based*

Complementary and Alternative Medicine, *2013*, 1–11. https://doi.org/10.1155/2013/504537

271. Nandhakumar, E., Purushothaman, A., & Sachdanandam, P. (2014). Protective effect of Shemamruthaa on lipids anomalies in 7,12-dimethylbenz[a]anthracene (DMBA)-induced mammary carcinoma-bearing rats. *Medicinal Chemistry Research*, *23*(7), 3491–3502. https://doi.org/10.1007/s00044-014-0921-4
272. Nasrolahi, O., Khaneshi, F., Rahmani, F., & Razi, M. (2013). Honey and metformin ameliorated diabetes-induced damages in testes of rat; correlation with hormonal changes. *Iranian Journal of Reproductive Medicine*, *11*(12), 1013–1020. http://www.ncbi.nlm.nih.gov/pubmed/24639728%0Ahttp://www.pubmedcentral.nih.gov/articlerender.fcgi?artid=PMC3941405
273. Nasruddin, Putri, I. K., Kamal, S., Esti Rahayu, H. S., Lutfiyati, H., Pribadi, P., Kusuma, T. M., Muhlisin, Z., Nur, M., Nurani, L. H., Santosa, B., Ishijima, T., & Nakatani, T. (2017). Evaluation the effectiveness of combinative treatment of cold plasma jet, Indonesian honey, and micro-well dressing to accelerate wound healing. *Clinical Plasma Medicine*, *5–6*(March), 14–25. https://doi.org/10.1016/j.cpme.2017.03.001
274. Nasuti, C., Gabbianelli, R., Falcioni, G., & Cantalamessa, F. (2006). Antioxidative and gastroprotective activities of anti-inflammatory formulations derived from chestnut honey in rats. *Nutrition Research*, *26*(3), 130–137. https://doi.org/10.1016/j.nutres.2006.02.007
275. Neamatallah, T., El-Shitany, N. A., Abbas, A. T., Ali, S. S., & Eid, B. G. (2018). Honey protects against cisplatin-induced hepatic and renal toxicity through inhibition of NF-κB-mediated COX-2 expression and the oxidative stress dependent BAX/Bcl-2/caspase-3 apoptotic pathway. *Food and Function*, *9*(7), 3743–3754. https://doi.org/10.1039/c8fo00653a
276. Negahi, A. R., Hosseinpour, P., Vaziri, M., Vaseghi, H., Darvish, P., Bouzari, B., & Mousavie, S. H. (2019). Comparison of honey versus polylactide anti-adhesion barrier on peritoneal adhesion and healing of colon anastomosis in rabbits. *Open Access Macedonian Journal of Medical Sciences*, *7*(10), 1597–1601. https://doi.org/10.3889/oamjms.2019.284
277. Nejabat, M., Astaneh, A., Eghtedari, M., Mosallaei, M., Ashraf, M. J., & Mehrabani, D. (2009). Effect of honey in *Pseudomonas aeruginosa* induced stromal keratitis in rabbits. *Journal of Applied Animal Research*, *35*(2), 101–104. https://doi.org/10.1080/09712119.2009.9706996
278. Nho, Y. C., Park, J. S., & Lim, Y. M. (2014). Preparation of hydrogel by radiation for the healing of diabetic ulcer. *Radiation Physics and Chemistry*, *94*(1), 176–180. https://doi.org/10.1016/j.radphyschem.2013.07.021
279. Nikaein, D., Khosravi, A. R., Moosavi, Z., Shokri, H., Erfanmanesh, A., Ghorbani-Choboghlo, H., & Bagheri, H. (2014). Effect of honey as an immunomodulator against invasive aspergillosis in BALB/c mice. *Journal of Apicultural Research*, *53*(1), 84–90. https://doi.org/10.3896/IBRA.1.53.1.08
280. Nooh, H. Z., & Nour-Eldien, N. M. (2016). The dual anti-inflammatory and antioxidant activities of natural honey promote cell proliferation and neural regeneration in a rat model of colitis. *Acta Histochemica*, *118*(6), 588–595. https://doi.org/10.1016/j.acthis.2016.06.006

281. Noorhafiza, R., Majid, A. M., & Hashida, N. H. (2013). Testosterone level and histological features of tualang honey and nicotine treated male rats. *Biomedical Research (India)*, *24*(3), 383–388.
282. Oguz, S., Salt, O., Ibis, A. C., Gurcan, S., Albayrak, D., Yalta, T., Sagiroglu, T., & Erenoglu, C. (2018). Combined effectiveness of honey and immunonutrition on bacterial translocation secondary to obstructive jaundice in rats: Experimental study. *Medical Science Monitor*, *24*, 3374–3381. https://doi.org/10.12659/MSM.907977
283. Omotayo, E. O., Gurtu, S., Sulaiman, S. A., Wahab, M. S. A., Sirajudeen, K. N. S., & Salleh, M. S. M. (2010). Hypoglycemic and antioxidant effects of honey supplementation in streptozotocin-induced diabetic rats. *International Journal for Vitamin and Nutrition Research*, *80*(1), 74–82. https://doi.org/10.1024/0300-9831/a000008
284. Orsolic, N., Knezevic, A., Sver, L., Terzic, S., Hackenberger, B. K., & Basic, I. (2003). Influence of honey bee products on transplantable murine tumours. *Veterinary and Comparative Oncology*, *1*(4), 216–226. https://doi.org/10.1111/j.1476-5810.2003.00029.x
285. Oršolić, N., Jazvinšćak Jembrek, M., & Terzić, S. (2017). Honey and quercetin reduce ochratoxin A-induced DNA damage in the liver and the kidney through the modulation of intestinal microflora. *Food and Agricultural Immunology*, *28*(5), 812–833. https://doi.org/10.1080/09540105.2017.1313819
286. Oršolić, N., Terzić, S., Šver, L., & Bašić, I. (2005). Honey-bee products in prevention and/or therapy of murine transplantable tumours. *Journal of the Science of Food and Agriculture*, *85*(3), 363–370. https://doi.org/10.1002/jsfa.2041
287. Ota, M., Ishiuchi, K., Xu, X., Minami, M., Nagachi, Y., Yagi-Utsumi, M., Tabuchi, Y., Cai, S. Q., & Makino, T. (2019). The immunostimulatory effects and chemical characteristics of heated honey. *Journal of Ethnopharmacology*, *228*(April 2018), 11–17. https://doi.org/10.1016/j.jep.2018.09.019
288. Owoyele, B. V., Adenekan, O. T., & Soladoye, A. O. (2011). Effects of honey on inflammation and nitric oxide production in Wistar rats. *Journal of Chinese Integrative Medicine*, *9*(4), 447–452. https://doi.org/10.3736/jcim20110415
289. Owoyele, B. V., Oladejo, R. O., Ajomale, K., Ahmed, R. O., & Mustapha, A. (2014). Analgesic and anti-inflammatory effects of honey: The involvement of autonomic receptors. *Metabolic Brain Disease*, *29*(1), 167–173. https://doi.org/10.1007/s11011-013-9458-3
290. Paramasivan, S., Drilling, A. J., Jardeleza, C., Jervis-Bardy, J., Vreugde, S., & Wormald, P. J. (2014). Methylglyoxal-augmented manuka honey as a topical anti-Staphylococcus aureus biofilm agent: safety and efficacy in an in vivo model. *International Forum of Allergy and Rhinology*, *4*(3), 187–195. https://doi.org/10.1002/alr.21264
291. Park, E., Long, S. A., Seth, A. K., Geringer, M., Xu, W., Chavez-Munoz, C., Leung, K., Hong, S. J., Galiano, R. D., & Mustoe, T. A. (2016). The use of desiccation to treat *Staphylococcus aureus* biofilm-infected wounds. *Wound Repair and Regeneration*, *24*(2), 394–401. https://doi.org/10.1111/wrr.12379
292. Park, J. S., An, S. J., Jeong, S. I., Gwon, H. J., Lim, Y. M., & Nho, Y. C. (2017). Chestnut honey impregnated carboxymethyl cellulose hydrogel for diabetic ulcer healing. *Polymers*, *9*(7). https://doi.org/10.3390/polym9070248

293. Paydar, S., Akrami, M., Dehghanian, A., Alavi Moghadam, R., Heidarpour, M., Bahari Khoob, A., & Dalfardi, B. (2016). A comparison of the effects of alpha and medical-grade honey ointments on cutaneous wound healing in rats. *Journal of Pharmaceutics*, *2016*, 1–6. https://doi.org/10.1155/2016/9613908

294. Pourali, P., & Yahyaei, B. (2019). The healing property of a bioactive wound dressing prepared by the combination of bacterial cellulose (BC) and *Zingiber officinale* root aqueous extract in rats. *3 Biotech*, *9*(2), 1–9. https://doi.org/10.1007/s13205-019-1588-9

295. Prasetyo, R. H., & Hestianah, E. P. (2017). Honey can repairing damage of liver tissue due to protein energy malnutrition through induction of endogenous stem cells. *Veterinary World*, *10*(6), 711–715. https://doi.org/10.14202/vetworld.2017.711-715

296. Punitan, R., Sulaiman, S. A., Hasan, H. B., & Shatriah, I. (2019). Clinical and antibacterial effects of Tualang honey on *Pseudomonas*-induced keratitis in rabbit eyes. *Cureus*, *11*(3). https://doi.org/10.7759/cureus.4332

297. Rabiul Islam, M., Reazul Islam, M., Anisuzzaman, M. D., & Hossain, S. J. (2019). Antidiarrheal, analgesic, and anthelmintic activities of honeys in the Sundarbans mangrove forest, Bangladesh. *Preventive Nutrition and Food Science*, *24*(1), 49–55. https://doi.org/10.3746/pnf.2019.24.1.49

298. Rahimi, V. B., Shirazinia, R., Fereydouni, N., Zamani, P., Darroudi, S., Sahebkar, A. H., & Askari, V. R. (2017). Comparison of honey and dextrose solution on post-operative peritoneal adhesion in rat model. *Biomedicine and Pharmacotherapy*, *92*, 849–855. https://doi.org/10.1016/j.biopha.2017.05.114

299. Rajabzadeh, A., Sagha, M., Gholami, M. R., & Hemmati, R. (2015). Honey and vitamin E restore the plasma level of gonadal hormones and improve the fertilization capacity in noise-stressed rats. *Crescent Journal of Medical and Biological Sciences*, *2*(2), 64–68.

300. Rezaei, N., Eftekhari, M. H., Tanideh, N., Mokhtari, M., & Bagheri, Z. (2018). Protective effects of honey and *Spirulina platensis* on acetic acid-induced ulcerative colitis in rats. *Iranian Red Crescent Medical Journal*, *20*(4), 1–11. https://doi.org/10.5812/ircmj.62517

301. Romero-Silva, S., Angel Martinez R, M., P. Romero-Romero, L., Rodriguez, O., Gerardo Salas G, C., Morel, N., Javier Lopez-Munoz, F., Angel Lima-Mendoza, L., & Bravo, G. (2010). Effects of honey against the accumulation of adipose tissue and the increased blood pressure on carbohydrate-induced obesity in rat. *Letters in Drug Design & Discovery*, *8*(1), 69–75. https://doi.org/10.2174/157018011793663912

302. Roosdiana, A., Permata, F. S., & Graf, P. S. (2019). The Sumbawa forest honey as preventive agent for organ damaged caused by environmental pollution. *IOP Conference Series: Earth and Environmental Science*, *239*(1). https://doi.org/10.1088/1755-1315/239/1/012031

303. Saber, A. (2010). Effect of honey versus intergel in intraperitoneal adhesion prevention and colonic anastomotic healing: A randomized controlled study in rats. *International Journal of Surgery*, *8*(2), 121–127. https://doi.org/10.1016/j.ijsu.2009.11.010

304. Sakhavar, N., & Khadem, N. (2008). Comparative study of therapeutic effects of honey and povidone iodine in surgical wound healing in rabbit. *Shiraz E Medical Journal*, *9*(4), 182–187.

305. Samat, S., Kanyan Enchang, F., Nor Hussein, F., & Wan Ismail, W. I. (2017). Four-week consumption of Malaysian honey reduces excess weight gain and improves obesity-related parameters in high fat diet induced obese rats. *Evidence-Based Complementary and Alternative Medicine*, *2017*. https://doi.org/10.1155/2017/1342150
306. Saral, Ö., Yildiz, O., Aliyazicioğlu, R., Yuluğ, E., Canpolat, S., Öztürk, F., & Kolayli, S. (2016). Apitherapy products enhance the recovery of CCL_4-induced hepatic damages in rats. *Turkish Journal of Medical Sciences*, *46*(1), 194–202. https://doi.org/10.3906/sag-1411-35
307. Sarhan, W. A., Azzazy, H. M. E., & El-Sherbiny, I. M. (2016). Honey/Chitosan nanofiber wound dressing enriched with *Allium sativum* and *Cleome droserifolia*: enhanced antimicrobial and wound healing activity. *ACS Applied Materials and Interfaces*, *8*(10), 6379–6390. https://doi.org/10.1021/acsami.6b00739
308. Saxena, A. K., Phyu, H. P., Al-Ani, I. M., & Talib, N. A. (2014). Potential protective effect of honey against chronic cerebral hypoperfusion-induced neurodegeneration in rats. *Journal of the Anatomical Society of India*, *63*(2), 151–155. https://doi.org/10.1016/j.jasi.2014.11.003
309. Sazegar, G., Hosseini, S. R. A., & Behravan, E. (2011). The effects of supplemental zinc and honey on wound healing in rats. *Iranian Journal of Basic Medical Sciences*, *14*(4), 391–398. https://doi.org/10.22038/ijbms.2011.5029
310. Schencke, C., Sandoval, C., Vásquez, B., & Sol, M. (2018). Quantitative analysis of dermal scars in deep skin burns treated with Ulmo honey supplemented with ascorbic acid. *International Journal of Clinical and Experimental Medicine*, *11*(3), 2422–2429.
311. Schencke, C., Vasconcellos, A., Sandoval, C., Torres, P., Acevedo, F., & del Sol, M. (2016). Morphometric evaluation of wound healing in burns treated with Ulmo (*Eucryphia cordifolia*) honey alone and supplemented with ascorbic acid in guinea pig (*Cavia porcellus*). *Burns & Trauma*, *4*, 1–9. https://doi.org/10.1186/s41038-016-0050-z
312. Shamaki, B. U., Yusuf, A., Balla, H. J., Halima, I. G., Sherifat, O. B., Abdulrahman, F. I., Ogbe, A. O., & Sandabe, U. K. (2014). Evaluation of chemical composition and the comparative wound healing effect of natural honey and olive oil in rabbits. *Infinity. Communications in Applied Sciences*, *2*(2), 149–169.
313. Shamshuddin, N. S. S., & Mohd Zohdi, R. (2018). Gelam honey attenuates ovalbumin-induced airway inflammation in a mice model of allergic asthma. *Journal of Traditional and Complementary Medicine*, *8*(1), 39–45. https://doi.org/10.1016/j.jtcme.2016.08.009
314. Shati, A. A., Elsaid, F. G., & Hafez, E. E. (2011). Biochemical and molecular aspects of aluminium chloride-induced neurotoxicity in mice and the protective role of *Crocus sativus* L. extraction and honey syrup. *Neuroscience*, *175*, 66–74. https://doi.org/10.1016/j.neuroscience.2010.11.043
315. Shi, P., Chen, B., Chen, C., Xu, J., Shen, Z., Miao, X., & Yao, H. (2015). Honey reduces blood alcohol concentration but not affects the level of serum MDA and GSH-Px activity in intoxicated male mice models. *BMC Complementary and Alternative Medicine*, *15*(1), 1–6. https://doi.org/10.1186/s12906-015-0766-5
316. Singh, S., Gupta, A., & Gupta, B. (2018). Scar free healing mediated by the release of aloe vera and manuka honey from dextran bionanocomposite wound dressings. *International Journal of Biological Macromolecules*, *120*, 1581–1590. https://doi.org/10.1016/j.ijbiomac.2018.09.124

317. Sohanur, A. M., & Munira S, R. M. (2015). Effects of honey supplementation on hepatic and cardiovascular disease(CVD) marker in streptozotocin-induced diabetic rats. *Journal of Diabetes & Metabolism*, *06*(09). https://doi.org/10.4172/2155-6156.1000592

318. Sukur, S. M., Halim, A. S., & Singh, K. K. B. (2011). Evaluations of bacterial contaminated full thickness burn wound healing in Sprague Dawley rats treated with Tualang honey. *Indian Journal of Plastic Surgery*, *44*(1), 112–117. https://doi.org/10.4103/0970-0358.81459

319. Swellam, T., Miyanaga, N., Onozawa, M., Hattori, K., Kawai, K., Shimazui, T., & Akaza, H. (2003). Antineoplastic activity of honey in an experimental bladder cancer implantation model: in vivo and in vitro studies. *International Journal of Urology*, *10*(4), 213–219. https://doi.org/10.1046/j.0919-8172.2003.00602.x

320. Tahir, H. M., Rakha, A., Mukhtar, M. K., Yaqoob, R., Samiullah, K., & Ahsan, M. M. (2017). Evaluation of wound healing potential of spider silk using mice model. *JAPS, Journal of Animal and Plant Sciences*, *27*(6), 1896–1902.

321. Fukuda, M., Kobayashi, K., Hirono, Y., Miyagawa, M., Ishida, T., Ejiogu, E. C., Sawai, M., Pinkerton, K. E., & Takeuchi, M. (2011). Jungle honey enhances immune function and antitumor activity. *Evidence-Based Complementary and Alternative Medicine*, *2011*. https://doi.org/10.1093/ecam/nen086

322. Takzaree, N., Hassanzadeh, G., Rouini, M. R., Manayi, A., Hadjiakhondi, A., & Zolbin, M. M. (2017). Evaluation of the effects of local application of Thyme honey in open cutaneous wound healing. *Iranian Journal of Public Health*, *46*(4), 545–551.

323. Tan, M. K., Hasan Adli, D. S., Tumiran, M. A., Abdulla, M. A., & Yusoff, K. M. (2012). The efficacy of Gelam honey dressing towards excisional wound healing. *Evidence-Based Complementary and Alternative Medicine*, *2012*. https://doi.org/10.1155/2012/805932

324. Tang, S. P., Kuttulebbai Nainamohamed Salam, S., Jaafar, H., Gan, S. H., Muzaimi, M., & Sulaiman, S. A. (2017). Tualang honey protects the rat midbrain and lung against repeated paraquat exposure. *Oxidative Medicine and Cellular Longevity*, *2017*. https://doi.org/10.1155/2017/4605782

325. Tanriverdi, O. (2018). Morphometric analysis of the effects of manuka honey on vasospastic femoral arteries in rats: an experimental study. *The Medical Bulletin of Sisli Hospital*, *52*(4), 268–273. https://doi.org/10.14744/semb.2018.35761

326. Tanvir, E. M., Afroz, R., Chowdhury, M. A. Z., Khalil, M. I., Hossain, M. S., Rahman, M. A., Rashid, M. H., & Gan, S. H. (2015). Honey has a protective effect against chlorpyrifos-induced toxicity on lipid peroxidation, diagnostic markers and hepatic histoarchitecture. *European Journal of Integrative Medicine*, *7*(5), 525–533. https://doi.org/10.1016/j.eujim.2015.04.004

327. Tenci, M., Rossi, S., Bonferoni, M. C., Sandri, G., Mentori, I., Boselli, C., Cornaglia, A. I., Daglia, M., Marchese, A., Caramella, C., & Ferrari, F. (2017). Application of DoE approach in the development of mini-capsules, based on biopolymers and manuka honey polar fraction, as powder formulation for the treatment of skin ulcers. *International Journal of Pharmaceutics*, *516*(1–2), 266–277. https://doi.org/10.1016/j.ijpharm.2016.10.050

328. Tomasin, R., & Cintra Gomes-Marcondes, M. C. (2011). Oral administration of Aloe vera and honey reduces walker tumour growth by decreasing cell proliferation and

increasing apoptosis in tumour tissue. *Phytotherapy Research*, *25*(4), 619–623. https://doi.org/10.1002/ptr.3293

329. Tomasin, R., De Andrade, R. S., & Gomes-Marcondes, M. C. C. (2015). Oral Administration of *Aloe vera* (L.) *Burm. f.* (Xanthorrhoeaceae) and honey improves the host body composition and modulates proteolysis through reduction of tumor progression and oxidative stress in rats. *Journal of Medicinal Food*, *18*(10), 1128–1135. https://doi.org/10.1089/jmf.2014.0129
330. Uwaydat, S., Jha, P., Tytarenko, R., Brown, H., Wiggins, M., Bora, P. S., & Bora, N. S. (2011). The use of topical honey in the treatment of corneal abrasions and endotoxin-induced keratitis in an animal model. *Current Eye Research*, *36*(9), 787–796. https://doi.org/10.3109/02713683.2010.544441
331. Vaghardoost, R., Mousavi Majd, S. G., Tebyanian, H., Babavalian, H., Malaei, L., Niazi, M., & Javdani, A. (2018). The healing effect of sesame oil, camphor and honey on second degree burn wounds in rat. *World Journal of Plastic Surgery*, *7*(1), 67–71.
332. Veena, K., Shanthi, P., & Sachdanandam, P. (2007). Therapeutic efficacy of Kalpaamruthaa on reactive oxygen/nitrogen species levels and antioxidative system in mammary carcinoma bearing rats. *Molecular and Cellular Biochemistry*, *294*(1–2), 127–135. https://doi.org/10.1007/s11010-006-9252-1
333. Veena, K., Shanthi, P., & Sachdanandam, P. (2006). The biochemical alterations following administration of Kalpaamruthaa and *Semecarpus anacardium* in mammary carcinoma. *Chemico-Biological Interactions*, *161*(1), 69–78. https://doi.org/10.1016/j.cbi.2006.03.003
334. Wahyuningtyas, E. S., Iswara, A., Sari, Y., Kamal, S., Santosa, B., Ishijima, T., Nakatani, T., Putri, I. K., & Nasruddin, N. (2018). Comparative study on Manuka and Indonesian honeys to support the application of plasma jet during proliferative phase on wound healing. *Clinical Plasma Medicine*, *12*(18), 1–9. https://doi.org/10.1016/j.cpme.2018.08.001
335. Wang, F., Zhang, Y. J., Zhou, Y., Li, Y., Zhou, T., Zheng, J., Zhang, J. J., Li, S., Xu, D. P., & Li, H. Bin. (2016). Effects of beverages on alcohol metabolism: potential health benefits and harmful impacts. *International Journal of Molecular Sciences*, *17*(3), 1–12. https://doi.org/10.3390/ijms17030354
336. Wang, H., Bai, J., Chen, G., Li, W., Xiang, R., Su, G., & Pei, Y. (2013). A metabolic profiling analysis of the acute hepatotoxicity and nephrotoxicity of Zhusha Anshen Wan compared with cinnabar in rats using (1)H NMR spectroscopy. *Journal of Ethnopharmacology*, *146*(2), 572–580. https://doi.org/http://dx.doi.org/10.1016/j.jep.2013.01.026
337. Wang, K., Wan, Z., Ou, A., Liang, X., Guo, X., Zhang, Z., Wu, L., & Xue, X. (2019). Monofloral honey from a medical plant, *Prunella Vulgaris*, protected against dextran sulfate sodium-induced ulcerative colitis via modulating gut microbial populations in rats. *Food and Function*, *10*(7), 3828–3838. https://doi.org/10.1039/c9fo00460b
338. Wang, T., Zhu, X. K., Xue, X. T., & Wu, D. Y. (2012). Hydrogel sheets of chitosan, honey and gelatin as burn wound dressings. *Carbohydrate Polymers*, *88*(1), 75–83. https://doi.org/10.1016/j.carbpol.2011.11.069
339. Wang, Y., Li, D., Cheng, N., Gao, H., Xue, X., Cao, W., & Sun, L. (2015). Antioxidant and hepatoprotective activity of vitex honey against paracetamol induced

liver damage in mice. *Food and Function*, *6*(7), 2339–2349. https://doi.org/10.1039/c5fo00345h
340. Xiao, J., Liu, Y., Xing, F., Leung, T. M., Liong, E. C., & Tipoe, G. L. (2016). Bee's honey attenuates non-alcoholic steatohepatitis-induced hepatic injury through the regulation of thioredoxin-interacting protein–NLRP3 inflammasome pathway. *European Journal of Nutrition*, *55*(4), 1465–1477. https://doi.org/10.1007/s00394-015-0964-4
341. Yadav, A., Verma, S., Keshri, G. K., & Gupta, A. (2018). Combination of medicinal honey and 904 nm superpulsed laser-mediated photobiomodulation promotes healing and impedes inflammation, pain in full-thickness burn. *Journal of Photochemistry and Photobiology B: Biology*, *186*(June), 152–159. https://doi.org/10.1016/j.jphotobiol.2018.07.008
342. Yalcinkaya, F. R., Davarci, M., Gokce, A., Guven, E. O., Inci, M., Kerem, M., Ayyildiz, A., Amilkanthwar, R. H., & Kacar, A. (2012). Intraurethral utterbasti application for urethral injury in rats. *Journal of Animal and Veterinary Advances*, *11*(9), 1494–1497. https://doi.org/10.3923/javaa.2012.1494.1497
343. Yaman, T., Yener, Z., & Celik, I. (2016). Histopathological and biochemical investigations of protective role of honey in rats with experimental aflatoxicosis. *BMC Complementary and Alternative Medicine*, *16*(1), 1–11. https://doi.org/10.1186/s12906-016-1217-7
344. Yusof, N., Ainul Hafiza, A. H., Zohdi, R. M., & Bakar, M. Z. A. (2007). Development of honey hydrogel dressing for enhanced wound healing. *Radiation Physics and Chemistry*, *76*(11–12), 1767–1770. https://doi.org/10.1016/j.radphyschem.2007.02.107
345. Yuzbasioglu, M. F., Kurutas, E. B., Bulbuloglu, E., Goksu, M., Atli, Y., Bakan, V., & Kale, I. T. (2009). Administration of honey to prevent peritoneal adhesions in a rat peritonitis model. *International Journal of Surgery*, *7*(1), 54–57. https://doi.org/10.1016/j.ijsu.2008.10.011
346. Zaid, S. S. M., Sulaiman, S. A., Sirajudeen, K. N. M., & Othman, N. H. (2010). The effects of tualang honey on female reproductive organs, tibia bone and hormonal profile in ovariectomised rats - animal model for menopause. *BMC Complementary and Alternative Medicine*, *10*(1), 82. https://doi.org/10.1186/1472-6882-10-82
347. Zaid, S. S. M., Othman, S., & Kassim, N. M. (2014). Potential protective effect of Tualang honey on BPA-induced ovarian toxicity in prepubertal rat. *BMC Complementary and Alternative Medicine*, *14*(1), 1–12. https://doi.org/10.1186/1472-6882-14-509
348. Zárraga-Galindo, N., Vergara-Aragón, P., Rosales-Meléndez, S., Ibarra-Guerrero, P., Domínguez-Marrufo, L. E., Oviedo-García, R. E., Hernández-Ramírez, H., Hernández-Téllez, B., López-Martínez, I. E., Sánchez-Cervantes, I., Vázquez-García, M., & Santiago, J. (2011). Effects of bee products on pentylenetetrazole-induced seizures in the rat. *Proceedings of the Western Pharmacology Society*, *54*(January), 32–39.
349. Zhao, H., Cheng, N., He, L., Peng, G., Liu, Q., Ma, T., & Cao, W. (2018). Hepatoprotective effects of the honey of *Apis cerana* Fabricius on bromobenzene-induced liver damage in mice. *Journal of Food Science*, *83*(2), 509–516. https://doi.org/10.1111/1750-3841.14021
350. Zhao, H., Cheng, N., He, L., Peng, G., Xue, X., Wu, L., & Cao, W. (2017). Antioxidant and hepatoprotective effects of *A. cerana* honey against acute alcohol-

induced liver damage in mice. *Food Research International*, *101*(August), 35–44. https://doi.org/10.1016/j.foodres.2017.08.014
351. Zohdi, R. M., Mukhtar, S. M., Said, S., Azmi, N. A. M., & Ali, A. A. (2014). A comparative study of the wound healing properties of Gelam honey and silver sulfadiazine in diabetic rats. *IECBES 2014, Conference Proceedings - 2014 IEEE Conference on Biomedical Engineering and Sciences: "Miri, Where Engineering in Medicine and Biology and Humanity Meet," December*, 247–250. https://doi.org/10.1109/IECBES.2014.7047495
352. Osuagwu, F.C., Oladejo, O.W., Imosemi, I.O., Aiku, A., Ekpo, O.E., Salami, A.A., Oyedele, O.O., & Akang, E.U. (2004). Enhanced wound contraction in fresh wounds dressed with honey in wistar rats (*Rattus Novergicus*). *West African Journal of Medicine, 23*(2), 114-118.
353. Jalali, F.S.S., Saifzadeh, S., Farshid, A.A., Tajik, H., Bagheri, R., & Mohhammadi, R. (2007). Efficiency assessment of Iranian honey on healing of linea alba following exploratory laparotomy in animal model. *Journal of Animal and Veterinary Advances, 6*(2), 238-241.
354. Hosseini, S.V., Niknshad, H., Fakhar, N., Rezaianzadeh, A., & Mehrabani, D. (2011). The healing effect of mixture of honey, putty, vitriol and olive oil in *Pseudomonas aeroginosa* infected burns in experimental rat model. *Asian Journal of Animal and Veterinary Advances, 6*(6), 572-579.
355. Mobarok Ali, A.T.M., & Al-Swayeh, O.A. (1997). Natural honey prevents ethanol-induced increased vascular permeability changes in the rat stomach. *Journal of Ethnopharmacology, 55,* 231-238.
356. Jalali, F.S.S., Tajik, H., Saifzadeh, S., & Fartash, B. (2007). Topical application of natural urmia honey on experimental burn wounds in the dog: clinical and microbiological Studies. *Asian Journal of Animal and Veterinary Advances*, *2*(3), 133–139. https://doi.org/10.3923/ajava.2007.133.139
357. Jalali, F.S.S., Saifzadeh, S., Tajik, H., & Farshid, A.A. (2007). Experimental evaluation of repair process of burn-wounds treated with natural honey. *Journal of Animal and Veterinary Advances, 6*(2), 179-184.
358. Akanmu, M. A., Olowookere, T. A., Atunwa, S. A., Ibrahim, O., Lamidi, O. F., Adams, P. A., Ajimuda, O., & Adeyemo, L. E. (2011). Neuropharmacological effects of Nigerian honey in mice. *African Journal of Traditional, Complementary and Alternative Medicines, 8*(3), 230-249.
359. Shati, A. A., & Alamri, S. A. (2010). Role of saffron (*Crocus sativus L.*) and honey syrup on aluminum-induced hepatotoxicity. *Saudi Medical Journal, 31*(10), 1106-1113.
360. Sadeghmanesh, F, & Irajsalehi. (2016). Effect of three different types of honey on passive avoidance memory process in the male diabetic rats. *International Journal of Advanced Biotechnology and Research,* 7(3), 2075–2082.
361. Sadek, K., Beltagy, D., Saleh, E., & Abouelkhair, R. (2018). Camel milk and bee honey regulate profibrotic cytokine gene transcripts in liver cirrhosis induced by carbon tetrachloride. *Canadian Journal of Physiology and Pharmacology, 94*(11)*,* 1141-1150. https://doi.org/10.1139/cjpp-2015-0596
362. Aderounmu, A.O., Omonisi, A.E., Akingbasote, J.A., Makanjuola, M., Bejide, R.A., Orafidiya, L.O., & Adelusola, K.A. (2013). Wound-healing and potential anti-

keloidal properties of the latex of *Calotropis procera* (Aiton) Asclepiadaceae in rabbits. *African Journal of Traditional, Complementary, and Alternative Medicine, 10*(3), 574-579. http://dx.doi.org/10.4314/ajtcam.v10i3.28

363. Nisbet, H., Nisbet, C., Yarim, M., Guler, A., & Ozak, A. (2010). Effects of three types of honey on cutaneous wound healing. *Wounds, 22*(11), 275-283.

364. Movaffagh, J., Fazly-Bazzaz, B.S., Yazdi, A.T., Sajadi-Tabassi, A., Azizzadeh, M., Najafi, E. Amiri N., Taghanaki, H.B., Ebrahimzadeh, M.H., & Moradi, A. Wound healing and antimicrobial effects of chitosan-hydrogel/honey compounds in a rat full-thickness wound model. *Wounds, 31*(9), 228-235.

365. Karabulut, E., & Durgun, T. (2004). The use of honey in wound treatment. *Indian Veterinary Journal*, *81*(10), 1108–1110.

366. Al-waili, N.S. (2004). Investigating the antimicrobial activity of natural honey and its effects on the pathogenic bacterial infections of surgical wounds and conjunctiva. *Journal of Medicinal Food, 7*(2), 210–222.

367. Suguna, L., Chandrakasan, G., & Thomas Joseph K. (1992). Influence of honey on collagen metabolism during wound healing in rats. *Journal of Clinical Biochemistry and Nutrition, 13,* 7-12.

368. Suguna, L., Chandrakasan, G., Ramamoorthy, U., & Joseph, K.T. (1993). Influence of honey on biochemical and biophysical parameters of wounds in rats. *Journal of Clinical Biochemistry and Nutrition, 14,* 91-99/

369. Haryanto, H., Urai, T., Mukai, K., Suriadi, S., Sugama, J., & Nakatani, T. (2012). Effectiveness of Indonesian honey on the acceleration of cutaneous wound healing: an experimental study in mice. *Wounds,* 24(4), 110-119.

370. Erejuwa, O. O., Sulaiman, S. A., Wahab, M. S., Sirajudeen, K. N. S., Salleh, M. S. M. D., & Gurtu, S. (2010). Antioxidant protection of Malaysian tualang honey in pancreas of normal and streptozotocin-induced diabetic rats. *Annales d'Endocrinologie*, *71*(4), 291–296. https://doi.org/10.1016/j.ando.2010.03.003

371. Saber, A., Shekidef, M.H., & El-Daharawy, M.H. (2011). Fecal peritonitis in rats. *Journal of Advanced Veterinary Research, 1,* 8-12.

372. Imtara, H., Al-Waili, N., Bakour, M., Al-Waili, W., & Lyoussi, B. (2018). Evaluation of antioxidant, diuretic, and wound healing effect of Tulkarm honey and its effect on kidney function in rats. *Veterinary World, 11*(10), 1491-1499. https://doi.org/10.14202/vetworld.2018.1491-1499

373. Yudaniayanti, I.S., Primarizky, H,., Nangoi, L, & Yuliani, G.A. (2019). Protective effects of honey by bees (*Apis dorsata*) on decreased cortical thickness and bone impact strength of ovariohysterectomized rats as models for menopause. *Veterinary World, 12*(6), 868-876. https://doi.org/10.14202/vetworld.2019.868-876

374. Sayed, S.M., Abou El-Ella, G.A., Wahba, N.M., El Nisr, N.A., Raddad, K., Abd El Rahman, M.F., Abd El Hafeez, A.E., & El Fattah Aamer, A.A. (2009). Immune defense of rats immunized with fennel honey, propolis, and bee venom against induced *Staphylococcal* infection. *Journal of Medicinal Food, 12*(3), 569-575. https://doi.org/10.1089/jmf.2008.0171

375. Abdul-Ghani, A.S., Dadoub N., Muhammad, R., Abdul-Ghani, R., & Qazzaz, M. (2008). Effect of Palestinian honey on spermatogenesis in rats. *Journal of Medicinal Food, 11*(4), 799-802. https://doi.org/10.1089/jmf.2008.0085

376. Hannan, A., Jabeen, K., & Saleem, S. (2015). Effect of different doses of Manuka honey in experimentally induced mouse typhoid. *Pakistan Journal of Pharmaceutical Sciences, 28*(3), 891-902.
377. Eteraf-Oskouei, T., Shaseb, E., Ghaffary, S., & Najafi, M. (2013). Prolonged preconditioning with natural honey against myocardial infarction injuries. *Pakistan Journal of Pharmaceutical Sciences, 26*(4), 681-686.
378. Rakha, M.K., Nabil, Z.I., & Hussein, A.A. (2008). Cardioactive and vasoactive effects of natural wild honey against cardiac malperformance induced by hyperadrenergic activity. *Journal of Medicinal Food, 11*(1), 91-98.
379. Nasrolahi, O., Heidari, R., Rahmani, F., & Farokhi, F. (2012). Effect of natural honey from Ilam and metformin for improving glycemic control in streptozotocin-induced diabetic rats. *Avicenna Journal of Phytomedicine, 2*(4), 212-221.
380. Andritoiu, C.V., Andritoiu, V., Prisacaru, A.I., Cotrutz, C.E., Petreus, T., & Popa, I.M. (2011). Researches regarding the influence of apitherapy diet on leukocyte formula in wistar rats with experimentally CCl_4 induced liver disease. *Veterinary Medicine, 68*(1), 20-29.
381. Gollu, A., Kismet., K., Kilicoglu, B., Erel., S., Gonultas, M.A., Sunay A.E., & Akkus, M.A. (2008). Effect of honey on intestinal morphology, intraabdominal adhesions and anastomotic healing. *Phytotherapy Research, 22*, 1243-1247. https://doi.org/10.1002/ptr.2457
382. Elmenoufy, G.A.M. (2012). Bee honey dose-dependently ameliorates lead acetate-mediated hepatorenal toxicity in rats. *Life Science Journal, 9*(4), 780-788.
383. Abu-Zinadah, O.A., Alsaggaf, S.O., Shaikh Omar, A.M., & Hussein, H.K. (2013). Effect of honey on testicular functions in rats exposed to octylphenol. *Life Science Journal, 10*(1), 979-984.
384. Qamar, M. U., Saleem, S., Toleman, M. A., Saqalein, M., Waseem, M., Nisar, M. A., Khurshid, M., Taj, Z., & Jahan, S. (2018). In vitro and in vivo activity of Manuka honey against NDM-1-producing *Klebsiella pneumoniae* ST11. *Future microbiology*, *13*, 13–26. https://doi.org/10.2217/fmb-2017-0119
385. Azim, M.K., Perveen, H., Mesaik M.A., & Simjee, S.U. (2007). Antinociceptive activity of natural honey in thermal-nociception models in mice. *Phytotherapy Research, 21*, 194-197. https://doi.org/10.1002/ptr.2049
386. Lusby, P.E., Coombes, A.L., & Wilkinson, J.M. (2006). A comparison of wound healing following treatment with *Lavandula* x *allardii* honey or essential oil. *Phytotherapy Research, 20*, 755-757. https://doi.org/10.1002/ptr.1949
387. Kabala-Dzik, A., Szaflarska-Stojko, E., Wojytczka, R.D., Stojko, A., Stojko, R., Pacha, J., & Stojko, J. (2004). Efficiency assessment of antimicrobial activity of honey-balm on experimental burn wounds. *Bulletin of the Veterinary Institute in Pulawy, 48*, 109-112.
388. Oryan, A., & Zaker S.R. (1998). Effects of topical application of honey on cutaneous wound healing in rabbits. *Journal of Veterinary Medicine Series A, 45,* 181-188.
389. Ali, A.T.M.M. (1991). Prevention of ethanol-induced gastric lesions in rats by natural honey, and its possible mechanism of action. *Scandanavian Journal of Gastroenterology, 26,* 281-288.

390. Takzaree, N., Hadjiakhondi, A., Hassanzadeh, G., Rouini, M.R., & Manayi, A. (2015). Synergistic effect of honey and propolis on cutaneous wound healing in rats. *Acta Medica Iranica, 54*(4), 233-239.
391. Applewhite, A.J., Attar, P., Liden, B., & Stevenson, Q. (2015). Gentian violet and methylene blue polyvinyl alcohol foam antibacterial dressing as a viable form of autolytic debridement in the wound bed. *Surgical Technology International XXVI, 26,* 65-70.
392. Attia, W.Y., Gabry, M.S., El-Shaikh, K.A., & Othman, G.A. (2008). The anti-tumor effect of bee honey in Ehrlich ascite tumor model of mice is coincided with stimulation of the immune cells. *The Egyptian Journal of Immunology, 15*(2), 169-183.
393. Yilmaz, N., Nisbet, O., Nisbet, C., Ceylan, G., Hosgor, F., & Dede, O.G. (2009). Biochemical evaluation of the therapeutic effectiveness of honey in oral mucosal ulcers. *Bosnian Journal of Basic Medical Sciences, 9*(4), 290-295.
394. Prakash, A., Medhi, B., Avti, P.K., Saikia, U.N., Pandhi, P., & Khanduja, K.L. (2008). Effect of different dose of manuka honey in experimentally induced inflammatory bowel disease in rats. *Phytotherapy Research, 22,* 1511-1519. https://doi.org/10.1002/ptr.2523
395. Ghasemi, P.A., Koohpayeh, A., & Karimi, I. (2009). Effect of natural remedies on dead space wound healing in wistar rats. *Pharmacognosy Magazine, 5*, 433-436.
396. Iftikhar, F., Arshad, M., Rasheed, F., Amraiz, D., Anwar, P., & Gulfraz, M. (2010). Effects of acacia honey on wound healing in various rat models. *Phytotherapy Research, 24,* 583-586. https://doi.org/10.1002/ptr.2990
397. Asaduzzaman, M., Sohanur Rahman, M., Muedur Rahman, M., Hasan, M., Siddique, M.A.H., Biswas, S., Belal, M.H., Khatun, M., Khan, M.M.H., Rahm,an, M.M., Karim, M.R., & Islam, M.A. (2015). Effects of honey supplementation on hepatic and cardiovascular disease (CVD) marker in streptozotocin-induced diabetic rats. *Journal of Diabetes and Metabolism, 6*(9), 592. https://doi.org/10.4172/2155-6156.1000592
