# Supplementary material for: A Scoping Review of the Evidence for the Medicinal Use of Natural Honey in Animals
Source: Front Vet Sci. 2021 Jan 18;7:618301. doi: 10.3389/fvets.2020.618301 (PMC7847899; doi:10.3389/fvets.2020.618301)
Supplement: Data Sheet 6 — Appendix F. [file Data_Sheet_6.PDF]

Appendix F
Biomedical Research Indications

Gastrointestinal

intraperitoneal adhesions
fecal peritonitis
Helicobacter pylori infection
bromobenzene-induced liver damage
aflatoxin-induced hepatotoxicity
non-alcoholic steatohepatitis
carbon tetrachloride-induced hepatotoxicity
acetaminophen-induced hepatotoxicity
intraabdominal adhesions and anastomotic healing
ammonia-induced gastric lesions
cisplatin-induced hepatotoxicity
ethanol-induced hepatotoxicity
ochratoxin-induced damage in the liver
arsenite-induced liver damage
cinnabar-induced hepatotoxicity
colitis
adhesion prevention and colonic anastomotic healing
cisplatin-induced hepatotoxicity
ulcerative colitis
anastomotic wound healing
liver injury
obstructive jaundice
alcohol-induced liver damage
colonic anastomosis
gastric ulcers
lead-induced hepatotoxicity
chlorpyrifos-induced hepatotoxicity
hepatic disease
paracetamol induced liver damage
acetaminophen-induced hepatotoxicity
metanil-yellow-induced hepatotoxicity
acetylsalicylic acid-induced gastric ulcer
aluminum-induced hepatotoxicity
intestinal anastomotic wound healing
N-ethylmaleimide-induced liver damage
peritoneal adhesions with bacterial peritonitis

inflammatory bowel disease
ulcerative colitis
obstructive jaundice
indomethacin-induced gastric lesions
ischaemia-reperfusion-induced gastric mucosal lesions
methotrexate mediated hepatotoxicity
nonsteroidal anti-inflammatory drugs induced gastric ulcer
ammonia-induced gastric lesions
ethanol-, indomethacin-, and acidified aspirin-induced gastric lesions
indomethacin-induced gastric ulcers
ethanol-induced gastric mucosal damage
bacterial translocation in obstructive jaundice
gastric ulceration
small bowel resection
gastric mucosal injury
anastomosis surgery
diarrhea
acetic acid-induced colitis
ochratoxin A-induced oxidative stress

Nervous system
transient cerebral global ischemia
status epilepticus
pentylenetetrazole-induced seizures
paraquat toxicity
nociception in offspring
nociception
neurological response to stress
memory impairment in diabetes
memory deficits
memory and depressive-behaviour caused by noise stress
lead acetate exposure
kainic acid-induced oxidative stress in brain
kainic acid-induced cortical damage
cerebral hypoperfusion-induced neurodegeneration, cognitive function
arsenite-induced brain damage
anxiety, age-related memory loss
aluminuim chloride-induced neurotoxicity
depression
picrotoxin-induced seizures

Cardiovascular

ventricular hypertrophy, aortic constriction
vasospasm
pulmonary thromboembolism
myocardial infarction
ischemia/reperfusion - induced cardiac arrhythmias
hypertension
congestive heart failure
cerebral hypoperfusion
cardiovascular disease
atherosclerosis
diabetic hypertension
vasomotor dysfunction
epinephrine-induced cardiac disorders

Lymphatic/immune

Toxoplasmosis
Staphylococcus aureus biofilm-associated sinusitis
Staphylococcal infection
Typhoid
Trypanosoma brucei infection
Schistosoma mansoni infection
Salmonella infection
Streptococcus pyogenes infection
rheumatoid arthritis
Klebsiella pneumoniae ST11 systemic infection
Aspergillosis infection
arsenite-induced clastogenicity

Metabolic/endocrine

polycystic ovary syndrome
obesity
menopausal syndrome
glycemic control in diabetes

Urinary/renal

urethral stricture
urethral injury
renal ischemia/reperfusion injury
prostatitis

oxidative stress in the kidney
ochratoxin-induced damage in the kidney
methotrexate (MTX) induced kidney toxicity
melamine toxicity in kidney
lead-induced renal toxicity
diabetic kidney damage
cisplatin-induced nephrotoxicity
carbon tetrachloride-induced nephrotoxicity
amikacin-induced nephrotoxicity
aflatoxin-induced nephrotoxicity
acetaminophen-induced nephrotoxicity

Reproductive
testis injury
pain behaviour in offspring
spermatogenesis
octylphenol toxicity on testes
noise stress effect on testes
nicotine ingestion on testes and testosterone
nicotine effect on testicular functions
infertility due to noise stress
diabetes-induced damages in testes
cigarette smoke-induced testicular damage
cigarette smoke on sexual behaviour and fertility
bisphenol-induced ovarian toxicity
ischemia-reperfusion injury of testis
chemotherapy-induced testicular damage

Wounds
general wound healing
tooth extraction
oral mucosal ulcers
intraoral wound healing

Other Dermatological
paw inflammation
eschar
debridement
ear oedema

atopic dermatitis
paw oedema

Other

tumour proliferation
tumour implantation in surgical wounds
tumour growth
trichlorfon toxicity
tartrazine toxicity
sodium arsenite toxicity
Pseudomonas aeruginosa induced stromal keratitis
peridural fibrosis after laminectomy
paraquat toxicity on lungs
mycotoxin ingestion
menopausal depression under stress
memory in menopause with stress
melamine toxicity
mammary carcinoma
lymphoid leukemia
lipopolysaccharide-induced endotoxemia
lead-induced anemia
lead acetate contamination
keratitis
epidural fibrosis following laminectomy
dry eyes
cornea alkali burns
carrageenan-induced inflammation
carbon tetrachloride-induced hematological changes
carbon tetrachloride toxicity
cadmium toxicity
breast cancer
asthma
arthritis
anemia
allergic asthma
alkali injury to the eye
alcohol intoxication
acute blood loss
foreign-body granuloma
cyclophosphamide genotoxicity

corneal abrasions
endotoxin-induced keratitis
bacterial conjunctivitis
formaldehyde-induced arthritis
